# Vanadium-based cathode materials for Zn-, Al-, Mg-, and NH_4_^+^-based aqueous rechargeable batteries: modification strategies and energy-storage mechanisms

**DOI:** 10.1039/d6sc04339a

**Published:** 2026-07-29

**Authors:** Xunxiang Jia, Xiaoyue Zhang, Xiangyang Li, Wenhaotian Zhu, Xinxin Cui, Weiji Li, Mengchuan Lai, Zekai Zhu, Yumeng Lu, Xuefeng Ren, Xifei Li, Anmin Liu, Chong Peng

**Affiliations:** a State Key Laboratory of Fine Chemicals, School of Chemical Engineering, Ocean and Life Sciences, Leicester International Institute, Dalian University of Technology Panjin 124221 China renxuefeng@dlut.edu.cn liuanmin@dlut.edu.cn pengchong@dlut.edu.cn; b School of Materials Science and Engineering, Xi'an University of Technology Xi'an 710048 China xfli@xaut.edu.cn; c State Key Laboratory for Manufacturing Systems Engineering, School of Mechanical Engineering, Xi'an Jiaotong University Xi'an 710049 China

## Abstract

Aqueous rechargeable batteries employing vanadium-based cathodes, including aqueous zinc-ion batteries (AZIBs), aqueous aluminum-ion batteries (AAlIBs), aqueous magnesium-ion batteries (AMgIBs), and aqueous ammonium-ion batteries (NH_4_^+^-IBs), have gained increasing attention as promising alternatives to conventional lithium-ion batteries (LIBs) because of their improved safety, cost effectiveness, and environmental compatibility. In this background, vanadium-based cathode materials, owing to their multivalent redox capabilities (V^2+^ to V^5+^), diverse architectures (*e.g.*, layered, tunnel-like, or three-dimensional frameworks), and elevated theoretical capacities, perform even more outstandingly. These characteristics can help overcome challenges, such as narrow electrochemical stability windows (∼1.23 V), sluggish ion diffusion, and cathode dissolution. A comprehensive analysis of vanadium oxides, vanadates, phosphates, sulfides, nitrides, and their composites across these aqueous battery platforms is given in this review. Various performance-enhancing strategies like ion/water intercalation, surface engineering, defect engineering, and heterostructure design were emphasized. Additionally, this review outlines future research directions for advancing these aqueous ion battery technologies toward practical applications in grid-scale storage and portable electronics. Focusing on the materials design and mechanism study, this review discusses the key challenges and opportunities for vanadium-based materials as a promising cathode in aqueous rechargeable batteries, both for fundamental research and practical applications. This study serves to promote future efforts toward the development of vanadium-based materials and related materials for applications in aqueous rechargeable batteries.

## Abstract 2

This review systematically discusses the interconnections between modification strategies, ion–host interactions, and electrochemical performance of vanadium-based cathodes for aqueous rechargeable batteries.

## Graphical abstract

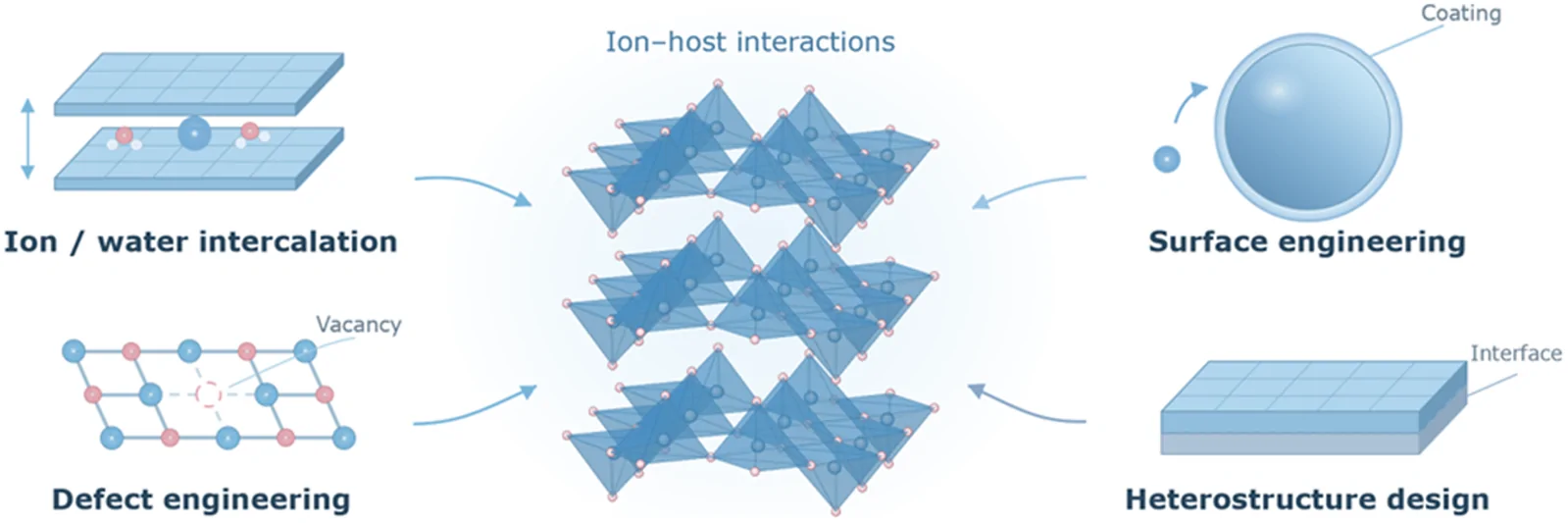

## 1. Introduction

Fundamentally limited by safety concerns regarding flammable organic electrolytes and a dependence on scarce resources,^1–3^ current lithium-ion batteries (LIBs) are increasingly unable to meet the escalating demands of renewable energy storage and electrified transportation.^4–6^ High energy density, long cycle stability, and scalable manufacturability, combined with safety and sustainability, are the characteristics urgently needed for modern energy storage.^7,8^ Fortunately, aqueous metal batteries are promising alternatives due to their non-flammable water-based electrolytes and earth-abundant electrode materials. They offer inherently safer, more sustainable, and cost-effective energy storage.^9,10^ For example, recent research highlights that aqueous metal-ion systems benefit from these attributes, leveraging simple, low-cost manufacturing for grid-scale applications.^11–14^

Aqueous rechargeable batteries can be classified according to their charge carriers. In this review, we focus on aqueous zinc-ion batteries (AZIBs), aqueous aluminum-ion batteries (AAlIBs), aqueous magnesium-ion batteries (AMgIBs), and aqueous ammonium-ion batteries (NH_4_^+^-IBs) that employ vanadium-based cathode materials. Among them, AZIBs, AAlIBs, and AMgIBs involve metal-ion charge carriers with relatively strong ion–host interactions, whereas NH_4_^+^-IBs are based on a monovalent molecular ion and are therefore discussed as an important aqueous rechargeable battery system rather than as a multivalent-ion system. Each type of aqueous battery has its own unique advantages. AZIBs have a high theoretical capacity of 820 mAh g^−1^ and a low redox potential of −0.76 V.^15–18^ AAlIBs draw on aluminum's exceptional three-electron redox mechanism; Al metal offers theoretical gravimetric and volumetric capacities of 2980 mAh g^−1^ and 8046 mAh cm^−3^, respectively.^19^ Coupled with aluminum's vast crustal abundance and industrial recyclability, these multi-electron reactions promise very high energy density at low cost.^20^

AMgIBs similarly leverage the divalent Mg^2+^ chemistry: Mg has a high volumetric capacity (3833 mAh cm^−3^) as a metal anode.^21^ In practice, Mg systems can be made compact with reduced dendrite risk,^22^ although the sluggish diffusion kinetics of solvated Mg^2+^ remain a major research challenge.^23,24^ Furthermore, AIBs use the NH_4_^+^ cation (molecular mass 18 g mol^−1^), which is even lighter than protonated water (H_3_O^+^) and has a small hydrated radius.^25,26^ The tetrahedral NH_4_^+^ can rapidly diffuse and intercalate into host lattices,^27,28^ offering high ionic conductivity and excellent rate capability; importantly, NH_4_^+^-based cells avoid all metal corrosion issues and are intrinsically non-flammable and low-cost.^29^ Within this landscape, vanadium-based cathode materials have attracted intense interest for all these aqueous multivalent systems. Common positive electrode materials based on vanadium include vanadium oxides, vanadium nitrides, vanadium sulfides, and vanadium phosphates, as shown in Fig. 1.^30^

**Fig. 1:**
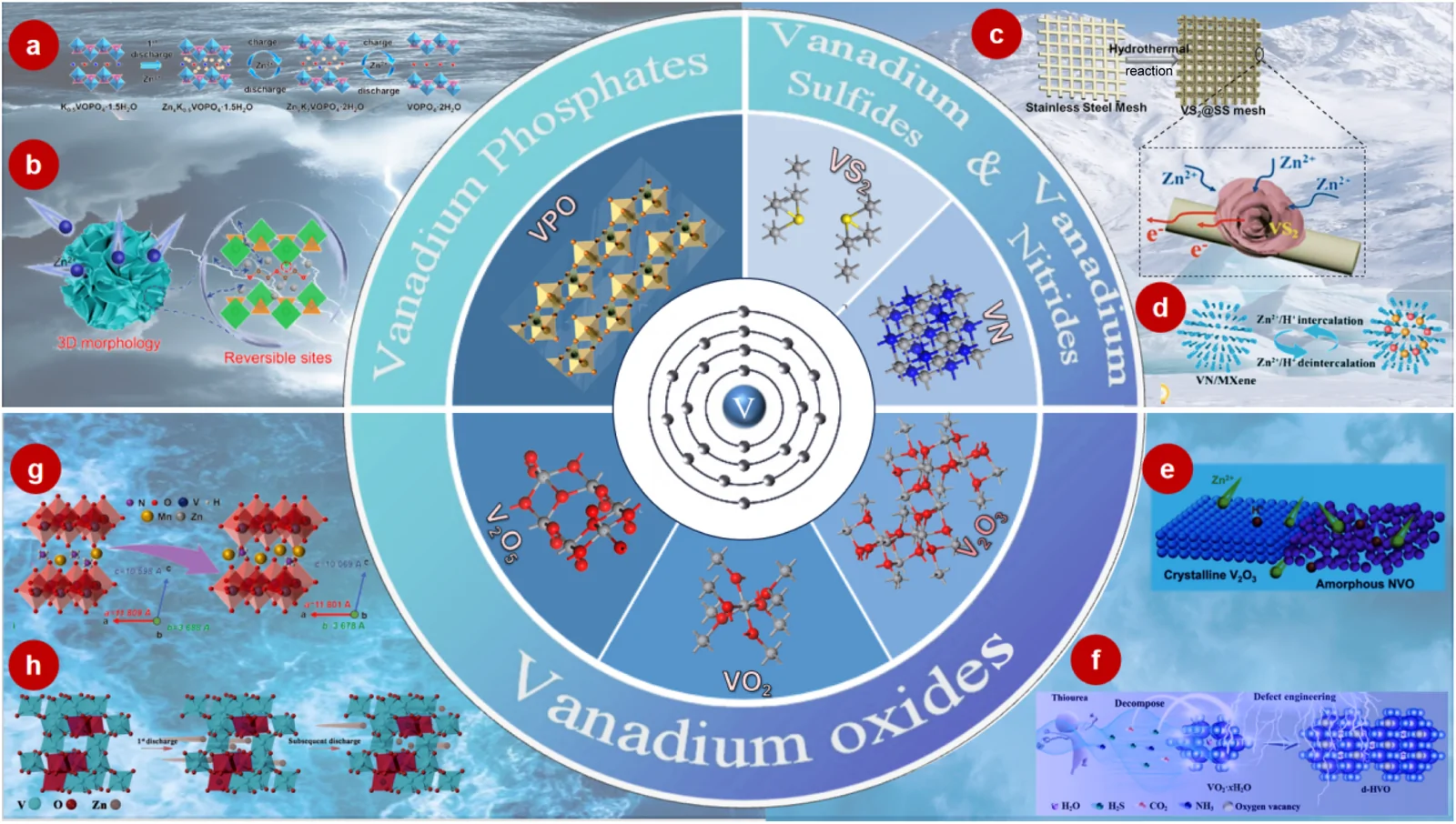
Classification of vanadium-based cathode materials and representative applications in aqueous metal-ion batteries: (a and b) vanadium phosphates; (c) vanadium sulfides; (d) vanadium nitrides; and (e–h) vanadium oxides.^31–40^ Adapted from ref. 31–33, 35, 36 and 38 with permission from Elsevier, copyright 2025, 2025, 2025, 2024, 2024 and 2022, respectively; ref. 34 and 37 with permission from the Royal Society of Chemistry, copyright 2025 and 2019, respectively; ref. 39 with permission from the American Chemical Society, copyright 2025; and ref. 40 with permission from Springer Nature, copyright 2021.

Vanadium oxides and related compounds exhibit a wealth of oxidation states (V^2+^ through V^5+^) and structural motifs (layered, tunnel-like, amorphous, *etc.*).^41–44^ These versatile frameworks can accommodate various ions while enabling multi-electron redox processes.^45^ For instance, vanadium oxides are known to support multiple-electron charge-transfer reactions, which significantly boost capacity and energy density.^46,47^ Indeed, recent studies report that Zn–vanadium cells achieve high capacity and cycling stability through self-adaptive proton intercalation alongside Zn^2+^ insertion and access to several V redox couples.^48^ In general, the combination of abundant vanadium, rich redox chemistry, and flexible crystal structures makes vanadium-based cathodes highly attractive for next-generation aqueous batteries. This review thus focuses on vanadium oxide and vanadate cathode materials for aqueous Zn^2+^, Al^3+^, Mg^2+^, and NH_4_^+^ batteries, surveying how their structural diversity and electrochemical properties can be optimized to maximize capacity, rate performance, and cycle life.

Although several reviews have summarized vanadium-based materials for aqueous rechargeable batteries, most existing studies primarily catalog material classes or report electrochemical performance metrics, with limited emphasis on how specific material modification strategies regulate ion–host interaction mechanisms and structure–property relationships.^15,49–51^ In contrast, this review distinguishes itself by systematically correlating material modification strategies, including intercalation chemistry, defect engineering, surface/interface regulation, and heterostructure construction, with their underlying energy-storage mechanisms, thereby providing a mechanism-driven interpretation of prior studies rather than a descriptive compilation.^15,49^ Moreover, rather than focusing on a single aqueous battery chemistry, this review adopts a comparative, charge-carrier-informed perspective, critically analyzing how vanadium-based cathode design principles diverge among Zn^2+^, NH_4_^+^, Mg^2+^, and Al^3+^ systems, reflecting fundamental differences in charge density, solvation behavior, and diffusion kinetics.^10,19,52–54^ By explicitly linking charge-carrier characteristics to framework selection, interlayer chemistry, and modification routes, this work establishes generalized yet carrier-specific design principles that are not comprehensively articulated in prior reviews.^15,25,55^ Importantly, this review goes beyond performance comparison by identifying common mechanistic bottlenecks across aqueous vanadium-based systems, such as vanadium dissolution, sluggish multivalent-ion kinetics, and lattice instability, and tracing them to their physicochemical origins, including unstable vanadium valence states, strong ion–oxygen electrostatics, and strain accumulation during repeated insertion/extraction.^17,49,50^ On this basis, feasible and system-dependent future research directions are proposed, emphasizing mechanism-guided material design, multifunctional hybrid architectures, and electrolyte–cathode co-engineering.^10,15,19^ As such, this review provides a unified mechanistic framework that connects modification strategies, energy-storage mechanisms, and charge-carrier chemistry, thereby offering original analytical insight and practical guidance for the rational design of next-generation vanadium-based cathodes for aqueous rechargeable batteries.

Although aqueous rechargeable batteries encompass a wide range of charge carriers, including monovalent (Li^+^, Na^+^, and K^+^) and alkaline-earth ions (Mg^2+^ and Ca^2+^), this review concentrated primarily on Zn^2+^-, NH_4_^+^-, and Al^3+^-based systems employing vanadium-based cathodes. This selection reflects both the maturity of these chemistries and their strong mechanistic relevance to vanadium redox behavior. Monovalent aqueous batteries generally exhibit weaker electrostatic interactions and faster diffusion kinetics, leading to fundamentally different structure–property relationships compared with multivalent systems. As a result, the modification strategies and mechanistic challenges central to vanadium-based cathodes in multivalent aqueous batteries, such as strong ion–host coupling and lattice strain, are less representative in monovalent systems and are therefore beyond the scope of this review. While Mg^2+^ and Ca^2+^ aqueous batteries are emerging research directions, their application with vanadium-based cathodes remains comparatively limited due to severe kinetic constraints, strong solvation effects, and a lack of well-established reversible storage mechanisms. Consequently, insufficient systematic data currently exist to support a comprehensive mechanistic comparison with Zn^2+^- or Al^3+^-based systems. In contrast, Zn^2+^-, NH_4_^+^-, and Al^3+^-based aqueous batteries have demonstrated diverse yet well-documented interaction mechanisms with vanadium-based materials, including Zn^2+^ intercalation, hydrogen-bond-mediated NH_4_^+^ storage, and surface-dominated Al^3+^ redox processes. These systems therefore provide a robust platform for elucidating how material modification strategies regulate multivalent ion storage behavior.^56^

## 2. Vanadium-based cathodes for AZIBs

AZIBs are considered promising alternatives to LIBs due to their intrinsic advantages: non-flammable electrolytes, abundant zinc resources, and eco-friendliness. These features result in enhanced safety and lower costs. In addition, zinc anodes deliver high theoretical capacity (820 mAh g^−1^), low redox potential (−0.76 V *vs.* SHE), and stable plating in mild aqueous media. However, the positive electrode has not been commercialized because of cathode limitations, such as low capacity, sluggish Zn^2+^ kinetics, structural instability, and dissolution.^57^

To address these limitations of the cathode, vanadium-based compounds stand out as cathodes due to their multivalent redox activity (V^2+^ to V^5+^), enabling high capacities (>300 mAh g^−1^) and versatile structures (layered and tunnel-like) for reversible Zn^2+^ intercalation and fast transport. Currently, researchers boost the performance of vanadium-based compounds through ion/water pre-intercalation, defect engineering, and heterostructures to boost conductivity, mitigate dissolution, and achieve long cycle life. Fig. 2 provides a timeline of key developments in vanadium-based cathodes for AZIBs from 2015 to 2025.

**Fig. 2:**
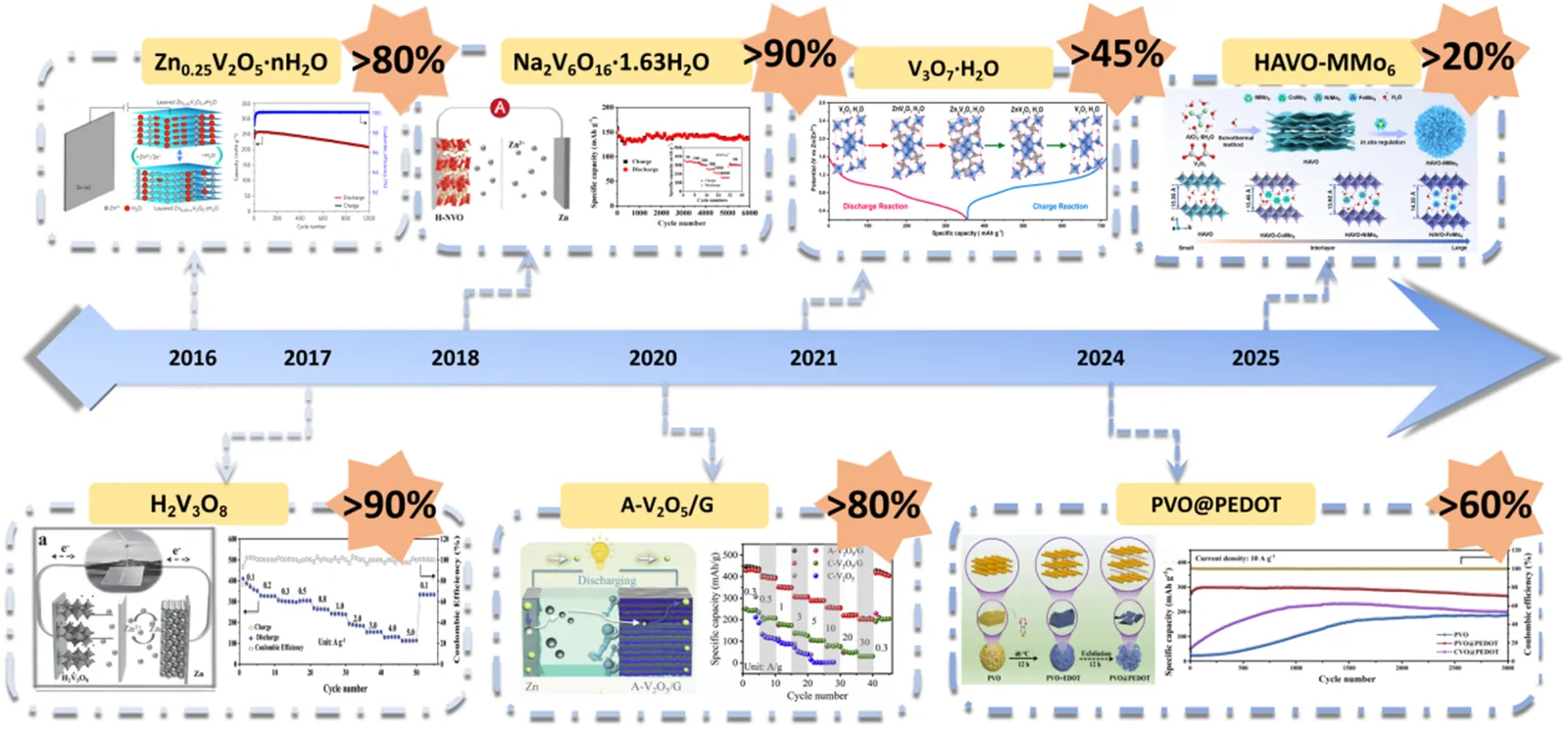
Chronological advancements in vanadium-based cathode materials for AZIBs. In 2016, a vanadium oxide bronze pillared by interlayer Zn^2+^ ions and water (Zn_0.25_V_2_O_5_·*n*H_2_O) was reported as a high-performance positive electrode. In 2017, H_2_V_3_O_8_ nanowires were introduced in a Zn(CF_3_SO_3_)_2_ electrolyte system. By 2018, Na_2_V_6_O_16_·1.63H_2_O nanowires enabled the fabrication of durable batteries. In 2020, ultrathin V_2_O_5_/graphene heterostructures (6 nm thick) enhanced stability. In 2021, V_3_O_7_·H_2_O nanoarrays with high mass loadings were synthesized facilely. In 2024, conductive polymer-intercalated V_2_O_5_ ultrathin nanosheets were developed. In 2025, 3D nanoflower aluminum vanadate with polyoxometalates (HAVO-MMo_6_-X) was fabricated *via* the solvothermal method.^54,58–66^ Adapted from ref. 54, 59 and 65 with permission from Wiley-VCH, copyright 2022, 2017 and 2023, respectively; ref. 58 with permission from Springer Nature, copyright 2016; ref. 60 with permission from Elsevier, copyright 2019; ref. 61–63 with permission from the Royal Society of Chemistry, copyright 2018, 2020 and 2022, respectively; and ref. 64 and 66 with permission from the American Chemical Society, copyright 2024 and 2025, respectively.

This subsection reviews the application of these materials in AZIBs, categorized into oxides, vanadates, phosphates, sulfides, nitrides, and composites, emphasizing reaction mechanisms, performance metrics, and future directions for practical implementation.

### 2.1 Vanadium-based oxides

Vanadium-based oxides feature multi-electron redox reactions and a rich variety of oxidation states (V^2+^, V^3+^, V^4+^, and V^5+^), which endow them with high capacity (>300 mAh) and large ionic transport channels; as a result, vanadium-based oxides are good materials as the cathode material for AZIBs. Moreover, V–O has multiple structural forms, such as VO_4_ tetrahedra, VO_5_ square pyramids, triangular bipyramids, and VO_6_ octahedra. These different polyhedra of vanadium oxides can combine to form chain-like, layer-like or three-dimensional structures. This leads to a variety of different structures for vanadium-based oxides. At present, relevant research mainly improves the structures of vanadium-based cathode materials through intercalation strategies, surface engineering, defect engineering, heterostructures, and other methods to enhance their performance. The following subsections review representative vanadium-based oxide cathodes for AZIBs, focusing on intercalation strategies, surface engineering, defect engineering, and heterostructure construction.^67^

#### 2.1.1 Intercalation strategies

Vanadium-based oxides typically exhibit layered or tunnel-type crystal structures with relatively large interstitial spaces, allowing the insertion of ions, such as Zn^2+^, Na^+^, K^+^, and H_2_O molecules. These intercalations can effectively modulate the interlayer structure and enhance the electrochemical performance of vanadium-based cathode materials.

For example, Kundu *et al.* synthesized Zn_0.25_V_2_O_5_·*n*H_2_O *via* a hydrothermal method by co-intercalating Zn^2+^ ions and water molecules into the interlayer space of V_2_O_5_.^58^ The resulting material features two redox-active centers (vanadium) and adopts a layered structure composed of divalent cations and interlayer water, enabling fast and reversible ion transport. The excellent rate capability of Zn_0.25_V_2_O_5_·*n*H_2_O therefore arises not only from its high crystallinity, but also from the cooperative effect of Zn^2+^/H_2_O co-intercalation,^68^ which minimizes framework distortion and maintains low mechanical stress during repeated cycling.

Structural characterization revealed that the product consists of two-dimensional, ultra-long, single-crystalline nanoribbons. Scanning electron microscopy (SEM) images show that the material consists of flat, ribbon-like structures spanning several tens of micrometers. And their widths are 100–150 nm and the thicknesses are about 10–20 nm. Transmission electron microscopy (TEM) further confirms their anisotropic geometry, planar morphology, and single-crystal nature. High-resolution transmission electron microscopy (HRTEM) measurements reveal that the lattice spacing is 0.537 nm, indicating that the nanoribbons preferentially grow along the *b*-axis direction.

X-ray diffraction (XRD) analysis shows that the pattern is dominated by (00l) reflections. This indicates a high degree of crystalline orientation, which is due to the high aspect ratio of the nanoribbons. Additionally, this structure is also composed of a double-layer two-dimensional framework, which is very similar to the δ-phase V_2_O_5_.

During electrochemical cycling, the Zn^2+^ ions are reversibly inserted into and extracted from the interlayer galleries. X-ray photoelectron spectroscopy (XPS) confirms that the interaction between intercalated Zn^2+^ and the oxide framework is relatively weak due to only moderate bond rearrangement and low structural stress. These features are useful for achieving the excellent rate capability and long-term cycling stability of the electrode. Notably, the interlayer spacing expands reversibly from 10.8 Å to 12.9 Å after intercalation of water molecules. This facilitates ion transport.

Electrochemical testing in a full cell configuration, using 1 M ZnSO_4_ as the electrolyte and metallic zinc as the anode, demonstrates outstanding high-rate performance. The electrode delivers a specific discharge capacity of 282 mAh g^−1^ at 1C (1C = 300 mA g^−1^). At 15C and 20C, the corresponding capacities are 223 and 183 mA h g^−1^, with discharge times of approximately 3 and 2 min, respectively. Moreover, after 100 charge–discharge cycles, vanadium dissolution into the electrolyte was found to be negligible (<0.01%), and Zn^2+^ remained stably retained within the electrode. The electrochemical testing results indicate excellent structural integrity and cycling stability.^58^

Seonwoo *et al.* through the plasma-assisted hydrothermal synthesis illustrated the WiVO nanosheet clusters (Fig. 3A).^69^ The vanadium and oxygen elements are uniformly distributed throughout the clusters (Fig. 3B). To demonstrate the effect of water molecules, they performed the density functional theory (DFT) calculations on anhydrous V_2_O_5_ and V_2_O_5_·H_2_O (Fig. 3C). The results of the DFT calculations indicate that charge transfer occurs between the Zn and intercalated H_2_O rather than between intercalated Zn and the V_2_O_5_ layers, which suggests that the intercalated H_2_O can improve Zn diffusion.^69^

**Fig. 3:**
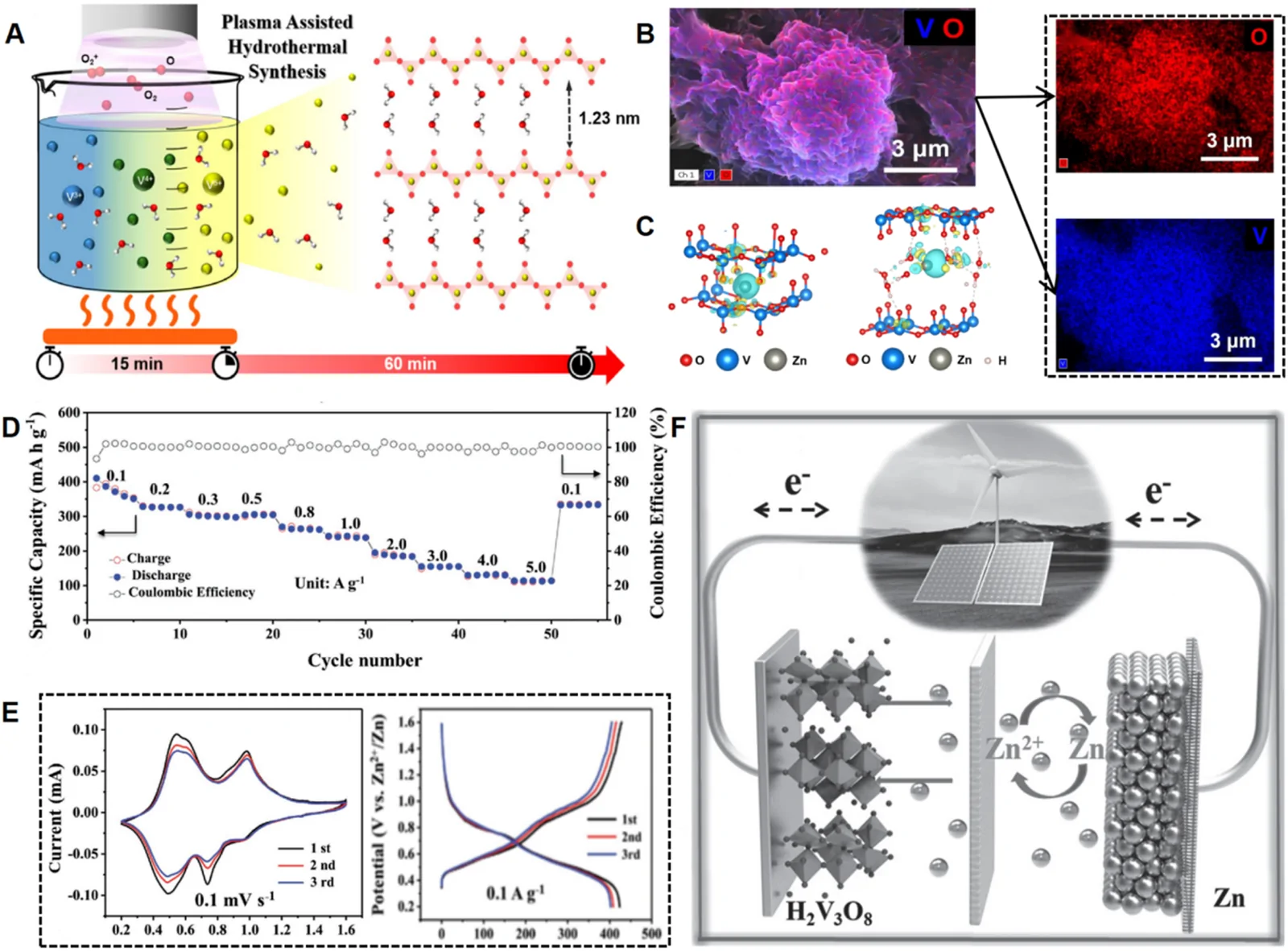
(A) Schematic of PAHT synthesis of WiVO 3D nanosheet clusters; (B) SEM-EDS mapping of WiVO 3D nanosheet clusters; (C) charge density distributions of anhydrous V_2_O_5_ and V_2_O_5_·H_2_O;^69^ (D) rate performance; (E) initial cyclic voltammetry (CV) curves at 0.1 mV s^−1^ (0.2–1.6 V) for Zn//H_2_V_3_O_8_ and initial charge/discharge profiles at 0.1 A g^−1^. (F) Schematic of the Zn//H_2_V_3_O_8_ battery.^59^ Adapted from ref. 59 and 69 with permission from Wiley-VCH, copyright 2017 and 2025, respectively.

Pan *et al.* also designed a vanadium oxide hydrate with water molecules embedded in layered vanadium compounds, namely H_2_V_3_O_8_. This material consists of V_3_O_8_ layers interconnected by hydrogen bonds, where each layer is composed of VO_6_ octahedra and VO_5_ trigonal bipyramids. X-ray diffraction (XRD) analysis reveals twelve distinct diffraction peaks, which indicate that the structure is highly crystalline and well-defined orthorhombic. Scanning electron microscopy (SEM) images show that the nanowires are uniformly dispersed with diameters ranging from 80 to 120 nm and lengths extending over several micrometers. The material exhibits a smooth surface, and high-resolution transmission electron microscopy (HRTEM) images display clear lattice fringes with a spacing of 0.341 nm, further confirming its orthorhombic phase.^59^

In the electrochemical performance evaluation, CR2016 coin-type cells were assembled with zinc metal as the anode. At a current density of 0.1 A g^−1^, the first three discharge capacities respectively reached 423.8, 408.4, and 402.1 mAh g^−1^. After 30 cycles, the capacity retention remained 84.5%, and the energy density was approximately 250 Wh kg^−1^. Furthermore, under a high-rate condition of 5.0 A g^−1^, the battery exhibited an initial discharge capacity of 173.6 mAh g^−1^. Moreover, it retained a capacity of 136.1 mAh g^−1^ after 1000 cycles (Fig. 3D).

For understanding the zinc-ion insertion/extraction mechanism in H_2_V_3_O_8_ (Fig. 3E and F), the team used XRD, TEM, XPS, and Raman spectroscopy. The XRD results combined with Bragg's law reveal that the interlayer spacing of the (200) crystal plane expanded during discharge and contracted during charge, which was attributed to the reversible intercalation of Zn^2+^ ions between the V_3_O_8_ layers (Fig. 3D). This charging and discharging process was accompanied by a redox reaction between V^5+^ and V^4+^. Notably, a new diffraction peak appeared at 0.6 V and disappeared at 1.2 V, indicating the formation and subsequent disappearance of a Zn_*x*_H_2_V_3_O_8_ intermediate phase during cycling. Raman spectroscopy further confirmed that the structural changes caused by Zn^2+^ insertion and extraction were minimal during the first two cycles, indicating that the material possesses good structural reversibility.^59^

Li *et al.* developed a vanadium-based cathode material based on K_0.486_V_2_O_5_ with a large interlayer spacing (∼0.95 nm) and high capacity. The research group investigated the microstructure of the KVO material using XRD, SEM, TEM, and thermogravimetric analysis (TGA) techniques. The strong diffraction peaks observed in the XRD patterns indicate high crystallinity of the sample. SEM and TEM images revealed that KVO exhibits a uniform rod-like morphology, with widths below 50 nm and lengths of only a few hundred nanometers. High-resolution TEM further confirmed a lattice spacing of approximately 0.75 nm.^70^

KVO nanorods exhibited excellent electrochemical performance as the cathodes for AZIBs. At a current density of 0.1 A g^−1^, its specific capacity is as high as 218 mAh g^−1^, and it still retains 92% of its capacity after 500 cycles. Its good rate capability benefits from the large interlayer spacing and one-dimensional single-crystalline nanowire structure, while its cycling stability is enhanced by the suppression of vanadium dissolution in the optimized 15 m ZnCl_2_ electrolyte.

Mechanism analysis indicated that the zinc storage process of this material was accompanied by multi-step intercalation of Zn^2+^ and reversible redox reactions of V^5+^/V^4+^. Furthermore, the pseudo capacitance effect dominates the overall energy storage behavior, indicating that this material has rapid kinetic characteristics.^70^ Furthermore, their team also prepared Na_2_V_6_O_16_·1.63H_2_O nanowire cathode materials through a hydrothermal method. It has a high specific capacity of 352 mAh g^−1^ at a current density of 50 mA g^−1^. Additionally, this cathode also had an extremely long cycle life. After 6000 cycles at 5000 mA g^−1^, the capacity retention rate still reached 90%.^71^ Another typical intercalation-type vanadium-based cathode material is Na_2_V_6_O_16_·1.63H_2_O nanowires synthesized by Hu *et al.* through a hydrothermal method. This material features a layered structure, with a large interlayer spacing that facilitates the reversible intercalation of Zn^2+^. Electrochemical tests show that it provides a specific capacity of up to 270 mAh g^−1^ at 0.1 A g^−1^ and retains approximately 90% of its capacity after 6000 cycles, demonstrating excellent cycling stability. Its superior performance mainly benefits from the stable layered structure, good electronic/ionic conductivity, and the synergistic effect of pre-intercalated Na^+^ and crystalline water in regulating the ion migration process within the structure.^71^ The Sambandam group used a one-dimensional layered nanomaterial, K_2_V_6_O_16_·2.7H_2_O (KVO), which featured a uniform rod-like structure. Its nanorods are less than 50 nm in width. The material demonstrated promising electrochemical performance: its specific capacity can meet 230 mAh g^−1^ after 90 cycles at a current density of 1 A g^−1^. After 170 cycles, the capacity slightly decreased to 222.8 mAh g^−1^. When the current density was increased to 3 A g^−1^, the capacity declined to 205.6 mAh g^−1^, and after 470 cycles, a stable capacity of 177.4 mAh g^−1^ was retained, indicating good long-term cycling stability and rate performance.^61^ In addition, Liu *et al.* synthesized Na^+^-intercalated VO_2_·0.3H_2_O, in which the inserted sodium ions served to expand the tunnel structure and enhance structural stability during cycling. In the Zn//VO_2_·0.3H_2_O battery, the material delivered an initial discharge capacity of 397 mAh g^−1^ at a current density of 0.2 A g^−1^ and retained 301 mAh g^−1^ after 110 cycles, demonstrating good capacity retention and structural robustness.^72^ Choi *et al.* synthesized two-dimensional and three-dimensional vanadium oxide nanosheet clusters with intercalated water *via* a novel plasma-assisted hydrothermal method. The presence of interlayer water expanded the lattice spacing by 1.23 nm, significantly enhancing zinc-ion diffusion kinetics. In electrochemical performance tests, the material delivered a high specific capacity of 324 mAh g^−1^ at 0.1 A g^−1^, and retained 95.8% of its capacity after 4000 cycles at a high current density of 10 A g^−1^, demonstrating outstanding cycling stability and rate performance.^69^ The Tang group synthesized carbon-intercalated vanadium oxide (CNVO) as a cathode material for zinc-ion batteries by inserting polyaniline (PANI) into vanadium oxide and then carbonizing it. This process transformed the structure from tetragonal (NVO) to orthorhombic, and the lattice spacing changed from 0.47 nm (NVO) to 0.45 nm (CNVO). CNVO exhibited a high specific capacity of 386 mAh g^−1^ at 0.1 A g^−1^ and retained 200 mAh g^−1^ at 10 A g^−1^, demonstrating excellent cycling stability with performance remaining stable after over 2000 cycles. The performance improvement was attributed to the enhanced electrical conductivity due to nitrogen doping and the mixed valence state of vanadium (V^4+^/V^5+^), making it suitable for flexible wearable electronic devices (Fig. 5C–E).^68^ Hu *et al.* developed a hydrated vanadium oxide cathode co-pre-intercalated with Ti^4+^ and K^+^ (TiK-VOH). It delivered a specific capacity of 393.4 mAh g^−1^ at 0.2 A g^−1^ and exhibited 94% capacity retention after 2000 cycles at 10 A g^−1^. It also demonstrated excellent cycling stability and rate performance in ZIBs, with a Zn^2+^ diffusion coefficient reaching 10^−9^ to 10^−10^ cm^2^ s^−1^.^73^ Additionally, Yilong Xu *et al.* proposed a superfast-activated NH_4_ intercalated vanadium oxide cathode material (E-VON), which reduced the activation time to less than 24 hours and decreased the impedance to 57.0 Ω (compared to 88.0 Ω for traditional VON). It achieved outstanding rate performance and cycling stability through a mixed charge storage mechanism, with the diffusion contribution increasing from 50% to 70%.^74^

Junwei *et al.* designed VN_0.9_O_0.15_ (Fig. 5A), which has good stability, as indicated by XRD (Fig. 4A).^75^ Yan's team synthesized N-V_2_O_3_@N–C according to the steps shown in Fig. 4B and 5B.^76^ The material can achieve a capacity of 349.0 mAh g^−1^ at 0.1 A g^−1^. Chang *et al.* synthesized VNO/graphene *via* a facile one-step pyrolysis process (Fig. 4F). The elements, C, V, O, and N, of VNO/graphene are shown in Fig. 4C. The elements C and O are distributed uniformly throughout the nanosheets. However, the V and N elements, along with a little O, are densely distributed at the nanoparticles in the high-angle annular dark-field image, indicating that nanoparticles are VN-doped with a large amount of O. CV curves of the material show that the peak of the material is at 1.2 V, but the peak of VNO is at about 1.3 V. The CV curves indicate that Zn^2+^ can more easily diffuse in the VNO/graphene (Fig. 4D). In electrochemistry, in contrast, at 0.2 A g^−1^ the capacity of VNO and VNO/graphene is similar, but with an increase in electron density the capacity of VNO/graphene is larger than the capacity of VNO because the graphene can maximize the exposure of active sites (Fig. 4E).^77^ By employing a polyvinylpyrrolidone (PVP) pre-intercalation and V^4+^/V^5+^ dual valence state regulation strategy, Mi *et al.* developed a high-performance calcium vanadium oxide (PVP–CaVO) cathode material for AZIBs (Fig. 5A–D). This method expanded the lattice spacing to facilitate Zn^2+^ diffusion and significantly enhanced the electrochemical performance. PVP–CaVO exhibited a high specific capacity of 263 mAh g^−1^ at 0.1 A g^−1^ and retained approximately 80% of its capacity after 2000 cycles at 10 A g^−1^. The electrochemical tests show excellent cycling stability and rate performance.^78^ Another similar material is H_2_V_3_O_8_/rGO-100, synthesized by Gan and his team, as shown in Fig. 5F–I.^79^

**Fig. 4:**
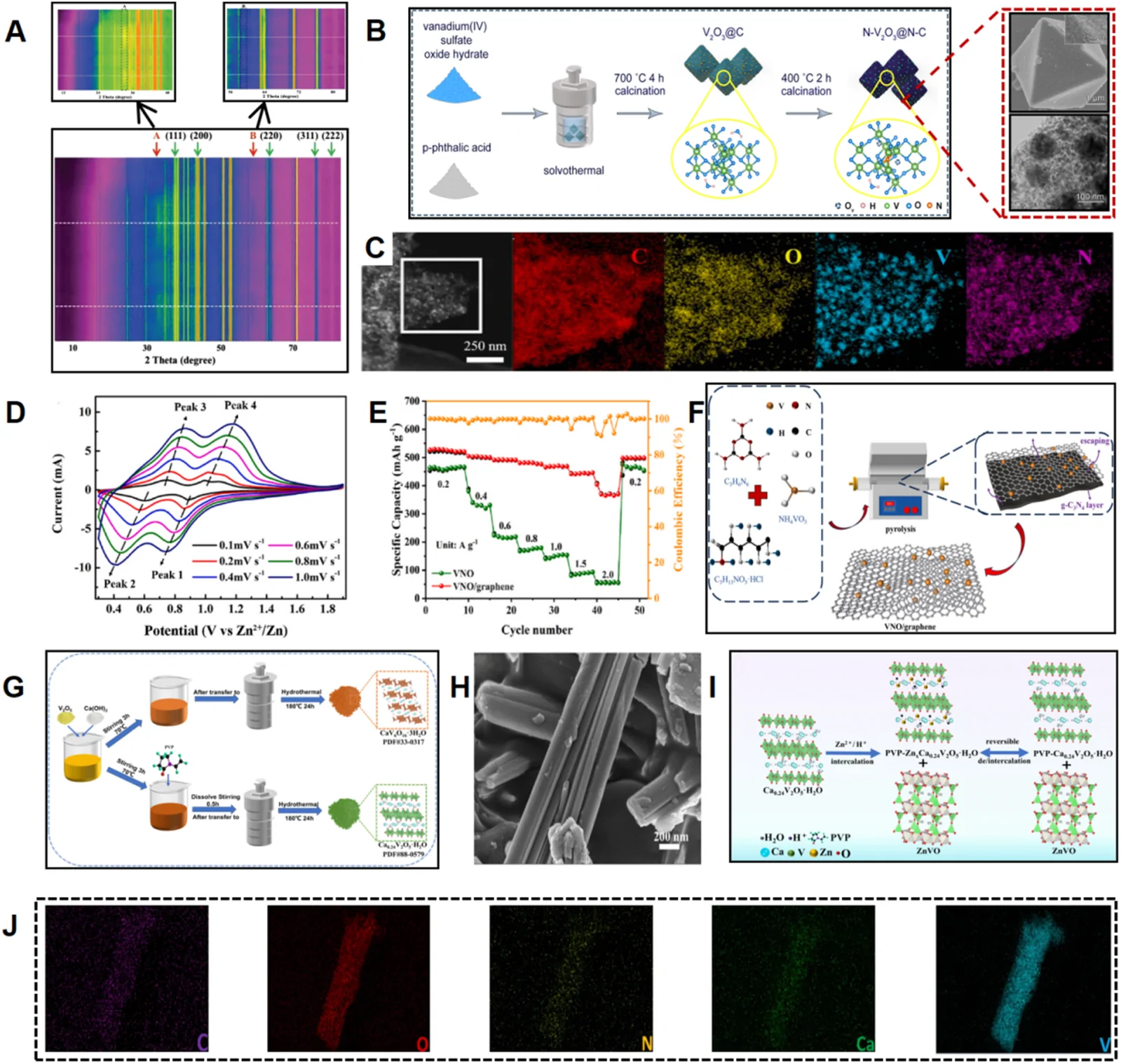
(A) 2D contour image of *operando* XRD peak intensities for VN_0.9_O_0.15_ as a function of cycling time, including high-resolution details of peak A;^75^ (B) fabrication procedure, SEM images, and TEM images of N–V_2_O_3_@N–C polyhedra;^76^ (C) elemental mapping images of VNO/graphene showing C (red), O (yellow), V (cyan), and N (purple) (for color interpretation, refer to the web version of this article); (D) CV curves of VNO/graphene electrodes at various scan rates; (E) rate performance of VNO, graphene, and VNO/graphene at different current densities; (F) schematic of the VNO/graphene preparation process.^77^ (G) Synthesis process of CaVO and PVP–CaVO; (H) SEM image of PVP–CaVO; (I) Zn^2+^ storage mechanism in PVP–CaVO; (J) SEM-EDS elemental mapping of PVP–CaVO;^78^ adapted from ref. 75 with permission from Wiley-VCH, copyright 2019; ref. 76 with permission from the American Chemical Society, copyright 2024; ref. 77 with permission from Elsevier, copyright 2025; and ref. 78 with permission from the Royal Society of Chemistry, copyright 2025.

**Fig. 5:**
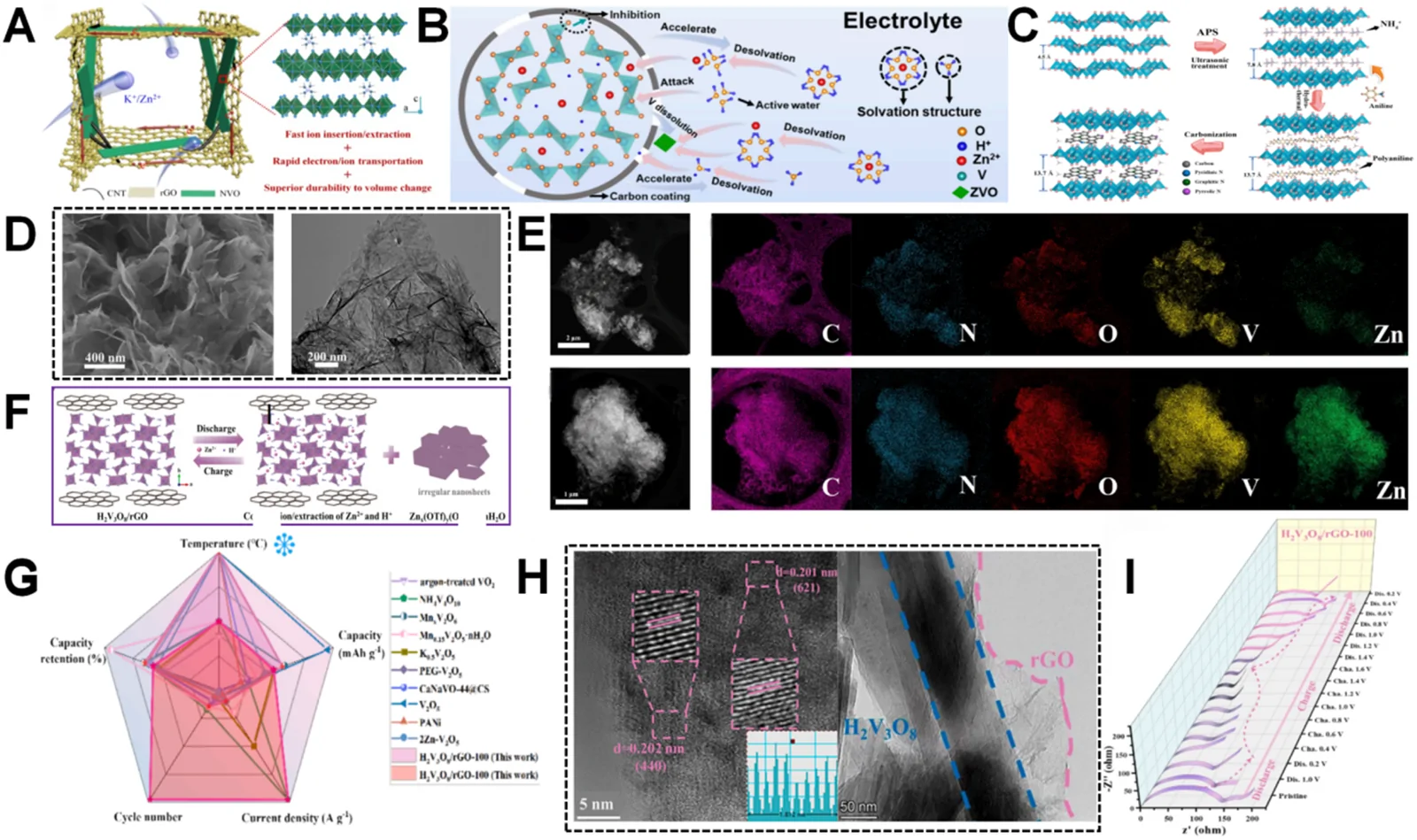
(A) 2D contour image of *operando* XRD peak intensities for VN_0.9_O_0.15_ as a function of cycling time, including high-resolution details of peak A;^75^ (B) fabrication procedure, SEM images, and TEM images of N–V_2_O_3_@N–C polyhedra;^76^; (C) CNVO synthesis; (D) SEM of CNVO; TEM of CNVO; (E) EDS mapping of CNVO charged/discharged;^68^ (F) Zn^2+^/H^+^ co-insertion in H_2_V_3_O_8_/rGO; (G) low-temp performance radar chart; (H) TEM of H_2_V_3_O_8_/rGO-100; (I) *in situ* electrochemical impedance spectroscopy (EIS) of H_2_V_3_O_8_/rGO-100;^79^ adapted from ref. 75 with permission from Wiley-VCH, copyright 2019; ref. 76 with permission from the American Chemical Society, copyright 2024; and ref. 68 and 79 with permission from Elsevier, copyright 2025.

#### 2.1.2 Surface engineering

Surface engineering, as a precise interface control strategy, enhances the electrochemical performance of vanadium-based cathode materials by constructing a nanoscale modified layer or functionalized surface structure with specific physicochemical properties on the surface of active material particles. Beyond simple conductivity enhancement, surface engineering fundamentally alters interfacial reaction pathways by regulating Zn^2+^ adsorption energetics, suppressing parasitic by-product formation, and stabilizing surface vanadium species.^75^ In particular, artificial interphases and polymer coatings act as selective ion-conducting layers that decouple bulk Zn^2+^ diffusion from surface side reactions.^80^

Huan and other researchers developed a composite material (VPMX) with a Ti_3_C_2_T_*x*_ MXene layer coated on the surface of V_2_O_5_ nanosheets, synthesized through van der Waals self-assembly, aiming to suppress the vanadium dissolution problem of the V_2_O_5_ cathode in AZIBs. Compared with traditional V_2_O_5_/carbon composites, this material has three major advantages: the MXene coating retains structural integrity, the heterointerface enhances kinetics, and the interlayer water molecules promote Zn^2+^ diffusion. The synthesis involves the dispersion of MXene in NMP and the self-assembly with V_2_O_5_ nanosheets to form an efficient coating.

Ti_3_C_2_T_*x*_ MXene is a thin-layered nanosheet with a lateral size of 0.5–1 µm and a thickness of approximately 3 nm (as measured by atomic force microscopy (AFM)), and XRD shows a typical (002) peak. In VPMX, MXene uniformly covers the surface of V_2_O_5_, forming a hetero-layered structure. Elemental mapping shows that Ti, V, and O are evenly distributed. XRD retains the V_2_O_5_ peak and shows split peaks at 7.7° and 8.9°, Raman confirms the typical vibration mode, and XPS shows an additional Ti–O peak at 459.4 eV, indicating strong interfacial interaction. The composite has a mesoporous structure with pore diameters ranging from 2 to 50 nm, a specific surface area of approximately 14 m^2^ g^−1^, a contact angle of 55°, and a conductivity of 920.5 S cm^−1^.

In a 3 M Zn(CF_3_SO_3_)_2_ electrolyte, when the mass ratio of V_2_O_5_ to MXene is 7 : 3, the capacity reaches 416.7 mAh g^−1^ at 0.3 A g^−1^ and remains at 243.6 mAh g^−1^ at 5.0 A g^−1^. At 10 A g^−1^, the capacity retention is approximately 99.5% after 5000 cycles. Galvanostatic intermittent titration technique (GITT) indicates that the diffusion coefficient of Zn^2+^ is 10^−10^ to 10^−9^ cm^2^ s^−1^, and the charge transfer resistance from EIS is 50.7 Ω.

During discharge, V_2_O_5_ forms Zn_*x*_V_2_O_5_ and then transforms into Zn_*x*_V_2_O_5_·*n*H_2_O; during charging, the process reverses. Water molecules shield the electrostatic force, lowering the activation energy and facilitating reversible insertion. The structure remains stable in the second cycle. DFT calculations show that the adsorption energy of Zn at the heterointerface is the lowest, and the differential charge density indicates charge accumulation in V_2_O_5_. Density of states (DOS) shows that the Fermi level shifts towards the conduction band, making the hybrid material metal-like and enhancing conductivity. CI-NEB calculations reveal the lowest diffusion barrier, promoting Zn^2+^ diffusion. ORCA calculations show that MXene binds to vanadyl ions (VO_2_^+^ −0.23 hartree and VO(OH)_2_^−^ −0.07 hartree), inhibiting dissolution (low V content by inductively coupled plasma (ICP) and a small pH change). ICP and immersion experiments confirm the inhibition of dissolution, and SEM shows that the structure remains intact after cycling. CV indicates that pseudocapacitance dominates (63.8% at 1.0 mV s^−1^), and *b* values of 0.78–0.85 confirm non-diffusion control.^81^

Weicai *et al.* combined the HCl/LiF and hydrothermal treatments to synthesize Ta_4_AlC_3_. The four layers of Ta atoms are densely packed into an octahedral structure, with three layers of C atoms embedded within, forming a Ta_4_C_3_ monolayer structure. The material has a compact axial layered structure, rough surface, and micrometer-sized grains (Fig. 6A and B).^82^

**Fig. 6:**
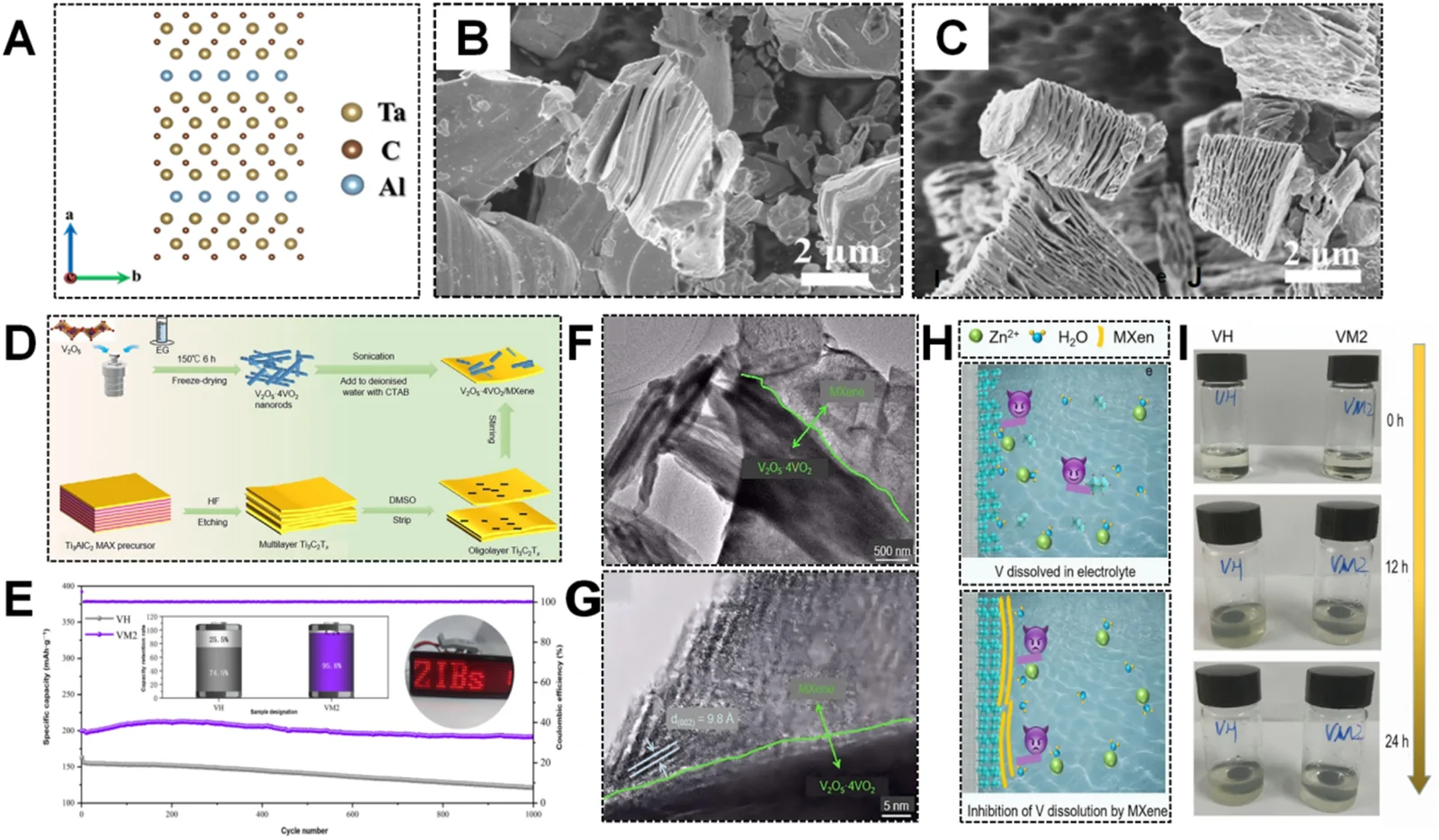
(A) Schematic atomic structure of Ta_4_AlC_3_. (B) SEM of parent Ta_4_AlC_3_. (C) Accordion-like Ta_4_C_3_.^82^ (D) Synthesis schematic of V_2_O_5_·4VO_2_/MXene composites. (E) Long-cycle performance at 10 A g^−1^ for the two electrodes; (F) TEM and (G) HRTEM of V_2_O_5_·4VO_2_/MXene. (H) Schematic of V dissolution in electrolyte for VH and VM_2_. (I) Optical images of VH and VM_2_ in 3 M Zn(CF_3_SO_3_)_2_ at varying soak times.^83^ Adapted from ref. 82 with permission from the American Chemical Society, copyright 2023; and ref. 83 with permission from Springer Nature, copyright 2025.

Sun and co-workers successfully prepared VOH@PEDOT composite materials by coating poly(3,4-ethylenedioxythiophene) (PEDOT) on the surface of water-intercalated V_2_O_5_ (*i.e.*, V_2_O_5_·*n*H_2_O, abbreviated as VOH). The morphology of this material consists of short and disordered nanoribbons (as shown in FE-SEM images), with a nanoribbon diameter of approximately 180 nm. TEM images reveal a clear interface between VOH and PEDOT, with an amorphous PEDOT layer unevenly coating the surface of VOH and having thickness ranging from several nanometers to tens of nanometers. HRTEM images further show that the lattice fringe spacing on the surface of the material is 0.197 nm.

The Zn//VOH@PEDOT battery was assembled using VOH@PEDOT as the cathode material, achieving a specific capacity of 432 mAh g^−1^ at 0.1 A g^−1^. After 35 cycles of increasing the current density from 0.1 A g^−1^ to 2 A g^−1^ and then back to 0.1 A g^−1^, the capacity remained at 377 mAh g^−1^. After 100 cycles at 0.1 A g^−1^, the capacity remained at 323 mAh g^−1^. Additionally, this battery demonstrated a high energy density of 255 Wh kg^−1^ at a power density of 245 W kg^−1^.

The CV curves show similar redox peaks, indicating good reversibility. The ion diffusion behavior according to the equation *i* = *av*^*b*^ showed that the *b* value was close to 0.5, confirming that diffusion control was dominant. This indicates that the PEDOT coating enhanced conductivity without hindering the ion transport of VOH, thereby improving the overall electrochemical performance. During the first discharge to 0.2 V, an intermediate phase Zn_3_(OH)_2_V_2_O_7_·2H_2_O was formed and the interlayer spacing increased to 13.5 Å, which was favorable for Zn^2+^ insertion. During subsequent charge–discharge cycles, the diffraction peak of this phase repeatedly appeared and disappeared, confirming the highly reversible intercalation and deintercalation of Zn^2+^ in the interlayer. DFT calculations further confirmed that the PEDOT coating could improve the conductivity and structural stability of VOH.^84^

Song and his team synthesized 1,5-naphthalenediamine-composited VO_2_ (VO@NDA). The VO_2_ inside provides the sites for Zn^2+^ storage, and the NDA outside can act as the electron acceptor and prevent the VO_2_ from dissolving. The CV curves indicate that, although the discharge platform of the VO@NDA electrode has decreased by 0.06 V, it's more stable during long-term cycling (Fig. 7A).^85^ Duan *et al.* designed PEDOT–NH_4_V_3_O_8_ as a cathode material. PEDOT–NVO shows a connected nanobelt structure, and the lattice spacing is about 10.8 Å, which is bigger than NVO's. What's more, according to (STEM)-energy-dispersive X-ray spectroscopy (EDS), they found that the N, V, and O elements are uniformly distributed throughout the material and the low content of the element N is caused by the PEDOT occupying the original sites of NH_4_^+^ or forming new vacancies (Fig. 7B–D). For its electrochemical mechanism, they used XRD, FTIR, and Raman analyses and found that the discharge reaction occuring at the cathode is PEDOT/NH_4_V_3_O_8_ + *x*Zn^2+^ + 2X^e−^ → PEDOT/Zn_*x*_NH_4_V_3_O_8_ at the initial stage (Fig. 7E). In the electrochemical test, the cathode material shows a good specific capacity; it can achieve 356.8 mAh g^−1^ at 0.05 A g^−1^. What's more, when the current density increased to 10 A g^−1^, it still could achieve 163.4 mAh g^−1^ (Fig. 7F).^86^ Zhang *et al.* synthesized the V_2_O_5_–PPy nanosheet *via* polymerization at room temperature. At the discharge state, Zn, V, O, and N are distributed uniformly on the surface. In contrast, the signal of Zn becomes weak at the charge state, indicating that Zn has been extracted (Fig. 7G). According to the XPS analysis, they observed the components V^4+^ and V^5+^; at the fully charged state, V^3+^ and the majority of V^4+^ are oxidized back to V^5+^. The curve is similar to that in the initial state. This result indicates a reversible redox reaction. What's more, the O 1s XPS spectrum shows two peaks, indicating that the two peaks respectively belong to VO_*x*_ and VOH (Fig. 7H).^87^

**Fig. 7:**
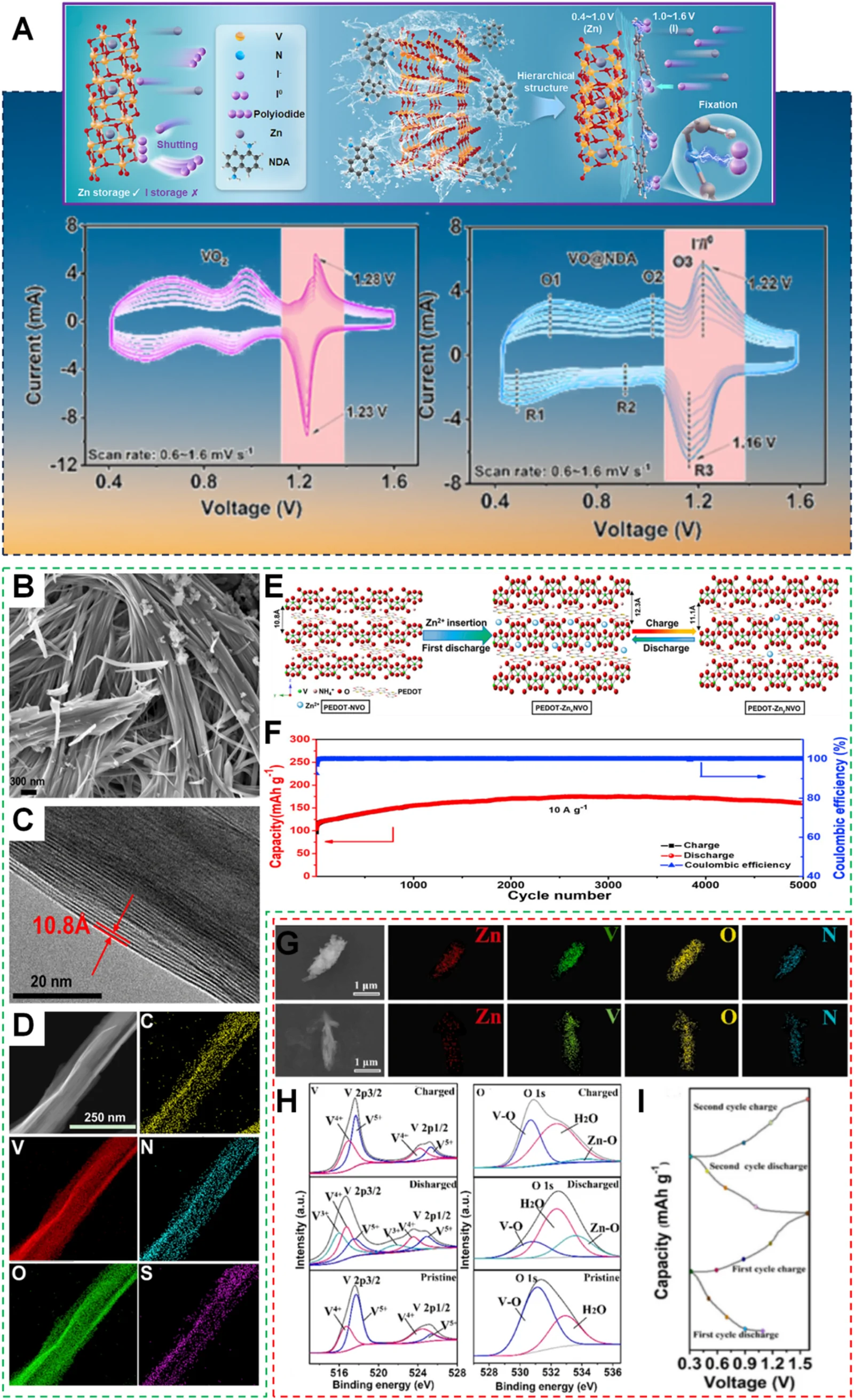
(A) Storage mechanism for the VO@NDA cathode; CV curves of V_2_O_5_; CV curves of VO@NDA;^85^ (B) SEM of PEDOT–NVO; (C) HRTEM of PEDOT–NVO; (D) elemental mapping of PEDOT–NVO; (E) schematic of Zn^2+^ intercalation/extraction in PEDOT–NVO during cycling; (F) long-term cycling of Zn//PEDOT–NVO at 10 A g^−1^ (full composite basis; loading ∼2 mg cm^−2^);^86^ (G) elemental mapping of V_2_O_5_–ppy charged/discharged; (H) XPS (V 2p and O 1s) of V_2_O_5_–ppy; (I) typical discharge/charge curves in the first two cycles at 0.1 A g^−1^.^87^ Adapted from ref. 85 with permission from the Royal Society of Chemistry, copyright 2024; and ref. 86 and 87 with permission from Elsevier, copyright 2020 and 2022, respectively.

Li *et al.* successfully synthesized a novel cathode material for AZIBs, namely yolk–shell V_2_O_3_/VN heterojunction microspheres (VON@SiO_2_), by hydrothermal preparation of VO_2_ precursors followed by annealing and coating with SiO_2_. This material retains the porous yolk–shell microsphere morphology but is slightly smaller in size (∼1 µm) compared to the VO_2_ precursors (∼1.3 µm). HRTEM analysis revealed that the (202) and (006) interplanar spacings of V_2_O_3_ are 0.205 nm and 0.233 nm, respectively, while that of VN's (111) plane is 0.238 nm, and SiO_2_'s (203) plane is 0.145 nm. This indicates that VON@SiO_2_ is a polycrystalline structure, with detectable (214) plane of V_2_O_3_, (311) plane of VN, and (221) and (311) planes of SiO_2_. The size of the aperture distribution of this material is approximately 2–10 nm, and its specific surface area is 38.714 m^2^ g^−1^, significantly higher than that of VO_2_ (11.082 m^2^ g^−1^) and VON (14.870 m^2^ g^−1^). The larger specific surface area exposes more active sites, promoting a larger electrochemical interface and enhancing electrolyte penetration. The CV curves show that the oxidation–reduction peaks of this material are narrower than those of VON and VO_2_, indicating lower polarization and better reversibility. At 0.5 A g^−1^, the highest discharge capacity is 483.5 mAh g^−1^, and the capacity retention rate after 50 cycles is 91%. At a high current density of 10 A g^−1^, the highest specific capacity is 294 mAh g^−1^, and the capacity retention rate after 1000 cycles is still 94%. Moreover, the SEM images after 500 cycles show that the material retains its initial microsphere structure, demonstrating good microstructural stability. *In situ* XRD analysis reveals that during discharge, the (104) plane of V_2_O_3_ and the (111) plane of VN shift to lower angles. At the same time, the intensity of VN's (311) plane is weakened due to the successful intercalation of Zn^2+^. During charging, the crystal plane positions and intensities return to their initial states. Additionally, a partial phase transformation occurs in the material during discharge, forming Zn_3_(OH)_2_V_2_O_7_·2H_2_O, which weakens or disappears after charging, confirming its participation in the zinc-ion storage process.^88^

Guo *et al.* deposited an ultrathin HfO_2_ layer on the surface of Zn_3_(OH)_2_V_2_O_7_·2H_2_O (referred to as ZVO) *via* atomic layer deposition (ALD) technology, serving as an artificial solid electrolyte interface (SEI) to suppress cathode dissolution and surface side reactions in AZIBs. XPS analysis indicated that the valence state of V (V^5+^) was not affected by the HfO_2_ coating. High-resolution TEM images revealed that the ZVO surface was uniformly covered with a HfO_2_ coating approximately 5 nm thick, while the (022) crystal plane spacing of ZVO was 0.21 nm. These characterization results confirmed that the HfO_2_ layer had been successfully deposited on the surface of the ZVO self-supporting electrode film.

The battery assembled with this material as the cathode, zinc foil as the anode, and 1 M ZnSO_4_ as the electrolyte exhibited a higher *b* value than the uncoated ZVO electrode in electrochemical performance tests, indicating enhanced reaction kinetics. In the rate performance test, the current density was gradually increased from 100 mA g^−1^ to 3000 mA g^−1^ and then decreased back to 500 mA g^−1^. The reversible discharge capacities of the HfO_2_-coated ZVO cathode at 100, 300, 500, 800, 1000, and 3000 mA g^−1^ were 215, 190, 165, 120, 106, and 88 mAh g^−1^, respectively. When the current density returned to 500 mA g^−1^, the recoverable average discharge capacity was 162 mAh g^−1^.

*Ex situ* XRD and electrochemical impedance spectroscopy (EIS) experiments revealed the zinc-ion storage mechanism: during discharge, H_2_O and Zn^2+^ co-insert into the interlayer of the ZVO electrode, but subsequent charging leads to the de-intercalation of Zn^2+^ and the restoration of the initial structure. Meanwhile, as the voltage decreases, the insulating by-product Zn_4_SO_4_(OH)_6_·*x*H_2_O forms, which increases the internal resistance; during charging, this by-product gradually disappears. The HfO_2_ coating significantly slows down the formation of by-products and the cathode dissolution process, thereby enhancing overall performance.^89^

#### 2.1.3 Defect engineering

Defect engineering is a key strategy to enhance the performance of vanadium-based oxide cathode materials. By introducing oxygen vacancies, cation vacancies or hetero-doping defects, it can improve electronic conductivity, ion diffusion kinetics, and structural stability, thereby alleviating capacity fading and dissolution issues. Defects influence electrochemical behavior through two dominant mechanisms: (i) electronic structure modulation, where oxygen or cation vacancies introduce mid-gap states and increase electronic conductivity^90^ and (ii) ionic regulation, where reduced local electrostatic potential lowers Zn^2+^ adsorption energy and migration barriers. However, excessive defect concentrations may trigger irreversible phase evolution, highlighting the need for defect-density optimization rather than maximization. Although a universal threshold is difficult to define because defect tolerance depends on the host structure, available reports suggest that moderate oxygen-vacancy levels, often reflected by a partial V^4+^ fraction of *ca.* 20–35% in oxygen-deficient vanadium oxides, can enhance conductivity and Zn^2+^ diffusion, whereas excessive vacancy generation or strong etching may collapse the layered framework. For cation-deficient V_2_O_3_, vanadium vacancy concentrations around 5–6% have been reported to improve Zn^2+^ storage without severe structural degradation. Therefore, defect engineering should be treated as a material-specific optimization window rather than a monotonic enhancement strategy.^91,92^

Chen *et al.* synthesized vanadium oxide nanofibers (VCN) with physical and chemical defects *via* electrospinning. SEM images revealed that VCN was composed of slender fibers with a diameter of approximately 340 nm. These slender fibers can effectively inhibit the self-aggregation of active vanadium oxide and maintain stability. The fibers contained numerous pores with diameters greater than 15 nm. HRTEM confirmed that VCN was composed of VO (110) nanocrystals with a lattice spacing of 0.34 nm and was modified by a wrinkled carbon shell, enhancing conductivity, ion diffusion, and the insertion/extraction of Zn^2+^. XRD patterns indicated that the surface of VCN was covered with amorphous carbon, and the *I*_D_/*I*_G_ ratio in Raman spectra was 1.47, indicating disordered carbon bonding and partial graphitization. These chemical defects improve the reversible insertion/extraction of Zn^2+^. N_2_ adsorption–desorption experiments showed that the specific surface area of VCN was 15.43 m^2^ g^−1^, promoting the deintercalation of Zn^2+^. TGA analysis confirmed the stoichiometric formula of V_2_O_4.59_, and oxygen vacancies provided more active sites.^93^

In electrochemical performance tests, the CV curve showed that the first cycle cathodic peak (0.88 V) split into 0.87 V and 0.93 V, attributed to the multiple insertions of zinc ions induced by defects. The rate performance was excellent. The capacities at 1, 2, 5, and 10 A g^−1^ respectively, were 246, 210, 137, and 73 mAh g^−1^ and returned to 238 mAh g^−1^ at 1 A g^−1^, indicating high reversibility and fast kinetics.

The mechanism analysis was conducted by calculating the Zn^2+^ diffusion coefficient 
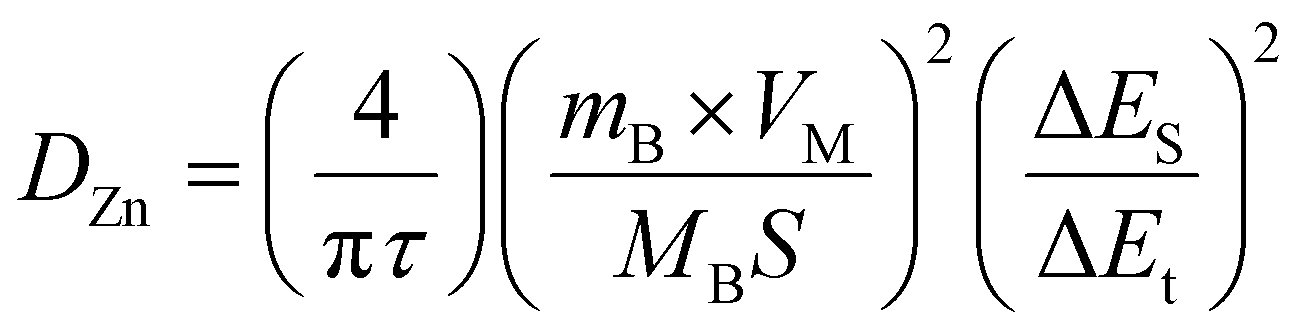
 through GITT, obtaining *D*_Zn_ ≈ 10^−9^ to 10^−10^ cm^2^ s^−1^. This method can help to quantify the enhancement of ion transport by defects. According to the CV kinetic equation *i* = *av^b^*, the *b* values are in the range of 0.71–0.78, indicating a mixed control process. The capacitive contribution is 65.8% (at 1 mV s^−1^), demonstrating that defects enhanced surface reactions. Additionally, because –OH groups bind to water molecules, the number of –OH in the lattice decreases, leading to reduced effective charges, decreasing electrostatic interactions, and promoting Zn^2+^ diffusion. *In situ* Raman and *ex situ* XPS verified the Zn^2+^/H^+^ co-intercalation mechanism: V_2_O_4.59_ + *x*Zn^2+^ + *n*H_2_O + 2*x*e^−^ ↔ Zn_*x*_V_2_O_4.59_·*n*H_2_O, which elucidated the defect-driven reversible phase transition.^93^

Gong *et al.* synthesized an organometallic (ferrocene) pre-intercalated vanadium oxide cathode (Fec/Od-VO) with a defective structure through a redox intercalation method for high-rate ZIBs. This material expands the V–O layer spacing and introduces oxygen vacancies, making Zn^2+^ diffusion and adsorption easier. The XRD pattern shows a (001) peak shift, with the interlayer spacing increasing to 5.62 Å. SEM/TEM reveals a nanoribbon structure, with a width of 50–100 nm. HRTEM shows a 0.36 nm lattice spacing, and disordered regions indicate oxygen vacancies. XPS confirms a mixed V^4+^/V^5+^ state, with oxygen vacancies of ∼28.5%. EPR verifies the presence of vacancies. The specific surface area of Fec/Od-VO is 32.6 m^2^ g^−1^, and the mesoporous structure is beneficial for ion transport.^94^

In electrochemical performance tests, Fec/Od-VO has a capacity of 411.7 mAh g^−1^ at 0.5 A g^−1^ and about 200 mAh g^−1^ at 20 A g^−1^. It exhibits excellent rate performance with a capacity recovery of >95%. For cycling stability, at 20 A g^−1^, it retains 86.5% after 3000 cycles.

The team used the same method, GITT calculation, and CV equation, as Chen *et al.* They obtained *D*_Zn_ ≈ 10^−8^ to 10^−10^ cm^2^ s^−1^, indicating that oxygen vacancies lower the energy barrier. In the CV equation, the values of *b* are 0.75–0.85, indicating a pseudocapacitive contribution of ∼70% (2 mV s^−1^), enhancing surface reactions. Additionally, the team used DFT to show that intercalation reduces the adsorption energy (−1.2 eV) and optimizes the electron density. *In situ* XRD/XPS verify Zn^2+^/H^+^ co-intercalation: discharge forms Zn_*x*_V_2_O_5_·*n*H_2_O and charging reverses it, with vacancies inhibiting dissolution.^94^

Guo *et al.* proposed an economically scalable method to prepare oxygen-deficient V_2_O_5−*x*_·*n*H_2_O (VOd) with tunable vacancy concentration for flexible ZIBs. The defects provide additional Zn^2+^ storage sites, reduce the intercalation electrostatic barrier, and enhance capacity and stability. The XRD pattern shows an increased interlayer spacing to 13.2 Å (4.3 Å for pure V_2_O_5_), XPS confirms an increased V^4+^ proportion (∼32%), and EPR verifies enhanced vacancy signals. SEM/TEM reveal a nanoribbon morphology (width ∼100 nm), a specific surface area of ∼45 m^2^ g^−1^, and a mesoporous structure that promotes electrolyte penetration.^95^

In electrochemical performance tests, VOd has a capacity of 415 mAh g^−1^ at 0.2 A g^−1^ and an energy density of 294 Wh kg^−1^; at 20 A g^−1^, it has a capacity of 236 mAh g^−1^ with a 0.004%/cycle decay over 5000 cycles. The flexible quasi-solid-state battery has a capacity of 200 mAh g^−1^ at 2 A g^−1^ and remains stable after repeated bending.

Mechanism analysis through GITT yields *D*_Zn_ ≈ 10^−9^ to 10^−11^ cm^2^ s^−1^, with vacancies reducing the diffusion energy barrier. CV shows *b* values of 0.8–0.9, with a pseudocapacitance contribution of ∼60%, enhancing surface reactions. DFT shows that vacancies reduce the Zn^2+^ adsorption energy (−0.95 eV), and *in situ* XRD verifies reversible phase transformation: discharge forms Zn_*x*_V_2_O_5−*x*_·*n*H_2_O and charging restores it, with vacancies inhibiting dissolution.^95^

Zhu *et al.* introduced vanadium vacancies (up to 5.7%) in the V_2_O_3_ lattice through defect engineering for the cathode of long-cycling AZIBs. These vacancies improved Zn^2+^ storage and stability. Rietveld refinement of neutron/X-ray powder diffraction confirmed the monoclinic phase structure, with lattice contraction (*a*/*b*/*c* parameters decreased by ∼0.5%) due to the vacancies; positron annihilation spectroscopy indicated a defect concentration of ∼5.7%; synchrotron XAS/XPS verified the dominance of V^3+^, and the O K-edge spectrum showed changes in electronic states induced by the vacancies. SEM/TEM revealed a nanoparticle morphology (∼50 nm) with a specific surface area of ∼28 m^2^ g^−1^, promoting ion diffusion.^92^

In electrochemical performance tests, the vanadium-deficient V_2_O_3_ exhibited a capacity of 420 mAh g^−1^ at 0.1 A g^−1^ and retained 81% of its capacity after 30 000 cycles at 5 A g^−1^. Rate performance was as follows: ∼250 mAh g^−1^ at 10 A g^−1^, with an energy density of ∼300 Wh kg^−1^. Mechanism analysis through DFT calculations showed that vanadium vacancies reduced the Zn^2+^ adsorption energy (−1.5 eV *vs.* −0.9 eV for defect-free V_2_O_3_), decreased electrostatic interactions, and promoted diffusion (*D*_Zn_ ∼ 10^−9^ cm^2^ s^−1^). *In situ* XRD verified reversible Zn^2+^ insertion: the formation of the Zn_*x*_V_2_O_3_ phase during discharge and its recovery during charging; vacancies suppressed dissolution, ensuring stability.^92^ In electrochemical tests, HEC-intercalated vanadium oxide (VO-i-HEC) achieved a capacity of 499.88 mAh g^−1^ at 0.1 A g^−1^ and exhibited a capacity decay of only 0.004% after 2000 cycles at 10 A g^−1^.^96^

Tang and his team synthesized NH_4_V_3_O_8_ at different temperatures (325, 350, and 375 °C) as shown in Fig. 8B. Among them, due to nanoparticles attaching on the surface, 350NVO is the best sample. What's more, the team put forward the storage mechanism of 350NVO, as shown in Fig. 8A, according to the analysis of non-*in situ* characterization. The electrochemical performance of the material is also remarkable. Fig. 8C shows the capacity of 350NVO at different current densities. With the current density increasing from 0.1 A g^−1^ to 2.0 A g^−1^ and then returning to 0.1 A g^−1^, 350NVO can achieve a high capacity of 444.0 mAh g^−1^, which is about 99% of the initial capacity.^90^ Yang's team used a method combining hydrothermal technology and *in situ* electrochemical oxidation, in which the material was charged to 1.7 V to stimulate the phase transition of VO, as shown in Fig. 8D. They made use of the selected-area electron diffraction (SAED) and HAADF-STEM to reveal the morphology and microstructure of VO, with the elements V and O uniformly distributed on the nanorods (Fig. 8E).^91^ Cho *et al.* designed amorphous oxygen-deficient V_2_O_5−*x*_ through the RF magnetron sputtering process, as shown in Fig. 8F, which had a capacity of 57.2 mAh g^−1^ at 20C and reached 105.2 mAh g^−1^ after 1000 cycles at 5C.^97^

**Fig. 8:**
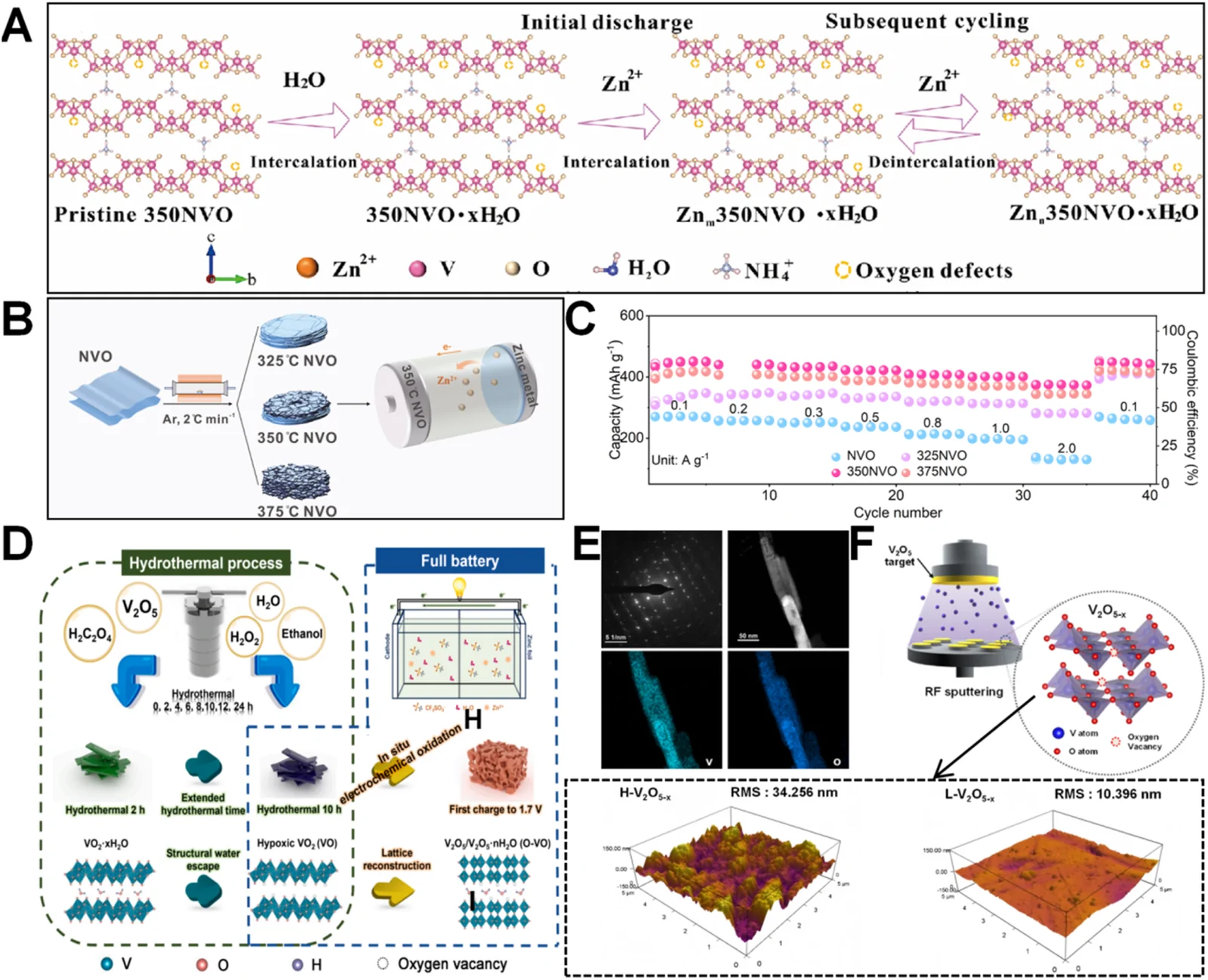
(A) The schematic diagram of the zinc-ion storage mechanism of the proposed 350NVO. (B) Fabrication processes of cation-modulated NVO nanosheets. (C) Rate performances of the four NVO electrodes.^90^ (D) Formation mechanism of VO and its *in situ* electrochemical oxidation. (E) SAED and HAADF-STEM elemental mapping images;^91^ (F) synthesis of oxygen-deficient V_2_O_5−*x*_ by sputtering; AFM images of H-V_2_O_5−*x*_ and L-V_2_O_5−*x*_ films prepared *via* RF magnetron sputtering at varying Ar pressures.^97^ Adapted from ref. 90 and 97 with permission from the American Chemical Society, copyright 2024 and 2023, respectively; and ref. 91 with permission from Elsevier, copyright 2024.

#### 2.1.4 Heterojunctions

Wu *et al.* prepared sea urchin-like V_2_O_3_–VN nanoheterojunction composite materials as the cathode for AZIBs. The Scherrer formula calculation showed that the grain sizes of V_2_O_3_ and VN in V_2_O_3_–VN were 297.1 Å and 125.3 Å, respectively, which were significantly smaller than those of commercial V_2_O_3_ (302.3 Å) and VN (180 Å), attributed to the heterointerface interaction. This reduction in grain size increased the electrode–electrolyte contact area and promoted the reaction. EDS mapping confirmed the uniform distribution of V, O, and N elements, indicating a tight coupling. SEM showed a ribbon-like nanostructure, and HRTEM revealed the diffraction rings of V_2_O_3_ (113)/(104) and VN (220) crystal planes, confirming the formation of the heterojunction. XPS showed a decrease in the V electron density, which was attributed to the strong coupling between V_2_O_3_ and VN. Heterojunction design introduces built-in electric fields at phase boundaries, which promote directional electron transport and redistribute local charge density.^94^ This interfacial polarization effect effectively lowers Zn^2+^ diffusion barriers and enhances redox kinetics, making heterostructures particularly advantageous for high-rate AZIB applications.^95^

In the electrochemical test, this electrode can achieve a capacity of 532.6 mAh g^−1^ at a current density of 0.1 A g^−1^. And at a higher current density of 10 A g^−1^, it can reach 116.7 mAh g^−1^. When it returns to 0.3 A g^−1^, its capacity can also be restored to 547.9 mAh g^−1^. This proves its reliable reversibility. It also showed good cycling stability, retaining 90.8% of its capacity at 5 A g^−1^. EIS analysis revealed that *R*_ct_ was 201 Ω, which was lower than that of VN (572.6 Ω) and V_2_O_3_ (785.9 Ω), indicating that the heterojunction interface accelerated electron transfer. The CV curve exhibited two pairs of redox peaks (0.78/0.54 V and 1.23/0.92 V), and the 8th scan was stable, reflecting the initial activation process. The GITT calculation showed that the diffusion coefficient of Zn^2+^ was higher than that of the single component, confirming that the heterojunction provided a fast channel and improved kinetics.^98^

Zhang *et al.* compared MVO + V_2_C with MVO and found that MVO + V_2_C, which was synthesized *via* the nanofilm electrostatic self-assembly method (Fig. 9B and C), can stimulate the transport of the electrolyte, benefiting from rich nanochannels in heterogeneous structures (Fig. 9A). Electrostatic interactions between intercalated Zn^2+^ and the host framework can hinder further ion insertion. In the MVO + V_2_C heterostructure, the MVO/V_2_C heterointerface facilitates charge-carrier transport and enhances the reaction kinetics of the composite cathode (Fig. 9D). The TEM and elemental mapping show that the distribution of V, Mn, O, and C is uniform, as shown in Fig. 9E. The team used DFT calculations to find that the diffusion barrier of MVO + V_2_C is lower than that of MVO, indicating that the heterogeneous structure can promote the diffusion of Zn^2+^ (Fig. 9F).^99^

**Fig. 9:**
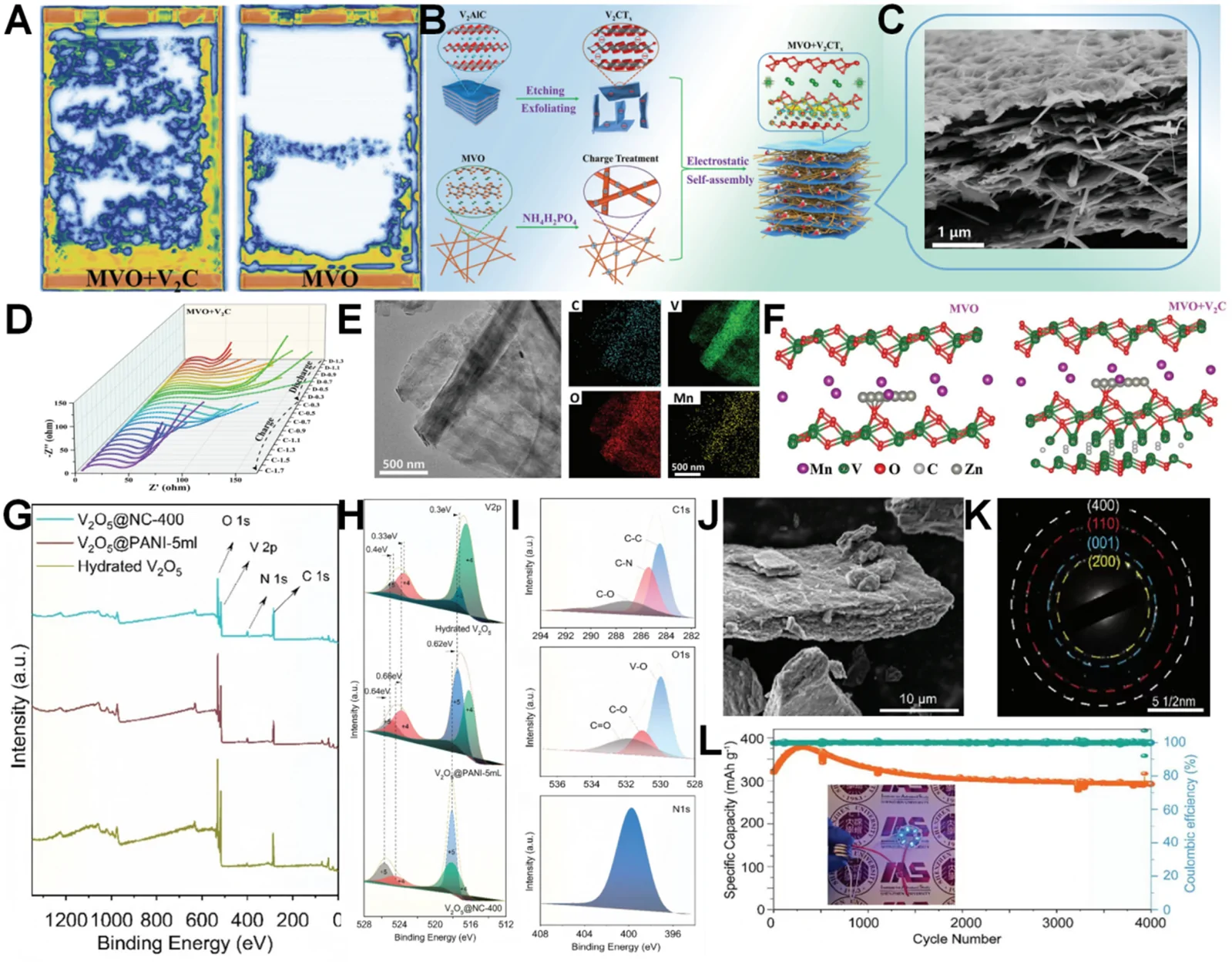
(A) Ultrasonic images of MVO + V_2_C and MVO pouch cells. (B) Self-assembly schematic of MVO and V_2_CT_*x*_. (C) Cross-sectional SEM of MVO + V_2_C. (D) *In situ* Nyquist plots of MVO + V_2_C under charge/discharge. (E) TEM & elemental mapping. (F) Zn^2+^ diffusion pathway in MVO + V_2_C.^99^ (G) Full XPS of the samples. (H & I) High-resolution V 2p, C 1s, O 1s, and N 1s XPS spectra of V_2_O_5_@NC-400. (J) Layered SEM of V_2_O_5_@NC-400. (K) Dark-field TEM of the V_2_O_5_@NC-400 area. (L) Long-cycle performance & CE of V_2_O_5_@NC-400 at 5 A g^−1^.^100^ Adapted from ref. 99 and 100 with permission from Wiley-VCH, copyright 2024.

Najeeb *et al.* synthesized V_2_O_5_@NC-400. The XPS spectrum shows a peak at 399.1 eV, as shown in Fig. 9G–I, which corresponds to N 1s, indicating that PANI and N-doped C were successfully combined into the V_2_O_5_ layers. The material exhibits a typical layered structure, as shown in Fig. 9J. What's more, according to the high-resolution image of the section (Fig. 9K), there were two types of layers, one is V_2_O_5_, with *d* = 12 Å and the other one is NC, with *d* = 4.2 Å. The total interlayer spacing is 16.2 Å. As shown in Fig. 9L, the material can achieve 268 mAh g^−1^ after 4000 cycles at 5.0 A g^−1^. The capacity retention rate is as high as 92%, and the CE value is close to 100%.^100^

The results revealed that the value for V_2_O_3_–VN was −2.44 eV, significantly lower than that of V_2_O_3_ (−0.67 eV) and VN (−0.41 eV), which enhanced the adsorption and intercalation/deintercalation of Zn^2+^. DOS analysis indicated that the heterojunction increased the conductivity, promoting electron migration and redox kinetics. *Ex situ* XRD revealed the formation of a new phase, Zn_3_(OH)_2_V_2_O_7_·2H_2_O, during discharge, confirming the co-intercalation mechanism of Zn^2+^/H^+^.^101^

Liu *et al.* designed an oxygen-deficient vanadium oxide nanocomposite material with a thin amorphous carbon coating on the outer layer (Od-VO@C). Its precursor, VNO@C, has a pea-like heterostructure, which is conducive to the intercalation and deintercalation of Zn^2+^. After annealing, the carbon shell is rich in submicropores, providing a Zn^2+^ transport channel and shortening the migration path. The BET test shows that the specific surface area of the composite material reaches 246 m^2^ g^−1^, which is attributed to its nanoporous structure. The group used VNO@C as the initial cathode. The cathode was charged at a current density of 0.1 A g^−1^ to 2.0 V and was activated to form Od-VO@C by the *in situ* anodization method. After activation, the cathode structure changes from dense to loose. This enhances the adaptability of the structure and its electrochemical activity.

In the electrochemical performance test, the CV curve (1 mV s^−1^) shows two pairs of redox peaks, corresponding to the V^4+^/V^3+^ and V^5+^/V^4+^ transitions. CV confirms the multi-step mechanism of Zn^2+^ intercalation/deintercalation. The subsequent curves are highly overlapping, indicating high stability and reversibility. The electrode has a capacity of 700 mAh g^−1^ at 0.1 A g^−1^ and still retains 250 mAh g^−1^ at 5 A g^−1^. Mechanism analysis reveals that Od-VO@C is rich in oxygen vacancies, which can relieve irreversible reactions and prevent highly polarized Zn^2+^ from being captured by oxygen atoms. The heterojunction and defects working together can ensure efficient Zn^2+^ storage to enhance electronic conductivity and ionic kinetics.^102^

The remaining heterojunction materials include MOF-derived C@VO_2_@V_2_O_5_,^103^ oxygen-deficient V_6_O_13−*x*_/rGO,^104^ and NC@VN/MnO.^105^ The above three types of materials can enhance the storage rate of zinc ions through the synergistic effect of the interface. C@VO_2_@V_2_O_5_ has a mesoporous nanostructure. Its specific surface area is about 150 m^2^ g^−1^ and pore size is 5–10 nm. V_6_O_13−*x*_/rGO is a 2D layered heterointerface (oxygen vacancies ∼ 25%), and NC@VN/MnO is composed of VN quantum dots/MnO nanoparticles encapsulated in a N-doped carbon shell, with a particle size of about 10 nm. These structures provide abundant active sites and short diffusion paths. In electrochemical tests, C@VO_2_@V_2_O_5_ exhibits a capacity of 376 mAh g^−1^ at 0.05 A g^−1^ and retains 90.3% after 2000 cycles at 5 A g^−1^; V_6_O_13−*x*_/rGO shows a capacity of 424.5 mAh g^−1^ at 0.1 A g^−1^ and retains 96% after 5800 cycles at 10 A g^−1^; NC@VN/MnO has a capacity of 108.3 mAh g^−1^ after 12 000 cycles at 10 A g^−1^, demonstrating excellent rate and long-cycle performance. Mechanism analysis indicates that the heterointerface builds an internal electric field to reduce the Zn^2+^ adsorption energy (DFT calculations: −1.2 eV), and the oxygen vacancies/quantum dots synergistically accelerate ion/electron transport (*D*_Zn_ ∼ 10^−9^ cm^2^ s^−1^) and suppress dissolution, ensuring reversible Zn^2+^/H^+^ co-intercalation.

### 2.2 Other vanadium-based compounds

From a mechanistic perspective, the electrochemical behavior of vanadium-based cathodes in AZIBs is governed by the interaction between Zn^2+^ electrostatics, multivalent vanadium redox chemistry, and host lattice flexibility.^64^ Unlike monovalent ion batteries, the strong coulombic interaction of divalent Zn^2+^ often leads to sluggish diffusion, severe lattice distortion, and parasitic side reactions.^65^ Therefore, effective cathode design must simultaneously regulate Zn^2+^ solvation/desolvation, lower migration barriers, and stabilize reversible V^2+^/V^5+^ redox couples while suppressing vanadium dissolution and irreversible phase transitions. Importantly, these strategies are not independent but mechanistically interconnected: interlayer species primarily reduce Zn^2+^ migration barriers through electrostatic shielding, defects modulate the electronic structure and active site density, while heterostructures create internal electric fields and fast electron pathways.^49,66^ Understanding how these modifications synergistically influence Zn^2+^/H^+^ co-intercalation and vanadium redox reversibility is critical for rational material optimization.

#### 2.2.1 Vanadium phosphates

Compared with vanadium oxides, vanadium phosphates have received relatively limited attention as cathode materials for aqueous zinc-ion batteries (AZIBs).^17^ Nevertheless, their robust polyanionic frameworks, tunable interlayer chemistry, and relatively high structural stability make them a promising yet underexplored class.^20,21^ Existing studies^24,27^ primarily concentrate on VOPO_4_-derived materials, aiming to overcome intrinsic drawbacks, such as sluggish Zn^2+^ diffusion, limited electronic conductivity, and structural degradation during cycling.

A widely adopted strategy involves interlayer engineering through organic or inorganic pre-insertion, which seeks to expand the interlayer spacing, regulate interfacial chemistry, and modulate Zn^2+^ storage behavior. For instance, the esterification-driven grafting of small organic molecules onto VOPO_4_ produces a zinc-philic, hydrophobic organic-layered vanadyl phosphate (VOPO_4_–HBSM, VPS).^25^ This modification effectively tailors the inner Helmholtz plane, mitigating free-water-induced erosion while altering the Zn^2+^ storage mechanism. As a result, VPS exhibits a markedly increased specific surface area (∼120 m^2^ g^−1^), providing abundant active sites and enabling stable capacities of 145 mAh g^−1^ at 0.2 A g^−1^ and 100.6 mAh g^−1^ after 1000 cycles at 3 A g^−1^. Such results collectively highlight the importance of interfacial water regulation in VOPO_4_-based cathodes.

Similarly, alkali-metal pre-insertion has emerged as an effective approach to stabilize layered VOPO_4_ structures and enhance rate capability.^26^ K^+^ insertion leads to the formation of potassium vanadyl phosphate (KVOPO_4_), where the increased nanosheet thickness and expanded interlayer spacing facilitate reversible Zn^2+^ transport.^31,32^ This material delivers a high capacity of 179.3 mAh g^−1^ at 0.5 A g^−1^ and retains 153 mAh g^−1^ after 400 cycles at 1 A g^−1^. In parallel, Na^+^ pre-insertion combined with oxygen vacancy engineering yields Na_0.23_(VO_1−*x*_)_3_(PO_4_)_2.14_, demonstrating improved cycling stability and a discharge capacity of 75.3 mAh g^−1^ at 0.1 A g^−1^. These studies collectively suggest that synergistic effects between alkali-metal pillars and lattice defects are critical for stabilizing VOPO_4_ frameworks under repeated Zn^2+^ insertion/extraction.

Beyond pre-insertion strategies, defect engineering has been explored to enhance electrochemical kinetics. I_2_-mediated etching introduces controllable defect densities into VOPO_4_, producing materials, such as VOP-I4 and VOP-I8.^33^ Moderate defect introduction (VOP-I4) preserves the nanosheet morphology while significantly boosting capacity (249 mAh g^−1^ at 0.2 A g^−1^), whereas excessive etching (VOP-I8) leads to structural collapse and inferior performance.^34,35^ These results highlight the necessity of balancing defect concentration to optimize ion transport without compromising structural integrity.^106^

Organic intercalation presents another dimension for tuning VOPO_4_ electrochemistry. Using thioglycolic acid (TG) or ferrocene carboxylic acid (FC) as intercalators, stable organic–inorganic hybrid VOPO_4_ structures have been achieved with expanded basal spacing and altered vanadium valence states.^36,37^ Spectroscopic analyses, X-ray absorption near-edge structure (XANES) and Raman spectroscopy, revealed that TG intercalation induces partial reduction of V^5+^ to V^4+^, which may contribute to enhanced electronic conductivity and redox activity.^38,39^ It should be noted that this chemically induced V^5+^/V^4+^ redistribution does not automatically indicate fully reversible valence evolution during cycling; its reversibility should be verified by comparing charged and discharged states using *ex situ* or *operando* XANES/XPS. These findings illustrate how interlayer chemistry can directly influence both electronic structure and electrochemical behavior.

From a structure–property standpoint, intercalation strategies function primarily by expanding diffusion channels and weakening Zn^2+^–O interactions.^82^ Pre-inserted ions or crystal water acts as structural “pillars” and electrostatic buffers, reducing lattice strain during cycling and stabilizing layered or tunnel frameworks. Meanwhile, interlayer water molecules partially solvate Zn^2+^ in the host, lowering the desolvation energy and enabling faster solid-state diffusion.^87^

In addition to VOPO_4_ derivatives, researchers have begun exploring non-VOPO_4_ frameworks. For instance, the rocking-chair-type Zn_3_V_4_(PO_4_)_6_@C cathode leverages a stable open framework and Zn^2+^/H^+^ co-insertion mechanism, achieving 120 mAh g^−1^ at 0.04 A g^−1^ and retaining 80% capacity after 400 cycles at 1 A g^−1^.^107^ Other efforts include interlayer-engineered NH_4_V_4_O_10_ with pronounced pseudocapacitive behavior and flexible electrochemical stability, as well as highly crystalline hydrogen vanadium phosphate (HVPO) synthesized *via* a MIL-47 metal–organic framework template, which enables a well-defined proton-intercalation mechanism.^108^

More recently, high-entropy and amorphous design concepts have been introduced into vanadium phosphate systems. An *in situ* electrochemical oxidation activation strategy has been employed to construct isotropic high-entropy amorphous VO–PO@C, featuring abundant defect sites and enlarged ionic cluster radii.^106^ This structural complexity significantly accelerates Zn^2+^ solid-state diffusion, providing a new paradigm for overcoming kinetic limitations in vanadium-based cathodes.

Overall, current research demonstrates that the electrochemical performance of vanadium phosphate cathodes is highly sensitive to interlayer chemistry, defect density, and framework topology. While impressive capacities and cycling stabilities have been achieved, challenges remain in achieving a balanced combination of high-rate capability, long-term structural stability, and scalable synthesis. Future research should emphasize mechanism-guided material design, systematic comparisons across modification strategies, and deeper insights into Zn^2+^/H^+^ co-insertion behavior to fully unlock the potential of vanadium phosphates in AZIBs (Fig. 10).

**Fig. 10:**
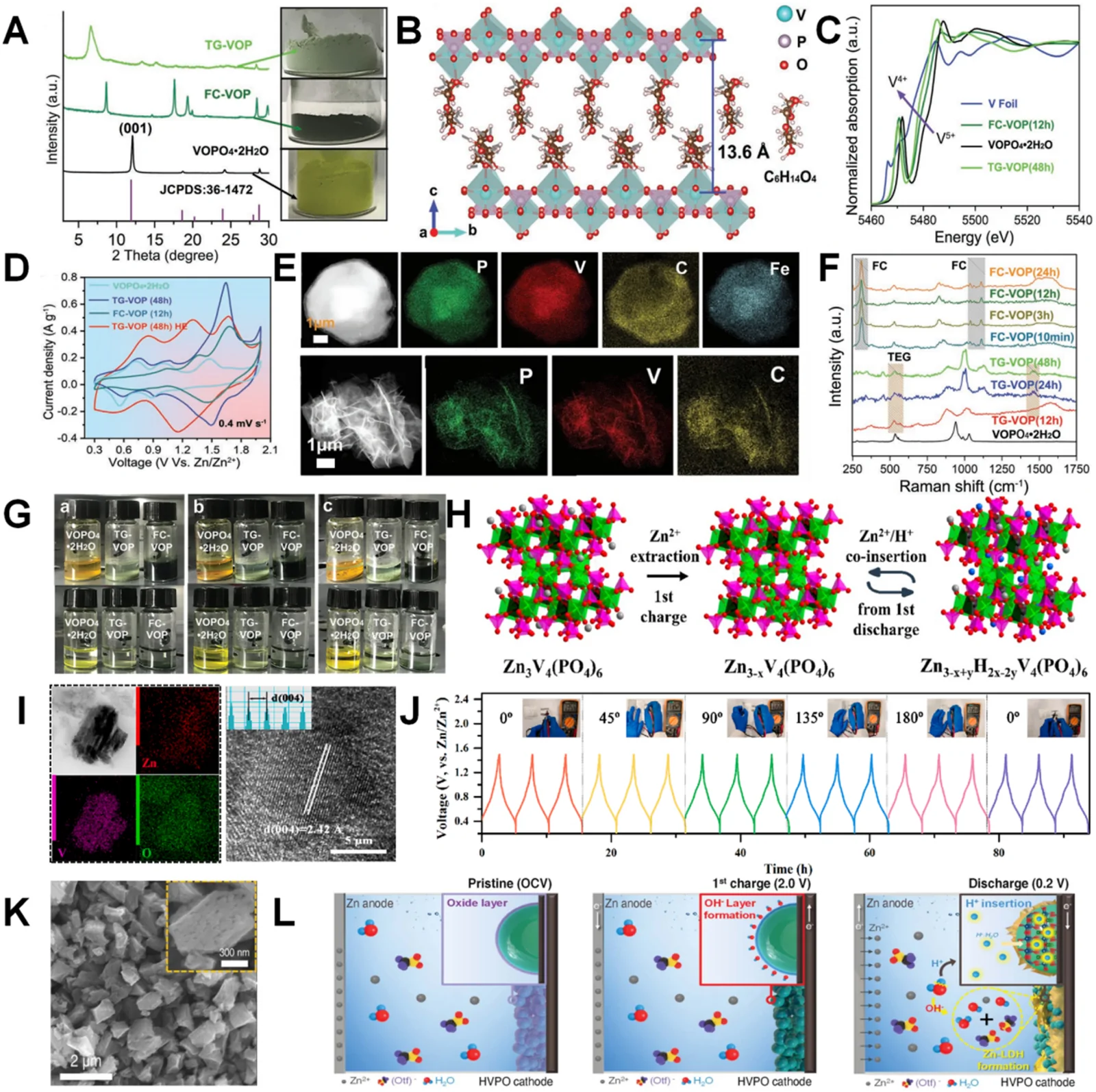
(A) XRD patterns of VOPO_4_·2H_2_O, FC-VOP, and TG-VOP. (B) TG-VOP crystal structure. (C) V K-edge XANES of V foil, VOPO_4_·2H_2_O, TG-VOP (48 h), and FC-VOP (12 h). (D) CV curves: VOPO_4_·2H_2_O, FC-VOP (12 h), and TG-VOP (48 h) in 3 M Zn(Tr)_2_; TG-VOP (48 h) in hybrid electrolytes. (E) FC-VOP samples and TEM mapping of TG-VOP. (F) Raman spectra of VOPO_4_·2H_2_O, TG-intercalated VOPO_4_, and FC-intercalated VOPO_4_ samples. (G) Photos of dispersed powders in 3 M Zn(Tr)_2_/hybrid electrolytes after 1, 6, and 15 days (top: Zn(Tr)_2_; bottom: hybrid).^109^ (H) ZVP cathode phase transition schematic (gray: Zn1; black: Zn2; green: V; purple: P; red: O; blue: H).^110^ (I) HRTEM of P-NVO-2 and P-NVO-2 elemental distribution. (J) SPZIB GCD at 0.2 A g^−1^ under bending; post-charge voltages.^111^ (K) Field emission scanning electron microscopy (FESEM) image; (L) HVPO proton-intercalation mechanism schematic.^112^ Adapted from ref. 109 with permission from Wiley-VCH, copyright 2022; ref. 110 with permission from the American Chemical Society, copyright 2022; ref. 111 with permission from Elsevier, copyright 2023; and ref. 112 with permission from the Royal Society of Chemistry, copyright 2022.

#### 2.2.2 Vanadium sulfides

One research team examined VS_4_ as a cathode for AZIBs. They refined the original VS_4_ structure using engineering strategies, employing a basic hydrothermal process to adjust its microstructure, thereby increasing active sites and facilitating Zn^2+^ ion movement.^113^

Another group utilized VS_2_, which shares structural similarities with VS_4_, by incorporating VS_2_ nanosheets into Ti_3_C_2_T_*x*_ to form a composite electrode. The intercalation of nanosheets expands the interlayer spacing, establishing a stable two-dimensional heterostructure. This layered heterostructure supports electrolyte infiltration in aqueous systems, boosts electron/ion conductivity, improves structural resilience, and supplies numerous sites for zinc ion accommodation. At a low current density of 0.2 A g^−1^, the battery's initial discharge capacity reaches 285 mAh g^−1^.^114^

Additionally, three variants of vanadium sulfide/reduced graphene oxide (V_*x*_S_8_/rGO, where *x* = 2, 5, or 6) were synthesized, each requiring charging above 1.8 V for electrochemical activation. Prior to activation, V_5_S_8_/rGO (50.4 m^2^ g^−1^) and V_6_S_8_/rGO (47.4 m^2^ g^−1^) had higher specific surface areas than V_2_S_8_/rGO (5.4 m^2^ g^−1^). Post-activation, however, V_5_S_8_/rGO and V_6_S_8_/rGO underwent structural and morphological changes, resulting in only 70% capacity retention after 700 cycles at 5 A g^−1^. In contrast, V_2_S_8_/rGO achieved 70% retention under the same conditions.^115^

Gao *et al.* synthesized VS_2_/V_2_O_5_·3H_2_O, in which VS_2_ and V_2_O_5_·2H_2_O coexist. The structure water in V_2_O_5_·3H_2_O can weaken the electrostatic interaction to accelerate Zn^2+^ embedding/detaching. The schematic diagram of the SVO//Zn system is shown in Fig. 11A.^116^ Wang and his team designed different morphologies VS_2_, VS_2_-D, VS_2_-M, and VS_2_-E. The morphology of VS_2_-E presents a floral structure, with thickness decreasing to 20.53 nm (Fig. 11B). In the XPS spectrum, as shown in Fig. 11C and D, they found that there exists V^3+^. Although V^3+^ will cause a lower transfer of electrons and the lower capacity, it can enhance the electrical conductivity and form lattice imperfections. In electrochemistry, as shown in Fig. 11E, at high rates, the contribution of pseudocapacitance is proportional to the surface area. Therefore, VS_2_-E has the highest contribution of pseudocapacitance. What's more, VS-M has the best rate performance (Fig. 11F).^117^ In addition, Wu's team synthesized D-VS_4_, as shown in Fig. 11H. The material has excellent cycle stability. After activating D-VS_4_, it can retain 92% capacity after 3500 cycles (Fig. 11G). And the CV curves can prove that the mechanism is the multi-step insertion/deletion of zinc ions and the multi-step change of the V oxidation state process (Fig. 11J). In the HRTEM, as shown in Fig. 11I, the uniform distribution of V, S, Zn, and O proves that Zn^2+^ successfully inserts into the layer.^118^

**Fig. 11:**
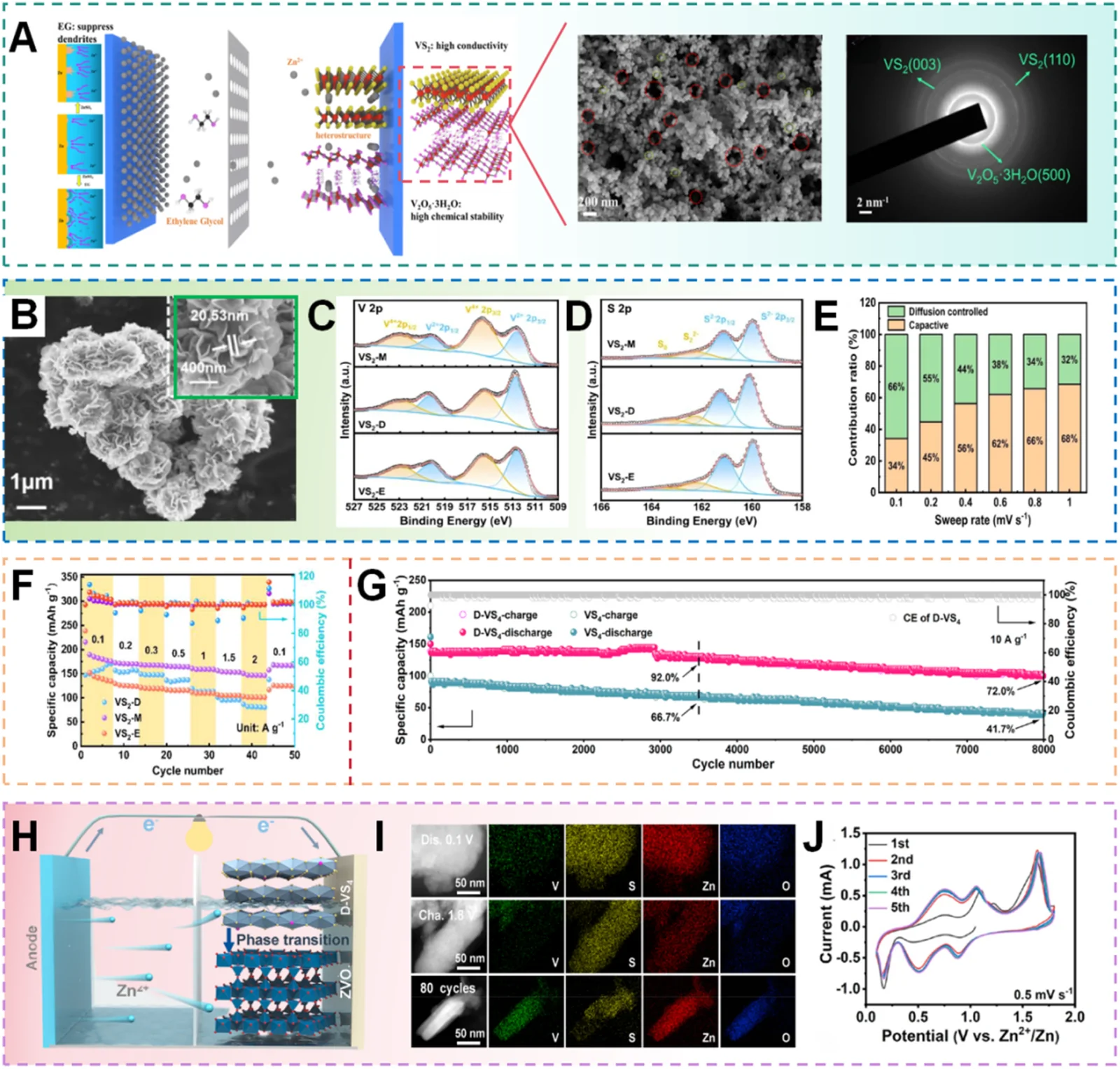
(A) Illustration of the charge and discharge processes in aqueous ZIBs using VS_2_ and V_2_O_5_·3H_2_O, SEM images of the SVO cathode after 6700 cycles in 41EZE electrolyte, and the SAED pattern of SVO.^116^ (B) High-resolution SEM images of VS_2_-E. (C) V 2p and (D) S 2p XPS spectra of VS_2_. (E) Capacitive and diffusion-limited capacity contributions for the VS_2_-M cathode. (F) Rate performance of VS_2_.^117^ (G) Cycling performance of D-VS_4_ and VS_4_. (H) Illustration of the Zn//D-VS_4_ battery. (I) HRTEM image of D-VS_4_. (J) CV curves of VS_4_ and D-VS_4_ at 0.5 mV s^−1^.^118^ Adapted from ref. 116 and 118 with permission from the American Chemical Society, copyright 2022 and 2023, respectively. Adapted from ref. 117 with permission from Elsevier, copyright 2024.

#### 2.2.3 Vanadium nitrides

Vanadium nitrides also serve as crucial cathode materials in this domain.

Starting with VN nanosheets, a simple self-sacrificial approach was used for void engineering, producing an interconnected framework with dual-component active ZnO quantum dots (QDs) decorating VN nanosheets (ZnO-QDs-VN-0.5). Drawing from the porous architecture of its precursor Zn_3_(OH)_2_(V_2_O_7_)(H_2_O)_2_, this material features a multi-porous design and strong electrochemical characteristics, reaching a capacity of 384.1 mAh g^−1^ at 0.1 A g^−1^, alongside favorable rate capabilities and extended cycle life.^119^ A similar QD decoration technique anchored vanadium oxynitride QDs onto nitrogen-doped graphene (VNO/graphene) to form a composite. This yields a specific capacity of up to 532 mAh g^−1^ at 0.2 A g^−1^, retaining 305 mAh g^−1^ after 800 cycles at 1 A g^−1^. The impressive rate performance and cycle durability arise from the woven structure of nitrogen-doped graphene, which prevents VNO aggregation and mitigates capacity fade to some extent.^77^ Beyond QD modifications, aluminum doping into VN has been explored to elevate cathode performance, achieving specific capacities as high as 544 mAh g^−1^ at 1 A g^−1^ and 275.6 mAh g^−1^ at 10 A g^−1^.^120^ Another method constructs vanadium nitride (VN) nanosheets on a titanium-based hollow nanoarray scaffold (denoted as VNTONC), addressing challenges like *in situ* activation, strong polarity of aqueous electrolytes, and cyclic expansion/contraction during efficient Zn^2+^ storage by generating multiple interfaces while preserving the VN (111) phase. The cathode evolves into a nanocrystalline form with enhanced active sites and conductivity, retaining much of the (111) facets for added Zn^2+^ storage. The prepared VNTONC electrode displays notable discharge capacities of 802.5 and 331.8 mAh g^−1^ at 0.5 and 6.0 A g^−1^, respectively.^121^ In addition, an *in situ* thermal conversion *via* pyrolysis of pentyl violet-intercalated V_2_O_5_ has led to innovative layered-stacked vanadium nitride nanocrystals/nitrogen-doped carbon hybrid nanosheets (VN/NC) as cathodes. Its stacked layering, leaf-like appearance, vanadium nitride nanocrystals, and conductive porous nitrogen-doped carbon nanosheets confer unique structural benefits, providing a specific capacity of 566 mAh g^−1^ at 0.2 A g^−1^ and sustaining 131 mAh g^−1^ after 1000 cycles at 10 A g^−1^, demonstrating exceptional rate performance and notable cycle stability.^63^ A thermal conversion strategy was also applied to prepare a V_2_O_3_ and VN composite (VOVN), with a lattice spacing of 0.35 nm and a specific surface area near 100 m^2^ g^−1^. The V_2_O_3_/VN heterointerfaces improve charge transport and reaction dynamics, resulting in a capacity of 445.3 mAh g^−1^ at 0.1 A g^−1^ and 151.4 mAh g^−1^ at a high current density of 30 A g^−1^.^122^

Bai *et al.* produced the nitrogen-vacancy-rich Nv-VN/C-SS-2 using a reduction strategy (as Fig. 12A). The assembled flexible quasi-solid-state Nv-VN/C-SS-2//Zn device retains 89.9% of its discharge time when bent to 180°, relative to the unbent state at 0° (Fig. 12B). The team used DFT to calculate, and the calculation results showed that Zn^2+^ could more rapidly adsorb/desorb on Nv-VN/C-SS-2 than on Nv-VN/C-SS-3, as shown in Fig. 12C, indicating that it can release more electrons into the delocalized electron cloud of the material to enhance the reversible capacity. The micromorphology of the nanorods of the material is shown in Fig. 12D, and it can be clearly observed. What's more, the SAED pattern (Fig. 12E) also shows the weak crystallinity of the material. In the long cycle electrochemical performance, as shown in Fig. 12F, Nv-VN/C-SS-2 retains the best specific capacity of 227 mAh g^−1^ after 1000 cycles. The vibrational characteristic peaks of VN in the Raman spectrum (Fig. 12G) showed similar trends and little change in intensity during the whole charge–discharge process, indicating that the structure is stable.^123^ Li and his team synthesized the 2D–2D VN–MoS_2_ nanosheets *via* hydrothermal and annealing processes, as shown in Fig. 12H. The surface composition of CC@VN–MoS_2_ films is similar to that of CC–MoS_2_. However, since the VN nanosheet is a substrate, the nanosheets were protruded, as shown in Fig. 12I. The graph of HAADF (Fig. 12J) shows that the elements Mo and S are enriched on the right side and the elements V and N are enriched on the left side, indicating the formation of the 2D material. The growth enhances the interaction with each other of the 2D materials, improving the efficiency of electron transfer. What's more, the SAED pattern shown in Fig. 12K also confirms that the material composed of MoS_2_ and VN.^124^ In addition, Dong *et al.* synthesised NVP/VN@NC *via* hydrothermal-assisted solution gelation. The storage mechanism of the material is that at the initial charge state, some of the Na^+^ were extracted from the Na2 site in Na_3_V_2_(PO_4_)_3,_ and the rest of the Na^+^ were still in the frame due to the inactive Na1 site to form Na_*x*_V_2_(PO_4_)_3_. After that, Zn^2+^/Na^+^ intercalate to form Zn_*m*_Na_*x*+_V_2_(PO_4_)_3_ and keep the NASICON stable (Fig. 12L). According to Fig. 12N, the formation of VN has been confirmed, and there are the dopants present in the form of pyridine N, graphite N, and nitrogen oxides. What's more, the material can also be applied in the SIBs. Collectively, these hydrated and alkali-ion-intercalated vanadium oxides reveal a common design rule: stable long-term cycling is achieved when interlayer species simultaneously serve as diffusion facilitators and structural stabilizers, whereas excessive interlayer expansion or weak host–guest interactions may lead to phase instability or capacity decay. Fig. 12M shows that the material has a strong reversible performance. Table 1 compares the synthesis methods and electrochemical performance of representative vanadium-based cathodes for AZIBs.^125^

**Fig. 12:**
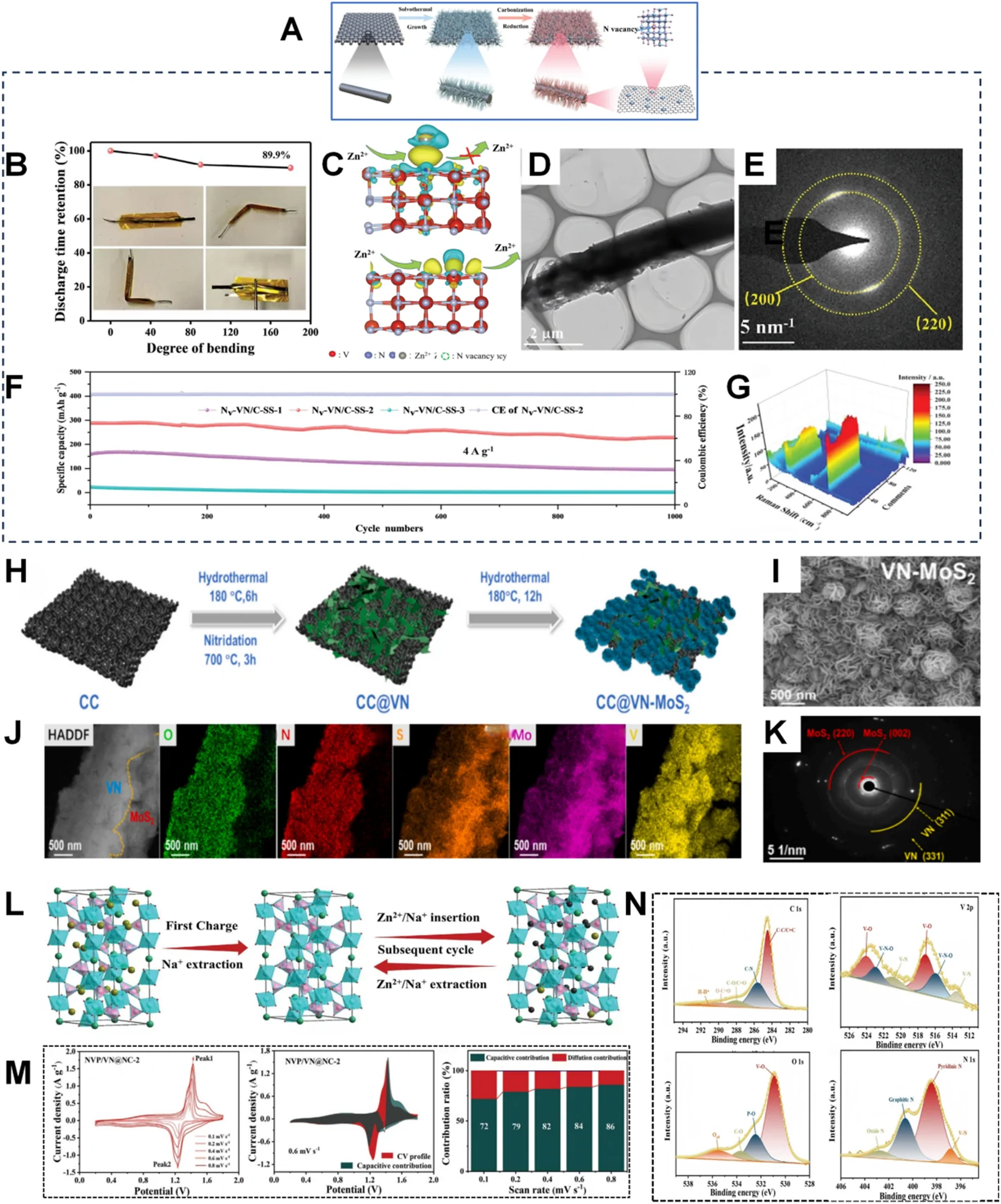
(A) Preparation schematic of Nv-VN/C-SS-2. (B) Soaking test of Nv-VN/C-SS-2. (C) Zn^2+^ charge density on Nv-VN/C-SS-3 and -2. (D) TEM of Nv-VN/C-SS-2. (E) SAED pattern of Nv-VN/C-SS-2. (F) Long-cycle performances of Nv-VN/C-SS-1, -2, and -3. (G) 3D *in situ* Raman mapping.^123^ (H) Synthesis schematic of the CC@VN–MoS_2_ electrode. (I) SEM images of CC@VN–MoS_2_. (J) EDX mapping of CC@VN–MoS_2_. (K) SAED patterns of CC@VN–MoS_2_.^124^ (L) Zn^2+^ insertion mechanism schematic. (M) CVs at various rates; diffusion/capacitive contribution at 0.6 mV s^−1^; ratio for NVP/VN@NC-2. (N) High-resolution V 2p, C 1s, O 1s, and N 1s XPS spectra of NVP/VN@NC-2.^125^ Adapted from ref. 123 and 125 with permission from Wiley-VCH, copyright 2024; and ref. 124 with permission from Elsevier, copyright 2024.

**Table 1:** Performance comparison of various vanadium-based cathodes for AZIBs

Material	Synthesis	Performance	Ref.
Zn_0.25_V_2_O_5_·*n*H_2_O	Microwave hydrothermal	260 mAh g^−1^ after 500 cycles	58
H_2_V_3_O_8_	One-step hydrothermal	94.3% retention after 1000 cycles	59
K_0.486_V_2_O_5_	Hydrothermal	171 mAh g^−1^ after 1400 cycles	70
Na_2_V_6_O_16_·1.63H_2_O	Hydrothermal	231 mAh g^−1^ after 100 cycles	71
CNVO	Sonication & annealing	2000 cycles at 10 A g^−1^	68
VOH@PEDOT	Hydrothermal & coating	432 mAh g^−1^ at 0.1 A g^−1^	84
V_2_O_5_/Ti_3_C_2_T*_x_*	Self-assembly	∼99.5% retention after 5000 cycles	81

### 2.3 Mechanistic challenges of vanadium-based cathodes in AZIBs

#### 2.3.1 Instability of vanadium oxides in aqueous electrolytes

Vanadium oxides are not chemically and electrochemically inert in aqueous electrolytes. Their instability comes down to how the V–O framework, vanadium's valence, and the electrolyte environment co-evolve during repeated cycling. With Zn^2+^/H^+^ constantly moving in and out, the V–O coordination around vanadium keeps warping repeatedly, which might cause some parts of the V–O framework to crack or rearrange. The co-evolution of the V–O framework, vanadium's valence states, and the electrolyte environment during repeated charge–discharge cycles drives their instability. When these species enter the electrolyte, irreversible vanadium dissolution sets in, and the electrochemically active material just keeps disappearing.^51,126^

Vanadium dissolution further triggers a series of degradation processes. One problem is that dissolved active vanadium species directly shrink the pool of redox centers, leading to nonstop capacity fading. Another issue is that as these species accumulate, they can change the electrolyte's color and composition, increase the charge-transfer resistance, and mess up interfacial ion transport. And finally, together with acidic microenvironments, the dissolved vanadium species can set off parasitic reactions on the Zn anode, including hydrogen evolution, Zn corrosion, and by-product buildup. Deep discharge, high-voltage overcharge, local pH changes, strongly solvating or complexing concentrated electrolytes, and long-term cycling that slowly wears away the surface protective layer—any of these can make these processes much more severe. Therefore, the stability of these vanadium oxide cathodes in AZIBs depends on more than the cathode's structural integrity. One must also regard it as a breakdown process that is shaped by the electrolyte and the whole-cell interactions.^30,127^

#### 2.3.2 Electrooxidation and activation of low-valent vanadium-based cathodes

Low-valent vanadium-based cathodes encompass V^3+^- or V^4+^-rich compounds, such as V_2_O_3_, VO_2_, V_6_O_13_, vanadium sulfides, vanadium nitrides, and some partially reduced vanadium oxides. These materials have lower initial oxidation states than V_2_O_5_-type high-valent vanadium oxides do. So, if you run them inside the positive potential window of aqueous Zn batteries, they show a much stronger drive toward electrochemical oxidation. The initial charge or activation step brings not only partial oxidation of low-valent V species to higher-valent V^4+^/V^5+^, but also a bunch of other changes: local coordination shifts, surface hydration, defect generation, and sometimes a partial rebuilding into more open or disordered vanadium oxide/hydroxide structures. This electrooxidation process can play a positive role in activating low-valent vanadium-based cathodes. The oxidation of V^3+^/V^4+^ to higher-valent V species provides additional redox-active centers and extends the accessible valence range during later cycles. Besides that, electrochemical activation can generate defects, enlarge ion-diffusion pathways, improve electrolyte wetting, and facilitate Zn^2+^/H^+^ transport. Lower charge transfer resistance and better reaction kinetics arise in some cases from the reconstructed surface layer, which behaves as a more electrochemically active interface.

But electrooxidation can backfire. Too much or poorly controlled oxidation may break down the original crystal structure, lead to irreversible phase transitions, and create soluble high-valent vanadium species. Active material loss and steady capacity fading, that's what you get when these species dissolve into the aqueous electrolyte. On top of that, repeated oxidation and reduction during cycling can put stress on the structure, cause particles to crumble, passivate the surface, and form an unstable interphase at the cathode/electrolyte interface. So for low-valent vanadium cathodes, electrooxidation can be helpful if kept mild, as it enhances electrochemical activity. But if it's excessive, it'll accelerate structural decay and vanadium dissolution. Either way, it's a double-edged activation process.

#### 2.3.3 Zn^2+^/H^+^ co-storage, by-product formation, and structural reconstruction

In aqueous Zn-ion batteries, Zn^2+^ does not act as the exclusive charge carrier within the cathode. With water around and local pH shifting during operation, protons can also take part in charge compensation upon discharge. Possible sources of these protons include water dissociation, hydrolysis reactions, or local acidification at the electrode/electrolyte boundary. As a result, many vanadium-based cathodes undergo Zn^2+^/H^+^ co-storage rather than pure Zn^2+^ intercalation.

The participation of H^+^ can be beneficial for electrochemical kinetics. Compared to Zn^2+^, H^+^ is not only smaller in size but also diffuses quicker. As a result, proton insertion is generally faster and can support high-rate capacity. What happens is that H^+^ co-insertion reduces the strong electrostatic repulsion you get from adding divalent Zn^2+^. As a result, the energy barrier for charge compensation drops, and local lattice strain is lessened. That means the capacity you get from vanadium-based cathodes isn't from just one thing, it's a mix of Zn^2+^ insertion, H^+^ insertion, surface redox processes, and maybe even some structural rebuilding.

The catch is that whenever H^+^ takes part, the local pH changes, and that can bring on by-products. During discharge, you can get basic zinc sulfate species like Zn_4_SO_4_(OH)_6_·*x*H_2_O when using a ZnSO_4_-based electrolyte. And if you switch to Zn(CF_3_SO_3_)_2_-based electrolyte, then similar basic compounds containing zinc triflate show up as well. Also, when dissolved vanadium species meet Zn^2+^, they can react and give rise to zinc pyrovanadate-type compounds—like Zn_3_V_2_O_7_(OH)_2_·*n*H_2_O, for example. These by-products can be both good and bad. On the plus side, reversible basic zinc salts help cushion local pH shifts and pitch in with charge compensation. But if too many by-products build up or they become irreversible, that's when trouble starts: they block ion pathways, raise interfacial resistance, coat the active sites, and eventually speed up capacity loss. The point is, for vanadium-based cathodes, you have to think about Zn^2+^/H^+^ co-storage, by-product formation, and structural changes as one package, not as separate pieces, when you're figuring out how they store energy.

#### 2.3.4 Zn^2+^ diffusion limitation and structural instability

Despite substantial research progress, vanadium-based materials still encounter fundamental challenges that hinder their practical application. These limitations, such as vanadium dissolution, slow Zn^2+^ diffusion kinetics, and structural instability, originate from intrinsic physicochemical properties and interfacial electrochemistry rather than mere processing factors.

Vanadium dissolution primarily arises from the over-reduction or over-oxidation of vanadium centers during deep cycling in aqueous electrolytes.^74^ When localized redox potentials fluctuate excessively, unstable intermediate species (*e.g.*, V^4+^ or hydrated VO^2+^ ions) tend to dissolve into the electrolyte through complexation with water or sulfate anions.^75^ Moreover, the high solvation ability of aqueous electrolytes accelerates the leaching of vanadium species, particularly under acidic or highly concentrated conditions. During discharge, this partial dissolution not only depletes active vanadium but also increases internal resistance and promotes irreversible capacity fading. Therefore, dissolution is electrochemically driven by unstable valence transitions and insufficient interfacial passivation.^76^

The sluggish Zn^2+^ diffusion kinetics in vanadium-based cathodes stem from the strong electrostatic interaction between divalent Zn^2+^ and oxygen atoms in VO_*x*_ polyhedra. This high charge density causes large polarization and energy barriers during intercalation. Additionally, the dense octahedral frameworks of vanadium oxides, while structurally robust, lack flexible diffusion tunnels, hindering rapid ion transport. The interfacial desolvation step further slows down reaction kinetics, since hydrated Zn^2+^ must shed its solvation shell before insertion, leading to limited rate capability. From a structure–property standpoint, the rigidity of V–O bonds and narrow diffusion channels collectively govern Zn^2+^ mobility.^77,78^

Repeated Zn^2+^ insertion/extraction generates lattice strain that induces irreversible phase transitions, especially between layered δ-V_2_O_5_ and amorphous or hydrated intermediates. These mechanical stresses often cause exfoliation, oxygen loss, and fragmentation at the nanoscale. The evolution of hydroxylated phases (*e.g.*, Zn_3_(OH)_2_V_2_O_7_·*x*H_2_O) further alters the crystalline order. Such instability is an inherent consequence of charge compensation mismatch; Zn^2+^ insertion demands local reduction of V^5+^ → V^3+^, while uneven electron localization distorts the lattice.^79^ Effective design thus requires balancing redox flexibility and structural resilience.

Understanding these mechanistic challenges provides guidance for targeted design. Stabilizing surface integrity through hydrophobic or polymeric coatings can suppress dissolution; creating defect or heterointerface structures modulates electron density and reduces polarization; and introducing pre-intercalated ions or water molecules expands diffusion channels and buffers strain. These strategies are not independent but rather are complementary, and they together mitigate the fundamental kinetic and thermodynamic limitations rooted in the electronic structure of vanadium compounds.

### 2.4 Modification mechanisms of vanadium-based cathodes in aqueous batteries

In the context of vanadium-based cathodes, modification mechanisms refer to how structural, electronic, and interfacial alterations regulate ion transport, redox reversibility, and phase stability during electrochemical cycling. Rather than merely enhancing capacity or rate performance empirically, each modification strategy targets specific physicochemical bottlenecks, such as Zn^2+^ diffusion barriers, vanadium dissolution, or lattice strain accumulation.^90^

Interlayer engineering and ion pre-intercalation primarily function by electrostatic shielding and lattice expansion, which weaken Zn^2+^–O interactions and reduce migration energy barriers.^91^ Pre-inserted ions or water molecules also act as structural pillars, buffering volume variation and maintaining framework integrity during repeated Zn^2+^ insertion/extraction. Thus, the performance enhancement originates from simultaneous kinetic acceleration and mechanical stabilization, rather than increased active material utilization alone. Defect engineering improves electrochemical performance through electronic structure modulation and localized ion-transport regulation. Oxygen vacancies or cation defects introduce additional electronic states near the Fermi level, enhancing electronic conductivity, while locally reducing electrostatic repulsion against incoming Zn^2+^.^97^ However, excessive defect densities may destabilize the lattice and promote vanadium dissolution, indicating that defect engineering requires controlled optimization rather than maximization. Surface modification strategies, including carbon coatings, polymers, and artificial interphases, regulate electrochemical behavior by controlling interfacial reaction pathways.^128^ These coatings suppress direct contact between the vanadium host and aqueous electrolyte, thereby inhibiting vanadium dissolution and side-product formation, while maintaining selective Zn^2+^ transport.^129^ Consequently, surface engineering modifies both interfacial thermodynamics and reaction kinetics, rather than serving solely as a conductive layer.

Heterostructure construction introduces built-in electric fields and interfacial charge redistribution, which promote directional electron transport and reduce charge-transfer resistance. The resulting interfacial polarization lowers Zn^2+^ diffusion barriers and stabilizes redox reactions at phase boundaries, making heterostructures particularly effective for high-rate aqueous batteries.^97,129^ Importantly, these modification mechanisms ultimately influence the energy-storage mechanism itself by regulating the balance between Zn^2+^ insertion, H^+^ co-intercalation, and conversion-type reactions.^100^ Structural flexibility and interfacial stability determine whether redox reactions proceed reversibly in the host lattice or trigger irreversible phase transformations.

In summary, the electrochemical improvements reported for vanadium-based cathodes arise from well-defined modification mechanisms that regulate ion transport, electronic conductivity, and structural stability at multiple length scales. A mechanistic understanding of how these strategies alter Zn^2+^ diffusion behavior and vanadium redox chemistry is essential for guiding future material design beyond empirical optimization.

### 2.5 Summary of modification strategies for vanadium-based cathodes in AZIBs

So overall, fixing vanadium-based cathodes in AZIBs isn't a single-knob tuning job. You've got separate issues to handle: slow Zn^2+^ diffusion, strong electrostatic attraction, vanadium leaching, shaky interfaces, and irreversible by-product accumulation. Therefore, drawing a comparative summary of representative modification strategies is a good way to understand both their mechanistic roles and their limitations (Table 2).

**Table 2:** Modification strategies and mechanistic roles of vanadium-based cathodes in AZIBs

Modification strategy	Targeted bottleneck	Mechanistic role	Possible limitation
Ion/water pre-intercalation	Strong Zn^2+^–O interaction and slow diffusion	Expands interlayer spacing, shields electrostatic interaction, and buffers lattice strain	Excessive expansion may weaken framework stability
Surface coating/interphase	Vanadium dissolution and side reactions	Suppresses direct contact with electrolyte and stabilizes surface V species	Thick coating may block Zn^2+^ transport
Defect engineering	Poor conductivity and limited active sites	Introduces local electronic states and lowers adsorption/diffusion barriers	Excessive defects may cause irreversible phase evolution
Heterostructure construction	Slow charge transfer and limited kinetics	Builds internal electric fields, redistributes charge, and accelerates electron/ion transport	Interfacial instability and complex synthesis
Electrolyte regulation	Dissolution, by-products, and unstable pH	Tunes the solvation structure, suppresses water activity, and stabilizes the interface	High cost or viscosity may reduce practical feasibility

### 2.6 Charge-carrier-dependent material design principles in aqueous batteries

Material design and selection in aqueous rechargeable batteries are fundamentally governed by the physicochemical characteristics of the charge carrier, including ionic radius, charge density, hydration structure, and desolvation energy.^130^ Consequently, cathode materials optimized for Zn^2+^ storage cannot be directly translated to systems employing NH_4_^+^ or Al^3+^ without re-evaluating the underlying ion–host interaction mechanisms. In Zn^2+^-based aqueous batteries, material design prioritizes mitigating strong electrostatic interactions between divalent Zn^2+^ and oxygen-rich frameworks.^131^ Layered or tunnel-structured vanadium compounds with expanded interlayer spacing, structural water, or pre-inserted ions are favored to lower Zn^2+^ diffusion barriers and accommodate lattice strain. Additionally, surface passivation and electronic structure modulation are essential to suppress vanadium dissolution induced by deep Zn^2+^ insertion.^132^ In contrast, NH_4_^+^-based aqueous batteries rely predominantly on hydrogen-bond-assisted insertion mechanisms rather than strong electrostatic coordination. Because NH_4_^+^ exhibits a lower charge density and smaller desolvation penalty, flexible frameworks with abundant oxygen or nitrogen functional groups are preferred to facilitate reversible hydrogen bonding. As a result, structural rigidity is less critical than framework adaptability and proton-coupled electron transfer kinetics.^133^ For multivalent Al^3+^ systems, the extremely high charge density and strong solvation shell impose severe kinetic constraints on solid-state diffusion. Effective material design therefore emphasizes open frameworks, large diffusion channels, and pseudocapacitive or surface-dominated storage mechanisms, rather than deep bulk intercalation.^134^ In this context, vanadium-based amorphous or defect-rich structures are more suitable than highly crystalline phases.

Collectively, these differences highlight that optimal material design in aqueous batteries must be charge-carrier-specific: Zn^2+^ systems require electrostatic shielding and lattice buffering, NH_4_^+^ systems benefit from hydrogen-bond-friendly and flexible hosts, while Al^3+^ systems demand open architectures that minimize desolvation and diffusion penalties. A universal cathode design is therefore unrealistic; instead, tailoring host structures to the intrinsic properties of the target ion is essential for advancing aqueous battery technologies.

## 3. Vanadium-based cathodes for aqueous ammonium-ion batteries (NH_4_^+^-IBs)

Aqueous ammonium-ion batteries (NH_4_^+^-IBs) have emerged as a promising class of energy storage systems, leveraging the abundance, low cost, and environmental benignity of ammonium ions (NH_4_^+^) as charge carriers.^54^ Unlike traditional lithium-ion batteries, NH_4_^+^-IBs utilize non-flammable aqueous electrolytes, enhancing safety and enabling sustainable applications in grid-scale storage and portable electronics. NH_4_^+^ exhibits a small hydrated ionic radius (∼3.31 Å) and low molar mass (18 g mol^−1^); although it is a monovalent ion, its diffusion kinetics are faster compared to multivalent ions like Zn^2+^ or Mg^2+^, while its weak hydrogen bonding in water minimizes desolvation energy barriers at electrode interfaces.^135^ Studies have shown that NH_4_^+^ exhibits unique energy storage mechanisms in aqueous batteries, where the insertion/deinsertion process of the electrode materials mainly relies on hydrogen-bond-assisted insertion mechanisms rather than the common strong electrostatic interactions of multivalent ions. This mechanism difference makes the NH_4_^+^ system an important control model, which helps to reveal the influence of carrier chemistry on electrode material design: for example, the dynamic characteristics of the hydrogen bond network can accelerate the diffusion kinetics of NH_4_^+^. However, a lot of challenges still confuse the researchers, such as limited cathode options, structural instability during cycling, and moderate energy. Therefore, more advanced materials are necessary.^130^ The key milestones in the development of NH_4_^+^-IBs from 2020 to 2025 are illustrated in Fig. 13. This section reviews key advancements in vanadium oxides, vanadate, and composite vanadium-based materials, highlighting their electrochemical mechanisms, structural modifications, and performance metrics in NH_4_^+^-IBs.

**Fig. 13:**
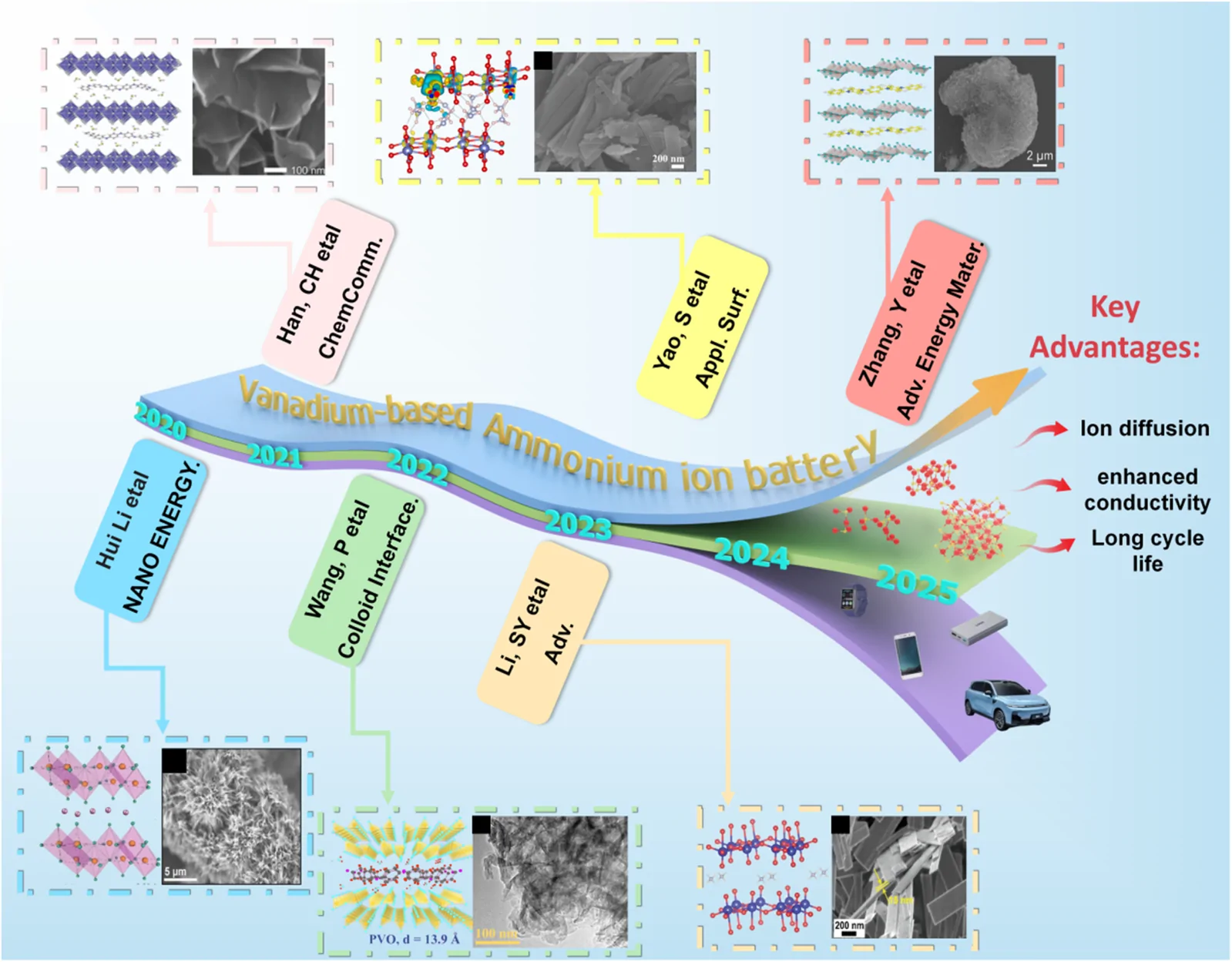
Chronological advancements in vanadium-based materials for NH_4_^+^-IBs. In 2020, a flexible full cell with a urchin-like NH_4_V_4_O_10_ cathode and a polyaniline anode in (NH_4_)_2_SO_4_ electrolyte was demonstrated. In 2021, polyaniline-intercalated vanadium oxide with 1.55 nm interlayer spacing was introduced as an electrode material. In 2022, PANI-intercalated HVO (PVO) expanded spacing to 13.9 Å for enhanced kinetics and stability. In 2023, NH_4_V_4_O_10_ charged to 1.6 V (NH_4_V_4_O_10_-C1.6) achieved high capacity (223 mAh g^−1^ at 10 A g^−1^) and 97.5% retention over 1000 cycles. In 2024, NH_4_V_4_O_10_ performance was boosted by partial ammonium removal and vanadium vacancies. In 2025, polyaniline-intercalated VO_*x*_ (P-VO_*x*_) was validated as an anode for NH_4_^+^ storage.^131–134,136,137^ Adapted from ref. 131, 133 and 136 with permission from Elsevier, copyright 2020, 2022 and 2024, respectively; ref. 132 with permission from the Royal Society of Chemistry, copyright 2022; and ref. 134 and 137 with permission from Wiley-VCH, copyright 2023 and 2025, respectively.

### 3.1 Charge-storage mechanism of NH_4_^+^ in vanadium-based cathodes

NH_4_^+^ storage in vanadium-based cathodes is fundamentally different from Zn^2+^, Mg^2+^, or Al^3+^ based storage. NH_4_^+^ is a monovalent tetrahedral molecular ion thatinteracts with V–O frameworks mainly through reversible N–H⋯O hydrogen bonds rather than strong metal–oxygen electrostatic coordination. The hydrogen-bond acceptor sites can include terminal V=O groups, bridging V–O units, interlayer water molecules, and O/N-containing functional groups introduced by polymers or conductive frameworks. During discharge, NH_4_^+^ insertion is accompanied by hydrogen-bond formation and partial vanadium redox reaction; during charging, hydrogen-bond cleavage enables NH_4_^+^ extraction and structural recovery.

This mechanism changes the design logic of vanadium-based AIB cathodes. Enlarging a rigid diffusion channel alone is insufficient; the host should provide a flexible layered environment, stable interlayer water, and abundant hydrogen-bond-friendly sites. Polymer intercalation, hydrated vanadium oxide frameworks, and conductive composites are therefore valuable not only because they enhance conductivity or expand spacing, but also because they regulate the reversibility and strength of the NH_4_^+^ host hydrogen-bond network. Excessively strong or irreversible hydrogen bonding, however, may induce structural fatigue and capacity decay.

### 3.2 Vanadium oxides

Vanadium-based oxides can reversibly convert between multiple oxidation states, which enables the transfer of multiple electrons during charging and discharging, making them capable of high-capacity charge storage. In addition, their low cost and abundant availability make them potential electrode materials for future energy storage systems.^138^

#### 3.2.1 V_2_O_5_

V_2_O_5_ and its derivatives have emerged as promising cathode materials for NH_4_^+^-IBs because their layered V–O frameworks provide both redox-active vanadium centers and hydrogen-bond acceptor sites for NH_4_^+^ intercalation. However, inherent limitations, such as low conductivity, structural instability, and strong hydrogen bonds, often hinder ion kinetics. Due to the characteristics of V_2_O_5_, the intercalation and doping strategies are effective methods to enhance the performance.

Intercalation of organic molecules has proven effective in expanding interlayer spacing and improving NH_4_^+^ diffusion. Zhang *et al.* synthesized ferrocene-embedded hydrated V_2_O_5_ (Fer/VOH), where ferrocene incorporation widens the interlayer distance, introduces oxygen vacancies, boosts conductivity, and weakens hydrogen bonds with NH_4_^+^. This yields a record capacity of 313 mAh g^−1^ at 0.2 A g^−1^ among vanadium oxides, with 54.8% retention (89 mAh g^−1^) after 2000 cycles at 2 A g^−1^. A full cell with a PTCDI anode underscores its practical potential^139^ (Fig. 14D and F).

**Fig. 14:**
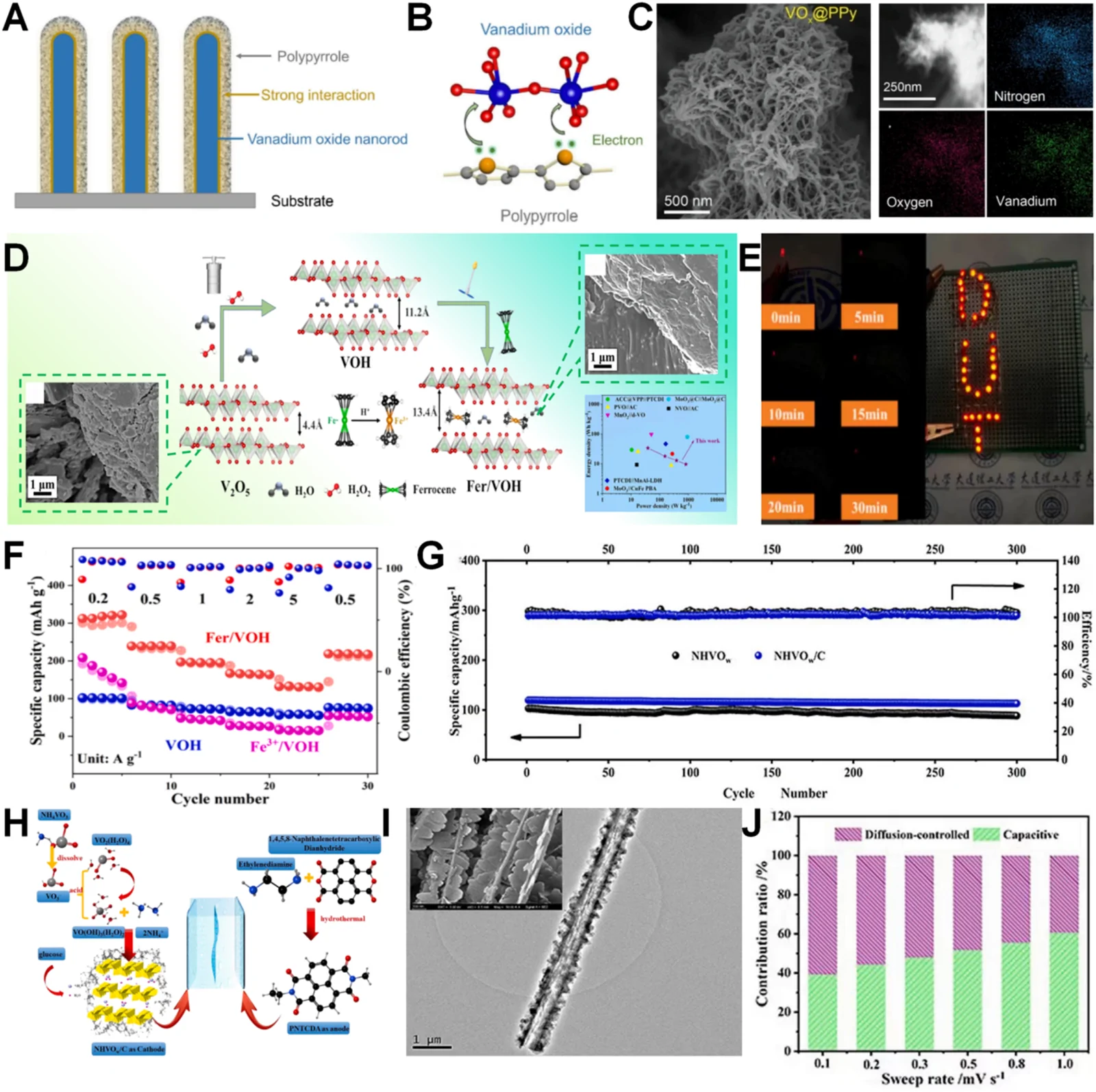
(A & B) Schematic diagrams of PPy–VO_*x*_ interaction. (C) SEM of VO_*x*_@PPy and STEM & elemental mapping (N, O, and V) of VO_*x*_@PPy.^151^ (D) Fer/VOH synthesis illustration; SEM of VOH/Fer/VOH; Ragone plot of PTCDI//Fer/VOH. (E) Battery lighting a LED. (F) Rate performance.^139^ (G) Long-cycle performance of NHVOw/C at 100 mA g^−1^. (H) Preparation flow for NHVOw/C & PNTCDA. (I) TEM of NHVOw/C. (J) Diffusion/capacitive ratios in NHVOw/C.^152^ Adapted from ref. 139, 151 and 152 with permission from Elsevier, copyright 2024, 2023 and 2023, respectively.

Similarly, Kuchena and Wang utilized *in situ* polymerization to intercalate polyaniline (PANI) into V_2_O_5_, forming nanoflower-like PANI–V_2_O_5_ (PVO) with an expanded interlayer spacing of 13.99 Å. This accommodates NH_4_^+^ ions (ionic radius 1.48 Å and hydrated radius 3.31 Å) and leverages PANI's π-conjugated structure for enhanced kinetics. Optimized PVO (PVO60) delivers 192.5 mAh g^−1^ at 1 A g^−1^ and 39 mAh g^−1^ at 20 A g^−1^, with 98% retention after cycling at 10–20 A g^−1^. *Ex situ* analyses confirm that hydrogen bond formation/breaking dominates the reversible NH_4_^+^ (de)intercalation, preserving structural integrity.^140^

Embedding conductive metal–organic frameworks (MOFs) offers another avenue for structural stabilization. Zhang *et al.* developed Ni-BTA/VOH by intercalating one-dimensional Ni-based MOF (ligand: 1,2,4,5-benzenetetramine) into hydrated V_2_O_5_ layers, increasing spacing to 13.8 Å and enhancing conductivity. The composite exhibits 183 mAh g^−1^ at 0.2 A g^−1^. In the cycle tests, it can achieve 52.6% retention after 500 cycles at 2 A g^−1^. The excellent electrochemical performance is benefited from synergistic VOH redox and C–N rearrangement mechanisms.^141^

Shifting attention to doping strategies, doping with metal ions addresses conductivity issues. Lin *et al.* hydrothermally synthesized Co-doped V_2_O_5_ nanorods, where 2% Co (as Co_3_O_4_) reduces agglomeration and improves electron transfer. In V_2_O_5_-saturated (NH_4_)_2_SO_4_ electrolyte, V_2_O_5_–2Co achieves 172 mAh g^−1^ at 1 A g^−1^, with 61.6% retention after 500 cycles and an energy density of ∼152 Wh kg^−1^.^142^

#### 3.2.2 VO_2_

VO_2_, with its tunnel-like and polymorphic structures, has shown considerable promise as a cathode for AIBs because its V–O framework can accommodate NH_4_^+^ migration through hydrogen-bond-assisted insertion. However, due to the challenges, such as limited conductivity and structural instability, it's necessary to modify through doping and composite formation to enhance ion kinetics and stability.^143–146^

The VO_2_@V_2_C MXene heterostructure (VO@VCM) is synthesized through one-step hydrothermal oxidation of V_2_C MXene (12 h optimal). The heterostructure integrates conductive MXene sheets with active VO_2_ nanorods (0.2–1 µm long). SEM/TEM reveal layered V_2_C (0.22 nm (103) fringes) coated with VO_2_ (0.62 nm (001) fringes), with EDS confirming uniform V/C/O distribution and residual F. The XRD pattern matches VO_2_(B) (JCPDS 31-1438) and V_2_C phases; XPS shows dominant V_4_^+^ (516.7/524.1 eV) with V_3_^+^ traces; Raman peaks (142, 194, and 993 cm^−1^) verify V–O bonds. In 2 M (NH_4_)_2_SO_4_ electrolyte (*vs.* Ag/AgCl), VO@VCM delivers 226 mAh g^−1^ at 0.1 A g^−1^, 85% retention after 5000 cycles at 5 A g^−1^, and rate performance of 120 mAh g^−1^ at 10 A g^−1^. Synergistic conductivity (MXene) and sites (VO_2_) enable NH_4_^+^ pseudocapacitance, with interlayer expansion (10.8–12.9 Å) aiding diffusion.^147^

Iron-doped VO_2_(B)/V_3_O_5_ (Fe_0.1_VO_2_(B)/V_3_O_5_), prepared solvothermally with calcination, forms hierarchical microflowers (∼5–10 µm) of intertwined VO_2_(B) nanorods (∼200 nm) and V_3_O_5_ nanosheets (∼50 nm). Fe^3+^ doping induces vacancies (EPR *g* = 2.002) and lattice shifts (XRD (110) to 14.5°); HRTEM shows 0.596 nm (VO_2_(B) (200)) and 0.363 nm (V_3_O_5_ (221)) fringes. In 2 M ZnCl_2_ + 0.5 M Zn(CH_3_COO)_2_ (*vs.* Ag/AgCl), it achieves 143.8 mAh g^−1^ at 0.5 A g^−1^, 85.2% retention after 5000 cycles at 2 A g^−1^, and a full-cell energy density of 115.1 Wh kg^−1^ with a PTCDI anode (78% after 1000 cycles). *Ex situ* XRD/FTIR confirms the phase transition (VO_2_(B) → (NH_4_)_*x*_V_2_O_5_·*n*H_2_O at ∼0.8 V); DFT shows Fe narrows the bandgap (1.85 eV) and lowers the NH_4_^+^ barrier (0.32 eV), promoting catalytic insertion.^148^

#### 3.2.3 Other vanadium oxides

Beyond the extensively studied VO_2_ and V_2_O_5_, other vanadium oxides, particularly hydrated variants, such as H_2_V_3_O_8_ and hydrated vanadium oxide (HVO), have garnered attention for their unique layered architectures that facilitate efficient ion intercalation in aqueous batteries. These materials often exhibit mixed valence states (V^4+^/V^5+^) and incorporate structural water or pre-intercalated species, which enhance lattice spacing, mitigate electrostatic repulsions, and improve structural stability during cycling. Strategies like doping, composite formation, and polymer intercalation have been pivotal in optimizing their electronic properties and ion diffusion kinetics for NH_4_^+^ storage.

The intercalation of polyaniline (PANI) is a commonly used method to enhance the storage performance of vanadium oxides for ammonium ions. Wang *et al.* used a dual polymer method to embed polyaniline into HVO, thereby forming polyaniline-intercalated HVO (PVO). This resulted in an increase in the interlayer spacing of HVO from 11.0 Å to 13.9 Å. This promoted the insertion and removal of ammonium ions and stabilized the material structure. Furthermore, the coexistence of polyaniline and crystalline water can also form a shielding effect that blocks the electrostatic interaction between the ammonium ions and the vanadium layer, thereby facilitating the diffusion of ions. HRTEM confirms that the widened (001) plane distance is about 13.85 Å. During discharge, NH_4_^+^ intercalates to form hydrogen bonds, as evidenced by *in situ* characterization. In the PVA/NH_4_Cl quasi-solid-state electrolyte, PVO retains ∼82% capacitance after 2000 cycles and ∼56% after 10 000 cycles, outperforming liquid electrolytes.^132^

Complementing this, Han *et al.* developed a PANI-intercalated vanadium oxide with 1.55 nm interlayer spacing, achieving a record capacity of ∼307 mAh g^−1^ at 0.5 A g^−1^. Lattice fringes align with XRD (001) peaks, exceeding HVO's spacing. *In situ* FT-IR shows N–H stretching at 3285 cm^−1^ intensifying and shifting during discharge, indicating NH_4_^+^ embedding and hydrogen bond formation with V=O groups; charging reverses this, breaking these bonds. XPS confirms reversible NH_4_^+^ (de)intercalation *via* hydrogen bond dynamics, establishing this as a benchmark for high-capacity NH_4_^+^-ion batteries.^133,149,150^

Mu *et al.* synthesized PPy–VO_*x*_, which exhibits the interaction between PPy and VO_*x*_. The interaction can enhance the electrochemical performance of VO_*x*_@PPy, as shown in Fig. 14A and B. What's more, the PPy didn't significantly change the structure of VO_*x*_, the highly porous structure has been retained (Fig. 14C).^151^ Zhang *et al.* synthesized NHVOw/C (Fig. 14H). The TEM image (Fig. 14I) shows us that the carbon is grown on NHVOw nanorods and distributed uniformly. In electrochemical performance, the NHVOw/C cathode can retain a high capacity retention of 93.7% after 300 cycles (Fig. 14G), and Fig. 14J also shows that the diffusion-controlled contribution is beneficial to show the excellent rate capability.^152^

### 3.3 Other vanadium-based cathode materials

Beyond vanadium oxides, other vanadium-based compounds, including vanadates and sulfides, have been investigated as cathodes for AIBs, capitalizing on their layered frameworks to enable effective NH_4_^+^ intercalation and enhanced electrochemical performance.

Vanadium sulfides, particularly in heterostructures, offer promising avenues for high-energy storage. Zhang *et al.* fabricated a network-like 1T-VS_2_/MXene heterostructure (HS-1T-VS@MXene) through *in situ* growth of 1T-VS_2_ nanosheets on Ti_3_C_2_T_*x*_ MXene *via* a one-step hydrothermal process. This architecture mitigates electron–hole recombination and facilitates carrier involvement in reactions. The MXene serves as a conductive backbone, promoting NH_4_^+^ mobility and penetration, while the 1T-VS_2_ nanosheets shorten ion transport paths. Crucially, NH_4_^+^ forms hydrogen bonds with surface functional groups (–OH and –O) on MXene and S atoms on 1T-VS_2_, bolstering structural stability and expanding charge pathways. The heterostructure interface further accelerates electron transfer, improving reaction kinetics. In an HS-1T-VS@MXene//AC-AAHSC device with 1 M (NH_4_)_2_SO_4_ electrolyte, specific discharge capacitances reach 96.0, 85.6, 78.6, 72.1, 62.0, 57.1, 53.6, 51.4, and 48.4 F g^−1^ at 1, 2, 3, 4, 5, 6, 8, 10, and 20 A g^−1^, respectively.^153^

Hydrated ammonium vanadates demonstrate structural resilience for NH_4_^+^ hosting. Zhang *et al.* synthesized (NH_4_)_2_V_6_O_16_·1.5H_2_O (NHVOw) nanorods and enhanced them through a surface carbon coating to form (NH_4_)_2_V_6_O_16_·1.5H_2_O/C (NHVOw/C). This method benefits from expanded interlayer spacing and improved conductivity. This composite retains the large vanadium layer gaps for NH_4_^+^ insertion/extraction and also has the characteristic of carbon's high conductivity, showing that the material can perform excellently in ammonium-ion systems. Rate tests in NH_4_Cl electrolyte show specific discharge capacities of 144.9, 114.9, 88.7, 71.8, and 68 mAh g^−1^ at 50, 100, 200, 300, and 500 mA g^−1^, respectively.^152^

Ferric vanadates provide large interlayer spaces for reversible NH_4_^+^ accommodation. Xu *et al.* developed ferric vanadate oxide (FVO) nanosheets, which exhibit superior pseudoscience behavior with NH_4_^+^ compared with Na^+^ or K^+^. In (NH_4_)_2_SO_4_ electrolyte, FVO delivers a high specific capacity of 72.5 mAh g^−1^ over 500 cycles at 5 A g^−1^, demonstrating better capacity than in Na_2_SO_4_ (64.7 mAh g^−1^) and K_2_SO_4_ (17.3 mAh g^−1^). This enhancement stems from directional hydrogen bonding between intercalated NH_4_^+^ and structural water in FVO, improving efficient ion diffusion and stronger host connections.^154^ Composites with carbon materials further boost surface area and capacitance. Murugesan *et al.* pioneered an ammonium vanadium oxide (NVO) and porous activated carbon (PAC) nanocomposite for NH_4_^+^ storage. The NVO–PAC exhibits a pore size of 3.81 nm and volume of 0.134 cm^3^ g^−1^, exceeding NVO's 3.05 nm and 0.042 cm^3^ g^−1^, enhancing mesoporous characteristics for ion storage. It achieves a specific capacitance of 527 mF cm^−2^ at 2 mA cm^−2^, surpassing NVO's 367 mF cm^−2^.^155^

Flexible configurations extend practical utility. Li *et al.* assembled a flexible aqueous NH_4_^+^-ion full cell using urchin-like NH_4_V_4_O_10_ on carbon fiber as the cathode and polyaniline nanorods on carbon fiber as the anode in (NH_4_)_2_SO_4_ solution. NH_4_V_4_O_10_'s layered structure with wide *d*-spacing accommodates NH_4_^+^ reversibly. The cell yields capacities of 80, 74, 65, 58, and 54 mAh g^−1^ at 0.1, 0.3, 0.6, 0.8, and 1 A g^−1^, with coulombic efficiencies increasing from ∼86.2% to 97.3% as current density increases. It retains electrochemical integrity under various deformations.^156^

In addition, Sun's team synthesized NH_4_V_4_O_10_@CNT (Fig. 15A and B)^157^ and Chen's team synthesized NVO_2_/CNT, which can achieve 472.5 mAh g^−1^ at 0.1 A g^−1^ (Fig. 15C–I).^158^

**Fig. 15:**
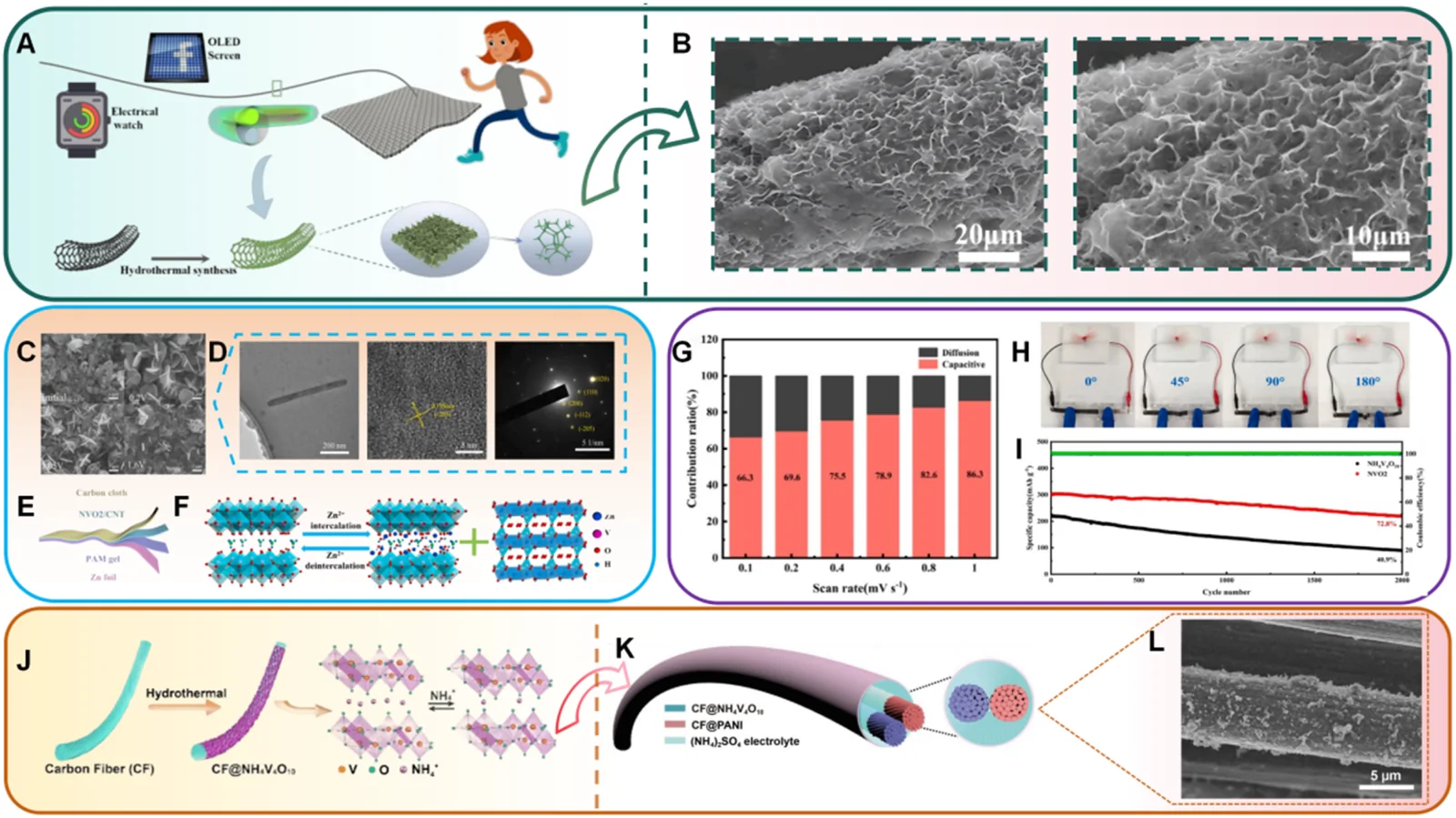
(A) Schematic of the NH_4_V_4_O_10_@CNT electrode & flexible energy storage. (B) SEM of NH_4_V_4_O_10_@CNT fiber at varying magnifications.^157^. (C) SEM of the NVO_2_ electrode at discharge/charge states. (D) TEM, HRTEM, & SAED of NVO_2_. (E) Quasi-solid-state ZIB schematic with the NVO_2_/CNT cathode. (F) Zn^2+^ storage mechanism schematic for NVO_2_. (G) Capacitive contribution percentages of NVO_2_ at various sweep rates. (H) Quasi-solid-state flexible ZIBs driving a small fan. (I) Cycling life of NH_4_V_4_O_10_ & NVO_2_ at 5 A g^−1^.^158^ (J) Schematic of CF@urchin-like NH_4_V_4_O_10_. (K) Schematic of CF@urchin-like NH_4_V_4_O_10_. (L) SEM images of NH_4_V_4_O_10_.^131^ Adapted from ref. 157 with permission from MDPI, copyright 2022; and ref. 131 and 158 with permission from Elsevier, copyright 2020 and 2023, respectively.

The electrochemical performance of representative vanadium-based cathodes for NH_4_^+^-IBs is summarized in Table 3. Overall, NH_4_^+^ storage in vanadium-based cathodes is governed primarily by the reversibility of hydrogen-bond formation/breaking and by the flexibility of the host framework. Thus, the core design criterion for NH_4_^+^-IB cathodes is not simply maximizing interlayer spacing or capacity, but constructing a reversible hydrogen-bond network that preserves vanadium redox activity during long-term cycling.

**Table 3:** Performance comparison of various vanadium-based cathodes for NH_4_^+^-IBs

Material	Synthesis	Performance	Reference
Fer/VOH	Hydrothermal & intercalation	54.8% retention after 2000 cycles	139
PVO	*In situ* polymerization	98% retention over 100 cycles	140
Ni–BTA/VOH	Hydrothermal & MOF intercalation	52.5% retention after 500 cycles	141
VO_2_@V_2_C MXene	Hydrothermal oxidation	59% retention after 1000 cycles	147
Fe–VO_2_/V_3_O_5_	Solvothermal doping	Stable over 3000 cycles	148
PANI–HVO	One-step hydrothermal	∼307 mAh g^−1^ at 0.5 A g^−1^	132
NVO–PAC	Room-temperature & composite	100% retention after 10^4^ cycles	155

## 4. Vanadium-based materials for aqueous aluminum ion batteries

AAlIBs have emerged as promising alternatives to traditional lithium-ion batteries due to their inherent safety features, stemming from non-flammable aqueous electrolytes that mitigate the risks of thermal runaway and dendrite formation. Aluminum, being the most abundant metal in the Earth's crust, offers advantages in terms of low cost, environmental benignity, and high theoretical volumetric capacity (8046 mAh cm^−3^) and gravimetric capacity (2980 mAh g^−1^), enabled by its three-electron redox process. These advantages make AAlIBs a good candidate for large-scale energy storage applications, such as grid-level systems and electric vehicles. However, AAlIBs also face significant challenges, including the narrow electrochemical stability window of water (about 1.23 V) and slow kinetics of highly charged Al^3+^ ions due to strong solvation effects and electrostatic repulsion. To find a material that can reversibly accommodate Al^3+^ without structural degradation or side reactions like hydrogen evolution is the main research direction. Vanadium-based compounds, with their layered or tunnel-like structures, multivalent redox capabilities (V^5+^/V^4+^/V^3+^), and tunable interlayer spacing, have shown great potential. It can address these limitations by facilitating efficient ion intercalation, enhancing capacity, and improving cycling stability in AAlIBs. This section reviews recent advancements in vanadium-based cathode materials for AAlIBs, paying more attention to vanadium oxides, vanadates, and other derivatives, and concludes their structural modifications, electrochemical performance, and underlying mechanisms.^19,159,160^

### 4.1 Charge-storage mechanism of Al^3+^ in vanadium-based cathodes

In vanadium-based AAlIB cathodes, charge storage should not be simplified as pure bulk Al^3+^ intercalation. Al^3+^ has an extremely high charge density and a strong hydration shell in aqueous electrolytes, rendering its desolvation and migration within rigid VO_*x*_ lattices kinetically unfavorable. Strong Al–O interactions can also induce local lattice distortion, irreversible phase evolution, and interfacial by-product formation. Accordingly, many reported Al-storage processes in vanadium-based cathodes involve coupled Al^3+^/H^+^ storage, surface-dominated redox reactions, pseudocapacitive contributions, or localized conversion-type reactions rather than deep and fully reversible Al^3+^ insertion.

This mechanistic constraint determines the preferred material-design strategy for AAlIB cathodes. Open frameworks, amorphous or defect-rich phases, MXene/carbon composites, and conductive heterointerfaces are more suitable than dense crystalline hosts because they reduce diffusion lengths, expose accessible redox sites, and tolerate local structural reconstruction. Therefore, the key objective in AAlIB cathode design is to lower reaction resistance and stabilize interfacial redox chemistry, rather than forcing extensive Al^3+^ insertion into a rigid V–O framework.

### 4.2 V_2_O_5_

The orthorhombic phase of V_2_O_5_, its layered structure, and high redox potential from V^5+^, stand out as features of a preferred cathode material in rechargeable batteries. This structure supports efficient ion intercalation, making it especially valuable for AAlIBs, because strong charge storage and structural durability are essential.^161^ In a key study, J. R. and team utilized a xerogel method to produce orthorhombic V_2_O_5_ for AAlIB applications. The gel-state V_2_O_5_ absorbs water at the oxide–air boundary, effectively inserting H_2_O molecules into the V_2_O_5_ interlayers due to its layered nature. This intercalation boosts electrochemical performance. Thus the material can achieve a specific capacity of120 mAh g^−1^ at 60 mA g^−1^. The water is added to expand interlayer distances and reduce electrostatic barriers to enhance ion diffusion and overall battery efficiency.^162^

Besides the intercalation, composite designs have also shown promise. Yang's group takes advantage of the superior conductivity of 2D MXene layers, creating Ti_3_C_2_ phases by HF etching Ti_3_AlC_2_ and combining them with rod-shaped V_2_O_5_ to form a 3D V_2_O_5_@MXene cathode. This setup makes use of reversible proton (H^+^) and aluminum ion (Al^3+^) insertion/extraction, greatly improving V_2_O_5_'s electrochemical reactivity. The result is an outstanding initial specific capacity of 626 mAh g^−1^ at 0.1 A g^−1^. The MXene scaffold offers conductive networks that lower resistance, while the interfacial layering accelerates ion movement and curbs degradation over cycles. 3D V_2_O_5_@Ti_3_AlC_2_ can overcome the typical drawbacks of standalone V_2_O_5_.^163^

Complementing this, defect engineering has emerged as another effective route. The Wang team introduced oxygen vacancies into V_2_O_5_ through a simple hydrothermal process with oxalic acid. According to the method, they successfully created vacancy-abundant O_v_-V_2_O_5_. These defects reduce reaction energy barriers and improve ion transport. During discharge, Al^3+^ and H^+^ insertion forms metastable phases like Al_*x*_V_2_O_5_ and H_*x*_V_2_O_5_. They are integrated within the V_2_O_5_ framework. These structures foster protective electrostatic bonds that reinforce the host and refine ion pathways. The material achieves a high initial capacity of 400 mAh g^−1^, retaining 103 mAh g^−1^ after 200 cycles at 0.4 A g^−1^. *Ex situ* analyses, including XRD and DFT computations, highlight how vacancies drive *in situ* activation, shedding light on Al^3+^ and H^+^ synergy in redox reactions. This vacancy modulation not only boosts performance but also deepens understanding of vanadium oxide metastability in AAlIBs.^164^

These innovations trace an evolution in V_2_O_5_ cathodes for AAlIBs, spanning hydrated xerogels, conductive hybrids, and vacancy-tuned variants. Capacities vary from 120 mAh g^−1^ in hydrated forms to over 600 mAh g^−1^ in MXene composites, tackling issues like sluggish kinetics and fading. Yet, integrating these, such as defects in MXene–V_2_O_5_ systems, could yield even greater advances in capacity and longevity.

### 4.3 VO_2_

Vanadium dioxide (VO_2_) is a versatile transition metal oxide. It exhibits multiple polymorphs with distinct structural and electronic properties, making it an attractive cathode material for aqueous rechargeable AAlIBs. Among its phases, such as rutile VO_2_(R), monoclinic VO_2_(M), tetragonal VO_2_(A), and bronze VO_2_(B), the latter stands out for energy storage applications due to its open framework that supports multivalent ion intercalation.^165^ VO_2_ generally consists of distorted VO_6_ octahedra, enabling tunnel-like pathways that facilitate ion diffusion while leveraging vanadium's variable oxidation states for high-capacity redox reactions. However, challenges like structural instability during cycling arise from strong Al^3+^–host electrostatic interactions. It's necessary to design structures that enhance durability and kinetics.^166^

Focusing on VO_2_(B), its bi-layer shear structure, formed by corner- and edge-shared VO_6_ octahedra, creates expansive tunnels (*e.g.*, 0.82 nm^2^ along the *b*-axis). VO_2_ is ideal for accommodating Al^3+^ ions (ionic radius: 53.5 pm) with minimal lattice strain. This configuration strengthens edge sharing to bolster resistance to shear distortions and promotes cycling stability.

Innovative structures have made use of these attributes. Cai and colleagues engineered VO_2_(B) holey nanobelts, exploiting the shear framework for enhanced stability. They also designed the 2D porous structure for expanded electrolyte interfaces, which shorten diffusion paths and boost reactivity. The cathode achieves 234 mAh g^−1^ at 0.15 A g^−1^ level. Furthermore, the material can retain 77.2% capacity after 1000 cycles at 1 A g^−1^, one of the highest performances for AIB cathodes. *Ex situ* studies and electrokinetic analyses indicate a mechanism involving reversible H^+^ and Al^3+^ co-intercalation/de-intercalation, with the holey structure mitigating volume changes.^62^

Similarly, Wang *et al.* synthesized nanobelt-like VO_2_(B) with 3D interconnected tunnels through a straightforward method, preserving the open architecture for swift Al^3+^ transport (Fig. 16G–I). It can achieve 235 mAh g^−1^ at 0.2 A g^−1^ and 49.3% retention at 2 A g^−1^. *Ex situ* XRD and XPS confirm dominant Al^3+^ intercalation, emphasizing the tunnels' role in overcoming charge density barriers.^167^

**Fig. 16:**
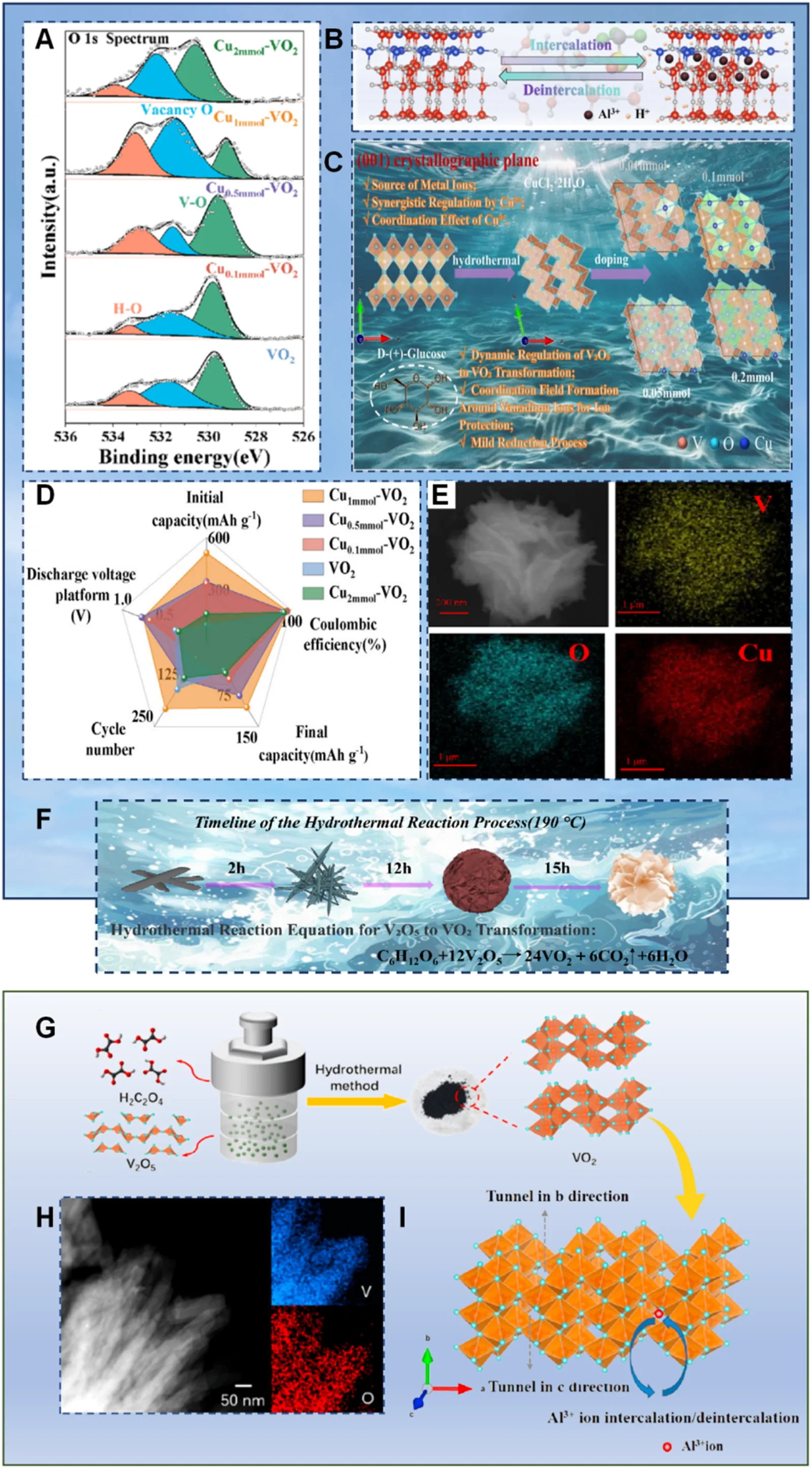
(A) XPS spectra of VO_2_ with varying doping degrees. (B) Energy storage mechanism in Cu_1 mmol_–VO_2_‖Al batteries. (C) Advantages of Cu_1 mmol_–VO_2_ cathodes. (D) Radar map comparing VO_2_ cathodes with doping levels (VO_2_, Cu_0.1/0.5/1/2 mmol_–VO_2_). (E) EDS mapping of Cu_1 mmol_–VO_2_. (F) Preparation and morphology evolution of Cu_1 mmol_–VO_2_.^168^ (G) Synthesis route of VO_2_. (H) Elemental mapping of VO_2_. Abbreviations: SEM (scanning electron microscopy), TEM (transmission electron microscopy), and XRD (X-ray diffractometry). (I) Al^3+^ intercalation/deintercalation mechanism.^167^ Adapted from ref. 168 with permission from Wiley-VCH, copyright 2025; and ref. 167 with permission from Elsevier, copyright 2021.

Doping and defect strategies have been integrated to achieve better capacity. Wang and his team used the glucose-assisted hydrothermal method to reduce monoclinic VO_2_ with Cu ion doping. This method can effectively promote the self-assembly of VO_2_ into a nanoflower structure (Fig. 16C and F). The team analysed the XPS spectrum (Fig. 16A) and found that at a moderate doping concentration, the amount of H–O bond and O vacancy reaches the maximum value; however, the strength of the V–O bond decreases to the minimum, indicating that Cu can distribute more uniformly in the lattice. According to Fig. 16E, the EDS mapping of Cu_1 mmol_–VO_2_ shows that the distribution of elements V, O, and Cu is uniform. Moreover, VO₂ samples with different Cu-doping levels exhibit different electrochemical performance, as shown in Fig. 16D. The storage mechanism is that the dual synergistic transformation induced by glucose optimized the crystal structure and reaction sites of VO_2_, while copper doping stabilized the lattice framework and enhanced electronic conductivity, providing effective support for the insertion/deinsertion reactions of Al^3+^, as shown in Fig. 16B.^168^

Wang *et al.* used glucose-assisted hydrothermal reduction of monoclinic VO_2_, with copper doping to drive nanoflower self-assembly. Cu introduces 3d orbitals that tune the V 3d–O 2p electronic transitions, enhancing conductivity and electron coupling. The resulting cathode achieves 421 mAh g^−1^ at 0.8 A g^−1^, benefiting from increased active sites and reduced dissolution in aqueous media.^169^

This evolution in VO_2_ cathodes, from polymorphic versatility to structure- and doping-optimized VO_2_(B), spans capacities of about 235–421 mAh g^−1^, tackling diffusion and stability hurdles. Future materials, like doped holey structures, could cooperate with these for superior AIB performance.

### 4.4 Vanadate

Beyond the aforementioned vanadium oxides, vanadates have become cathode materials with substantial promise for AAlIBs, benefiting from their layered architectures, tunable electronic structures, and capacity for multivalent ion accommodation. These compounds often include stabilizing cations or structural modifiers that reduce electrostatic repulsions and enhance ion diffusion to address key limitations in vanadium-based systems.

Research on ammonium vanadates highlights their potential through expanded interlayer spacing and composite designs. For example, Vishnu and colleagues synthesized NH_4_V_4_O_10_ (NVO) with an interlayer distance of 9.4 Å, which was achieved through a controlled process that promotes structural openness. This configuration simulates Al^3+^ intercalation, achieving a specific capacity of 210 mAh g^−1^ at 50 mA g^−1^. The enlarged spacing, which reduces diffusion barriers, can enable efficient charge storage without significant lattice strain.^64^ Except for this team, Wu's team developed (NH_4_)_2_V_10_O_25_·8H_2_O composited with Ti_3_C_2_T_*x*_ MXene, which remarkably improved cycling performance over the original NHVO structure. At 1.0 A g^−1^, it retains 268.7 mAh g^−1^ after 32 cycles, attributed to the formation of a stable storage phase (Al_12_V_8_O_37_·30H_2_O) supported by the MXene framework. This scaffold prevents dissolution and retains structural integrity during phase transitions to enhance reversibility.^170^ In a related advancement, Wu *et al.* utilized Mo_2_V_2_AlC_3_ MXene to *in situ* form V_0.95_Mo_0.97_O_5_, leveraging Mo–V bimetallic synergy to accelerate active ion diffusion and expand active sites. The resulting cathode achieves 334.06 mAh g^−1^ after 130 cycles at 0.4 A g^−1^, demonstrating superior stability through bimetallic bonding that induces favorable changes in the (100) crystal plane and supports reversible Al^3+^ intercalation.^171^

Shifting attention to bismuth vanadates, amorphous forms offer unique advantages by disrupting crystalline order. Sunny and coworkers explored amorphous Bi_4_V_2_O_11_, where the lack of long-range periodicity generates abundant defects, such as vacancies and voids, that serve as active sites and open channels for ion migration. This design achieves an initial discharge capacity of 272 mAh g^−1^ at 1 A g^−1^, retaining 130 mAh g^−1^ after 50 cycles. *Ex situ* analyses, including XRD, SEM, and XPS, suggest a mechanism involving Al^3+^ storage improved by isotropic defect pathways, outperforming its crystalline counterpart and highlighting the role of amorphous materials in overcoming kinetic hurdles in AIBs.^172^

Lithium vanadates, exemplified by LiV_3_O_8_, benefit from pre-intercalated Li^+^ ions that screen electrostatic interactions. Vaiyapuri Soundharrajan *et al.* investigated this layered material. Its classical vanadium-based features can enable high capacity and extend cycling for multivalent carriers. The interlayer Li^+^ reduces repulsions from external cations, resulting in a discharge capacity of 182 mAh g^−1^ at 0.59C (1C = 838 mAh g^−1^). Remarkably, it retains 114 mAh g^−1^ after 500 cycles at the same rate, though gradual structural deterioration linked to high charge density of Al^3+^ contributes to a 22.7% capacity loss. *Ex situ* XRD, XPS, SEM, and TEM reveal challenges in reversible (de)intercalation, providing insights for optimizing layered cathodes.^173^

Iron vanadates exhibit environmental sensitivity, particularly to electrolyte conditions. Sonal Kumar's group examined FeVO_4_, finding its performance to be modulated by pH. Elevating the pH from 1.9 to 3.5 minimizes visible electrolyte discoloration during cycling, indicative of reduced electrode dissolution. At higher pH, the cathode attains up to 350 mAh g^−1^, with synchrotron-based characterization techqniues (XRD, XPS, XAS, and Raman) confirming a conversion mechanism involving Al_*x*_V_*y*_O_4_ and Fe–O–Al formations. This pH-dependent stability underscores the need for tailored electrolytes to suppress side reactions in conversion-type cathodes.^174^

Finally, Prussian blue analogs (PBAs) incorporating vanadium represent a frontier in coordination-tuned cathodes. Feng and team, leveraging PBAs' open frameworks and adjustable environments, used an acid-assisted strategy with H_2_C_2_O_4_ to synthesize vanadium-based PBAs, specifically Fe-doped VFePBA. This method preserves the crystal coordination while boosting the proportion of electrochemically active sites, thereby elevating specific capacity. The material exhibits a narrow band gap and low Al^3+^ migration barrier (0.586 eV) to promote rapid ion conduction. In AIBs, it provides 103.34 mAh g^−1^ at 0.1 A g^−1^, with *in situ* studies confirming suppressed distortions through Al–O coordination and a capacitance-dominated mechanism.^159^

In addition, De *et al.* used a low-cost V_2_O_5_ interconnected flake morphology as the cathode material; it can achieve 140 mAh g^−1^ at 0.5 A g^−1^ and in the long cycles and it can retain 96% capacity after 1000 cycles (Fig. 17A and B).^175^

**Fig. 17:**
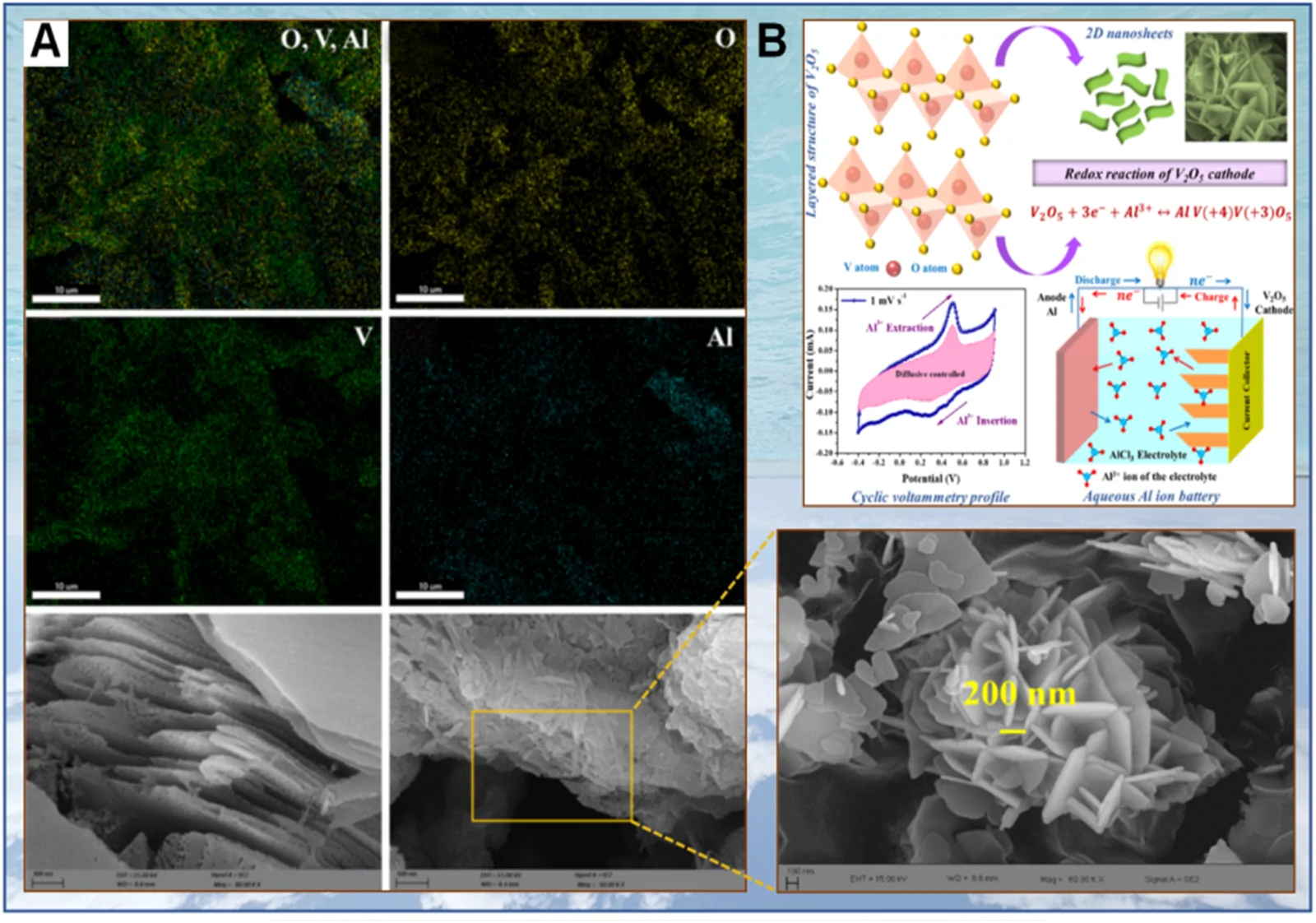
(A) SEM-EDS mapping and SEM images of the V_2_O_5_ electrode after discharge and 1000 cycles at 1 A g^−1^. (B) Overview of the V_2_O_5_ electrode.^175^ Adapted from ref. 175 with permission from the American Chemical Society, copyright 2023.

Collectively, these vanadate advancements, from ammonium and bismuth variants to lithium, iron, and PBA derivatives, span capacities of 103–350 mAh g^−1^. The attention is on strategies like interlayer expansion, compositing, amorphization, doping, and pH optimization to enhance kinetics and stability. Future integrations could use hybrid systems for superior AIB performance (Table 4).

**Table 4:** Performance comparison of various vanadium-based AAlIB cathodes

Material	Synthesis	Performance	Ref.
V_2_O_5_ xerogel	Ion exchange	∼120 mAh g^−1^ at 60 mA g^−1^	162
V_2_O_5_@MXene	van der Waals assembly	626 mAh g^−1^ at 0.1 A g^−1^	163
VO_2_(B) holey nanobelts	Hydrothermal	77.2% retention after 1000 cycles	62
Cu-Doped VO_2_	Hydrothermal doping	642 mAh g^−1^ at 0.4 A g^−1^	168
(NH_4_)_2_V_10_O_25_/MXene	Hydrothermal & self-assembly	688.8 mAh g^−1^ at 1 A g^−1^	170
LiV_3_O_8_	Sol–gel	77.3% retention after 500 cycles	173
FeVO_4_	Hydrothermal & calcination	High capacity but fades	174

In conclusion, the performance of vanadium-based AAlIB cathodes depends on whether the material can accommodate high-charge-density Al species through accessible surface or near-surface redox sites while avoiding irreversible lattice collapse. V_2_O_5_ and VO_2_(B) benefit from hydration, defects, and conductive scaffolds, whereas vanadates and amorphous or PBA-like frameworks provide more open coordination environments for Al-related storage. The common direction is therefore to combine open diffusion architecture with defect/interface engineering, rather than relying on bulk intercalation alone.

## 5. Vanadium-based cathodes for AMgIBs

AMgIBs offer several advantages over conventional LIBs, including enhanced safety due to non-flammable aqueous electrolytes that reduce fire risks and eliminate dendrite formation.^176,177^ They are more cost-effective and sustainable, leveraging abundant, low-cost magnesium resources and non-toxic compounds, which minimize environmental impact and supply chain vulnerabilities.^178^ Additionally, Mg^2+^ provides a higher theoretical volumetric capacity (3833 mAh cm^−3^*vs.* lithium's 2061 mAh cm^−3^), potentially enabling compact, high-energy designs.^178,179^

However, AMgIBs face key challenges, such as a narrow electrochemical stability window (∼1.23 V) limited by water decomposition, lower operating voltages than non-aqueous systems,^176^ sluggish Mg^2+^ diffusion kinetics caused by strong solvation, and anode corrosion in aqueous media.^180^ Vanadium-based cathodes may address part of these limitations, but their evaluation should focus on whether the host can support hydrated or partially desolvated Mg^2+^ transport and reversible Mg^2+^/H^+^ co-storage rather than on apparent high capacity alone.

### 5.1 Charge-storage mechanism of Mg^2+^ in vanadium-based cathodes

Although Mg^2+^ and Zn^2+^ are both divalent ions, Mg^2+^ generally carries a stronger hydration shell and faces a higher desolvation barrier. This makes Mg^2+^ diffusion in rigid VO_*x*_ frameworks slower and more sensitive to the local interlayer environment. Interlayer water is therefore not merely a spacing-expansion agent; it can shield Mg^2+^–O electrostatic interactions, assist hydrated or partially desolvated Mg^2+^ migration, and buffer lattice strain during repeated insertion/extraction. In many aqueous Mg systems, Mg^2+^ storage may also couple with H^+^ participation, making Mg^2+^/H^+^ co-storage and interfacial proton activity important mechanistic variables.

Accordingly, effective AMgIB cathode design should prioritize hydrated layered vanadium oxides, Mg^2+^/H_2_O pre-intercalated frameworks, heterostructures, and open channels that maintain water-assisted diffusion pathways. For Mg^2+^ systems, the central challenge is not simply to maximize theoretical capacity but to reduce desolvation and migration barriers while preserving structural reversibility under strong ion–host interactions.

### 5.2 Vanadium oxides

#### 5.2.1 V_2_O_5_

Orthorhombic V_2_O_5_ (α-V_2_O_5_) features a layered structure with edge- and corner-sharing distorted VO_5_ square pyramids stacked along the *c*-axis, yielding an interlayer spacing of 4.37 Å. This architecture positions it as a promising intercalation host for various ions in energy storage systems.^21^

In AMgIBs, Drosos *et al.* synthesized orthorhombic V_2_O_5_ nanostructures *via* aerosol-assisted chemical vapor deposition, demonstrating high cathode stability with unchanged peak intensities after over 1000 cycles. Rate evaluations at 2.4–5.9 A g^−1^ revealed superior capacities at higher rates (427 mAh g^−1^ at 5.9 A g^−1^*vs.* 170 mAh g^−1^ at 2.4 A g^−1^), with 99% retention upon returning to 5.9 A g^−1^, underscoring excellent structural integrity and reversibility.^181^ To address limitations like sluggish kinetics, modifications incorporating hydration and pre-intercalation have been pursued. Xu *et al.* developed bilayer-structured Mg_0.3_V_2_O_5_·1.1H_2_O nanowires through chemical modification of α-V_2_O_5_, where pre-intercalated Mg^2+^ enhances conductivity and stability, and lattice water promotes Mg^2+^ mobility *via* charge shielding. The synthesis involves H_2_O_2_-induced valence reduction and structural rearrangement, resulting in an 11.9 Å interlayer spacing similar to that of Zn_0.25_V_2_O_5_·*n*H_2_O. With a Brunauer–Emmett–Teller (BET) surface area of 38.23 m^2^ g^−1^ aiding ion transfer, it exhibits multistep Mg^2+^ intercalation, delivering capacities of 162–50 mAh g^−1^ at 0.1–4.0 A g^−1^, full retention at 1 A g^−1^ after 10 000 cycles, and 80% at 2 A g^−1^ with 99.91% coulombic efficiency.^60^ Building on this, He *et al.* integrated oxygen defect engineering into a 2D-on-2D nanopaper-like bilayer V_2_O_5_·*n*H_2_O xerogel (BL-HVOd NPS) to boost Mg^2+^ insertion/extraction kinetics and minimize irreversibility in AMgIBs. Interlayer water acts as spacers for providing stable expanded channels and electrostatic shielding. The material achieves a reversible capacity of 162.8 mAh g^−1^ at 0.2 A g^−1^, retaining 81.3 mAh g^−1^ (88%) after 2000 cycles at 10 A g^−1^.^182^

#### 5.2.2 Other vanadium oxides

Similar to V_2_O_5_, VO_2_ exhibits several polymorphs, including VO_2_(R), VO_2_(M), VO_2_(T), VO_2_(A), VO_2_(B), and VO_2_(C). Despite this diversity, VO_2_ has received limited attention in AMgIBs due to sluggish electrolyte ion transfer kinetics and structural instability stemming from narrow interlayer channels and confined multilayer stacking. To address these issues, Li *et al.* engineered a cation-intercalation strategy that controllably expands the interlayer spacing of VO_2_ from 6.17 to 6.49 Å through multivalent cation (Na^+^, Mg^2+^, and Al^3+^) insertion, while preserving the structural integrity of the nanoplate morphology. HRTEM analysis revealed well-defined lattice fringes with interlayer spacings: Al–VO_2_ (0.649 nm) > Mg–VO_2_ (0.639 nm) > Na–VO_2_ (0.633 nm) > VO_2_(0.617 nm), correlating with cation charge density (Al^3+^ > Mg^2+^ > Na^+^). Quantitative ICP analysis confirmed compositions of Na_0.27_VO_2_, Mg_0.21_VO_2_, and Al_0.34_VO_2_, designated as Na–VO_2_, Mg–VO_2_, and Al–VO_2_. Comparative CV analysis at varying scan rates showed Mg–VO_2_ with higher redox peak intensities and larger integrated areas. As current density increased from 1 to 5 A g^−1^ and returned to 1 A g^−1^, Mg–VO_2_ electrodes recovered nearly their initial discharge capacity, demonstrating good rate reversibility. Moreover, Mg–VO_2_ exhibited superior first discharge capacity and cycling stability at 1 A g^−1^ compared to other samples. The capacity enhancement in Na–VO_2_, Mg–VO_2_, and Al–VO_2_ arises from larger layer spacing due to metal ion intercalation, reducing the energy barrier for Mg^2+^ embedding. Mg–VO_2_'s peak performance may stem from pre-embedded Mg^2+^, imparting a pre-magnetization tendency.^183^

Building on structural modifications, Yu *et al.* introduced a facile heterojunction of the VO_2_·*x*H_2_O@V_2_O_5_ (VO_*x*_) electrode with a nanobelt structure, synthesized *via* electrochemical deposition for AMgIBs. This biphasic structure integrates the advantages of layered V_2_O_5_ and tunnel-like VO_2_·*x*H_2_O, delivering excellent storage capacity and cycle stability. The heterojunction enhances interlayer spacing, facilitating transport pathways for Mg^2+^ and H^+^. The VO_*x*_ electrode yields reversible capacities of 510, 356.7, 307.6, 270.3, 243.2, 220.5, and 195.5 mAh g^−1^ at 0.05, 0.1, 0.2, 0.5, 1, 2, and 5 A g^−1^, respectively. Upon returning to 0.05 A g^−1^, the electrode sustains a capacity of 315.7 mAh g^−1^.^184^

Complementing these efforts, Vasić *et al.* investigated magnesium vanadium oxides (MVOs), such as MgV_2_O_6_ and multiphase systems, prepared *via* simple solid-state synthesis, as cathodes for rechargeable AMgIBs. Structural characterization *via* XRD, FTIR, and Raman spectroscopy revealed layered structures with varying phases. Electrochemical performance, assessed by cyclic voltammetry (CV), showed sluggish Mg^2+^ kinetics in MgV_2_O_6_ due to poor electronic/ionic conductivity, as confirmed by bond valence sum (BVS) calculations indicating limited diffusion pathways. Complex redox behavior depended on phase composition and inserted ions (Mg^2+^/H^+^). Multiphase MVOs outperformed single-phase ones in Mg^2+^ insertion/deinsertion. Carbon addition enhanced charge storage *via* dual electronic conductivity and structural stabilization. The MgV_2_O_6_/carbon composite delivered improved capacities (∼100–150 mAh g^−1^ at 50 mV s^−1^) and stability, highlighting a strategy to mitigate kinetic limitations in AMgIBs.^185^

Zuo *et al.* synthesized the MVO microspheres through a simple hydrothermal process and then calcined them. The 4 nm microspheres are composed of 10 nm nanoparticles, as shown in Fig. 18D and E, and the EDS (Fig. 18C) shows that the Mg, V, and O elements are uniformly distributed. The material has good reaction kinetics according to the CV curves, as shown in Fig. 18A. The team analyzed the storage mechanism, as shown in Fig. 18B; during the initial charge process, Mg^2+^ is extracted from MVO which transforms into (Mg_0.5_V_1.5_)O_4_. During the discharge process, Mg^2+^ intercalates into (Mg_0.5_V_1.5_)O_4_ and turns back to Mg(Mg_0.5_V_1.5_)O_4_.^186^ In addition, Huang *et al.* showed us that the proton co-embedding in the tunnel material is determined by both interface derivation and internal diffusion, as shown in Fig. 18F–H.^187^ Tian and his team synthesized the ultra-thin VO_*x*_ nanosheet, as shown in Fig. 18I–J, which can achieve 402 mAh g^−1^ at 0.13 A g^−1^.^188^ Nano-morphologies shorten Mg^2+^ diffusion paths to accelerate kinetics, yet cycling life remains inadequate. Ma *et al.* leveraged the anisotropy of layered V_3_O_7_·1.9H_2_O nanobelts to stabilize the structure during discharge/charge. V_3_O_7_·1.9H_2_O, a typical layered vanadium oxide with mixed +4/+5 valences, comprises V_3_O_8_ layers of [VO_6_] octahedra and [VO_5_] trigonal bipyramids, yielding two distinct Mg^2+^ diffusion directions. The synthesized V_3_O_7_·*n*H_2_O exhibits (200) lattice planes spaced at 8.4 Å. Nanobelts measure 100–300 nm in width and over 100 µm in length. TEM shows lattice fringes of 0.84 nm, matching the (200) interplanar spacing of V_3_O_7_ crystals. It delivers 137 mAh g^−1^ at 0.05 A g^−1^ (Fig. 18 K–O) (Table 5).^189^

**Fig. 18:**
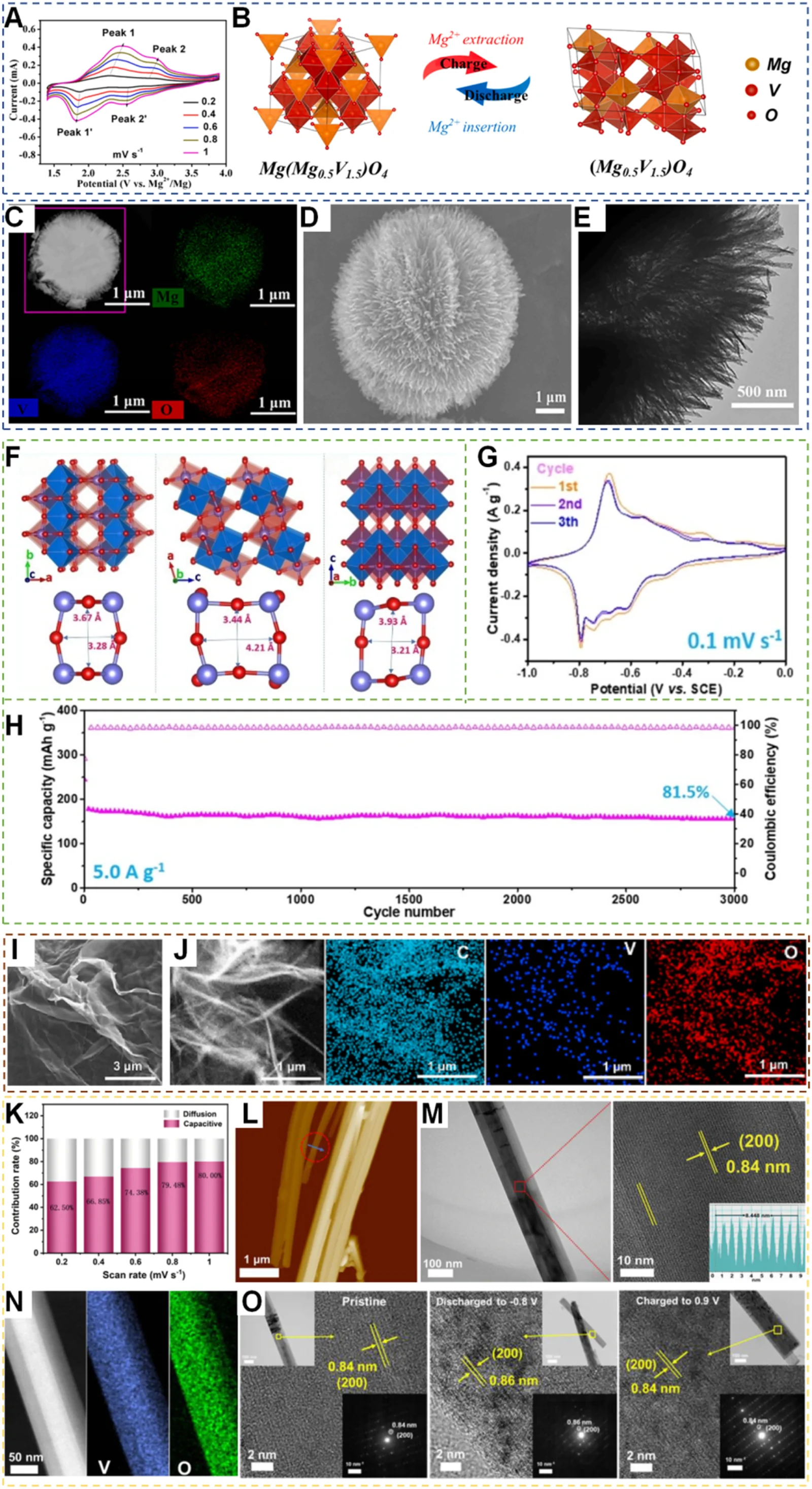
(A) CV curves of MVO at 0.2–1 mV s^−1^. (B) Mg^2+^ intercalation/deintercalation schematic during discharge/charge. (C) MVO TEM-EDS maps. (D) MVO SEM image. (E) MVO TEM image.^186^ (F) VO_2_(B) structures along the *c*/*b*/*a*-axes. (G) CV curves of VO_2_(B) at 0.1 mV s^−1^. (H) Long-term cycling at 5 A g^−1^.^187^ (I) VO_*x*_ SEM image. (J) VO_*x*_ EDX mapping (C, V, and O).^188^ (K) Capacitive ratios for V_3_O_7_·1.9H_2_O at various scan rates. (L) V_3_O_7_·1.9H_2_O AFM image. (M) V_3_O_7_·1.9H_2_O TEM images. (N) V_3_O_7_·1.9H_2_O STEM/EDS mapping (V and O). (O) *Ex situ* HRTEM image: pristine V_3_O_7_·1.9H_2_O nanobelts, with discharging at −0.8 V and charging at 0.9 V.^189^ Adapted from ref. 186, 188 and 189 with permission from Elsevier, copyright 2021, 2023 and 2025, respectively; and ref. 187 with permission from Wiley-VCH, copyright 2023.

**Table 5:** Performance comparison of various vanadium-based cathodes for AMgIBs

Material	Synthesis	Performance	Ref.
V_2_O_5_ nanostructures	Chemical vapor deposition	82% retention after 2000 cycles	181
Mg_0.3_V_2_O_5_·1.1H_2_O	Hydrothermal intercalation	80% retention after 10 000 cycles	60
BL-HVO_4_ NPS	Hydrothermal	Stable over 2000 cycles	182
Mg_0.21_VO_2_	Hydrothermal pre-intercalation	62.5% retention after 1000 cycles	183
VO_*x*_ heterojunction	Electrodeposition	100 mAh g^−1^ after 1000 cycles	184
V_3_O_7_·1.9H_2_O	Hydrothermal	11 000 cycles with 60% retention	189
MgV_2_O_5_/C	Precipitation & carbon composite	Improved stability in the composite	185

Overall, AMgIB cathode design is dominated by the need to maintain water-assisted Mg^2+^ transport and suppress irreversible structural rearrangement. Hydrated V_2_O_5_ derivatives and Mg^2+^/H_2_O pre-intercalated frameworks directly address the desolvation and migration barriers, whereas VO_2_-based and heterojunction electrodes improve kinetics by expanding channels and creating additional interfacial pathways. Compared with Zn^2+^ systems, AMgIBs require stronger emphasis on the role of interlayer water and on separating genuine Mg^2+^ storage from proton-assisted or surface-dominated contributions.

## 6. Cross-system comparison and charge-carrier-dependent design principles

### 6.1 Common mechanistic features across vanadium-based aqueous cathodes

Across Zn^2+^, NH_4_^+^, Al^3+^, and Mg^2+^-based aqueous batteries, vanadium-based cathodes share several mechanistic foundations. Charge storage relies on the accessible multivalent redox chemistry of vanadium, typically involving V^5+^/V^4+^/V^3+^ couples. Reversible cycling requires stabilization of V–O or V–X frameworks to prevent irreversible phase reconstruction, dissolution of active vanadium species, and loss of electrical connectivity. All systems suffer, to different extents, from strain accumulation during repeated ion insertion/extraction. Intercalation engineering, defect regulation, surface modification, and heterostructure construction are broadly useful, yet their function and priority depend strongly on the charge carrier.

### 6.2 Carrier-dependent differences in storage chemistry and material design

The major differences among these aqueous systems originate from charge density, hydration structure, desolvation energy, and host-interaction mode. These factors serve as the intrinsic determinants governing both the energy storage mechanism and the direction of materials design. Such intrinsic differences systematically lead to disparities among aqueous battery systems in terms of reaction pathways, kinetic limitations, and the priorities of modification strategies, rather than merely resulting in distinct performance metrics (Table 6).

**Table 6:** Charge-carrier-dependent storage mechanisms and design principles for vanadium-based aqueous cathodes

Carrier	Ion feature	Dominant storage mechanism	Main challenge	Preferred design
Zn^2+^	Divalent ion; strong Zn–O interaction	Zn^2+^/H^+^ co-insertion; pseudocapacitive contribution	V dissolution and by-product formation	Interlayer water/ion pre-intercalation; surface protection; controlled defect engineering
NH_4_^+^	Monovalent molecular ion; hydrogen bond donor	Hydrogen-bond-assisted insertion	Limited energy density	Flexible layered structure; polymer intercalants; abundant hydrogen-bond acceptor sites
Al^3+^	Trivalent ion; high charge density and strong hydration	Al^3+^/H^+^ co-storage; pseudocapacitive contribution	Slow diffusion and by-product formation	Open structure; amorphous/defect phase; conductive heterostructure
Mg^2+^	Divalent ion; strong hydration	Mg^2+^/H^+^ co-storage and water-assisted interlayer diffusion	Difficult to desolvate; slow migration	Hydrated layered oxides; Mg^2+^/H_2_O pre-intercalated frameworks; heterostructures

### 6.3 Cross-system comparison of representative vanadium-based material families

The cross-system applicability of vanadium-based materials in different systems is determined by the matching degree of their intrinsic structures (layered, tunnel-like, multi-anion framework, and high conductivity network) and the physical and chemical properties of charge carriers. The same vanadium-based material may exhibit different mechanisms in different aqueous batteries and show regular changes with the characteristics of charge carriers, which reflects the intrinsic coordination relationship between the structure and mechanism (Table 7).^190^

**Table 7:** Cross-system comparison of representative vanadium-based material families

Material family	AZIBs	AIBs	AAlIBs	AMgIBs	General conclusion
V_2_O_5_/hydrated V_2_O_5_	High capacity	Dependent on hydrogen bonds and flexible interlayer connections	Requires defect/composite structure assistance	Mg^2+^ diffusion requires interlayer water assistance	The layered structure is universal, but the interlayer environment needs to be regulated according to the carriers
	Easy to dissolve				
VO_2_	Low-temperature vanadium activation can occur	Defect/hydration structure is advantageous	The opening of VO_2_(B) channels is beneficial	Requires pre-embedding ion expansion layer	Crystal form and oxidation state determination mechanism
Vanadates	Good structural stability	NH_4_V_4_O_10_ is suitable for hydrogen bond storage	Can serve as a host for Al^3+^	The dynamics of Mg^2+^ are still restricted	Pre-embedded ions can serve as structural pillars
Sulfides/nitrides	High conductivity, often accompanied by reconfiguration	Less research	The conversion reaction needs to be controlled	Still in the early stage	More suitable for constructing heterostructures with carbon/MXene

## 7. Conclusions and prospects

Vanadium-based cathode materials have emerged as pivotal components in the development of aqueous rechargeable batteries, including AZIBs, AAlIBs, AMgIBs, and AIBs, owing to their multivalent redox activity (V^2+^–V^5+^), diverse crystal architectures (layered, tunnel-type, and three-dimensional frameworks), and high theoretical capacities. This review systematically summarizes recent advances in vanadium oxides, vanadates, phosphates, sulfides, nitrides, and their composites across these aqueous battery systems, with particular emphasis on material modification strategies, such as ion/water intercalation, defect engineering, surface/interface regulation, and heterostructure construction, and their roles in regulating energy-storage mechanisms. These approaches have been developed to address intrinsic limitations of aqueous systems, including narrow electrochemical stability windows (∼1.23 V), sluggish multivalent-ion diffusion, and cathode dissolution, thereby advancing vanadium-based cathodes toward practical energy-storage applications.

Historically, vanadium-based cathode materials were first explored in non-aqueous ion batteries, where their layered structures and multi-electron redox behavior enabled outstanding electrochemical performance, particularly in lithium-ion batteries. However, driven by lithium resource constraints and growing demand for sustainable energy storage, researchers have increasingly applied vanadium-based materials into aqueous environments. In AZIBs, early studies employed V_2_O_5_ cathodes that delivered initial capacities of ∼200–300 mAh g^−1^ but suffered from rapid capacity fading due to dissolution and structural instability.^150,191^ Subsequent interlayer-engineered materials, such as Zn_0.25_V_2_O_5_·*n*H_2_O, marked a transition toward enhanced cycling stability and higher reversible capacities. In AAlIBs, early V_2_O_5_ cathodes enabled Al^3+^ intercalation but exhibited limited capacities (∼100–200 mAh g^−1^) due to strong electrostatic interactions and sluggish kinetics.^192^ AMgIBs and AIBs emerged thereafter, leveraging the structural tunability of vanadium frameworks for Mg^2+^ and NH_4_^+^ storage, with initial research primarily focused on overcoming kinetic barriers. Since then, the field has rapidly evolved from simple oxides toward advanced composites and heterostructured materials, reflecting broader efforts to meet the demands of large-scale and sustainable energy storage.

Parallel to material evolution, synthesis strategies have progressed from conventional solid-state, sol–gel, and hydrothermal routes toward more sophisticated and controllable techniques. Early hydrothermal methods enabled the fabrication of layered V_2_O_5_ and vanadates with basic nanostructures but offered limited control over defects and surface chemistry. Advanced approaches, such as plasma-assisted hydrothermal synthesis, van der Waals self-assembly, and *in situ* anodic oxidation, have enabled the construction of water-intercalated nanosheet clusters with enlarged interlayer spacing (*e.g.*, ∼1.23 nm), heterostructures (*e.g.*, V_2_O_5_@MXene), and oxygen-deficient composites (*e.g.*, Od-VO@C) that improve conductivity and suppress dissolution. MOF-derived strategies have produced mesoporous architectures (*e.g.*, C@VO_2_@V_2_O_5_ with ∼126 m^2^ g^−1^ surface area), while defect engineering through doping (*e.g.*, Cu-doped VO_2_ nanoflowers) and aerosol-assisted CVD has enabled precise regulation of vacancies and polymorphs. These synthesis routes, often combined with guest pre-intercalation (*e.g.*, PVP and Ti^4+^/K^+^), have significantly enhanced structural stability, electronic transport, and scalability.

As a result of these material and mechanistic advances, the electrochemical performance of vanadium-based cathodes has improved substantially. In AZIBs, capacities have increased from ∼200–300 mAh g^−1^ with limited cycling stability to ∼400–700 mAh g^−1^ through intercalation engineering, defect modulation, and conductive composites (*e.g.*, Od-VO@C reaching ∼700 mAh g^−1^).^88^ In AAlIBs, vanadium-based cathode capacities have progressed from ∼120 mAh g^−1^ to above 400 mAh g^−1^ by vacancy regulation and MXene-based composites that enhance Al^3+^ kinetics and mitigate structural degradation. AMgIBs initially suffered from slow Mg^2+^ diffusion and modest capacities (∼100–200 mAh g^−1^), while heterojunction engineering and pre-intercalation strategies have boosted capacities to ∼500 mAh g^−1^, with ∼100 mAh g^−1^ retained after 1000 cycles. AIBs exhibit analogous trends, with NH_4_^+^-intercalated vanadates achieving ∼200 mAh g^−1^ and improved reversibility. Additionally, electrochemical activation, electrolyte optimization, and internal electric-field regulation, shown by DFT to reduce adsorption energies to −1.2 to −2.44 eV, have further enhanced rate performance.

Despite this progress, several fundamental challenges remain across AZIBs, NH_4_^+^-IBs, AMgIBs, and AAlIBs, limiting commercial viability. In AZIBs, poor capacity retention at low current densities persists, primarily due to aggravated vanadium dissolution and secondary side reactions. Broader barriers include narrow electrolyte ESWs, zinc anode corrosion, dendrite growth, and cathode dissolution. Current strategies have evolved from simple interlayer ion insertion toward synergistic approaches combining defect engineering and composite design, including Prussian-blue-type assemblies.^193–196^ However, long-term stability under practical operating conditions remains unresolved.

In NH_4_^+^-IBs, practical deployment is hindered by the lack of low-potential anodes and by cathode dissolution in aqueous electrolytes, leading to structural degradation and rapid capacity decay. While intercalation and vacancy engineering have improved diffusion kinetics and capacity, further development of vanadium nitrides and sulfides as cathode materials represents a promising future direction.^139,141,197,198^

For AMgIBs, energy density is constrained by the low ESW (1.3–2.0 V) of aqueous magnesium electrolytes and the scarcity of high-performance cathodes. Although Mg^2+^ incorporation and heterostructure design have yielded improvements, future progress will depend on developing open three-dimensional frameworks, composite metal systems, and surface-dominated storage mechanisms to overcome intrinsic kinetic limitations.^199^

In AAlIBs, the high charge density of Al^3+^ leads to severe electrostatic interactions, slow diffusion kinetics, and pronounced structural damage of vanadium-based cathodes. Moreover, the formation of insoluble by-products during cycling causes interfacial blockage and structural collapse. Future AAlIB research should concentrate on enhancing three-electron transfer efficiency through multifunctional cathodes with tunable polymorphic structures, defect-rich or amorphous phases, and compatible mixed anodes (*e.g.*, aluminum–graphene systems).^163,164,172,200^

Future studies should move from general performance optimization toward carrier-directed cathode design. For Zn^2+^ systems, the priority should be electrostatic shielding, surface protection, and suppression of vanadium dissolution. For NH_4_^+^ systems, the key direction is the construction of reversible hydrogen-bond networks through flexible layered hosts, interlayer water, and polymer or molecular intercalants. For Al^3+^ systems, the focus should shift to open, defect-rich, amorphous, or surface-dominated architectures that avoid forcing deep Al^3+^ insertion into rigid lattices. For Mg^2+^ systems, hydrated diffusion channels and Mg^2+^/H_2_O pre-intercalated frameworks should be emphasized to lower desolvation and migration barriers.

Electrolyte–cathode co-design is equally important. Electrolyte composition controls pH, solvation structure, vanadium dissolution, by-product formation, and the extent of H^+^ participation; therefore, cathode performance cannot be evaluated independently from electrolyte chemistry. Dynamic reconstruction should also be treated as a central research target, especially for low-valence vanadium materials such as VO_2_, V_2_O_3_, VN, and VNO, which may oxidize, hydrate, amorphize, or reconstruct during cycling. Future work should distinguish the activity of the pristine material from that of the reconstructed active phase.

Mechanistic verification should rely more heavily on *operando* and online methods, including *operando* XRD, *in situ* Raman spectroscopy, X-ray absorption spectroscopy, online ICP, electrochemical quartz crystal microbalance, pH mapping, and ToF-SIMS, to track phase evolution, vanadium valence, dissolution, interfacial mass changes, and ion distribution. In parallel, DFT, molecular dynamics, and data-driven screening can be used to predict ion adsorption energies, diffusion barriers, defect formation energies, interlayer-water stability, and vanadium dissolution tendency. These approaches would make future material selection less empirical and more mechanistically predictive.

Overall, future research on vanadium-based aqueous batteries should transition from empirical optimization toward mechanism-guided, system-level design, integrating multifunctional hybrid materials, scalable and environmentally benign synthesis routes, and theoretical modeling to rationally optimize ion transport, redox reversibility, and structural stability. The development trends and remaining challenges for different aqueous battery systems are summarized schematically in Fig. 19. This review provided a useful reference and mechanistic framework for advancing vanadium-based cathode materials toward sustainable and practical aqueous energy-storage technologies.

**Fig. 19:**
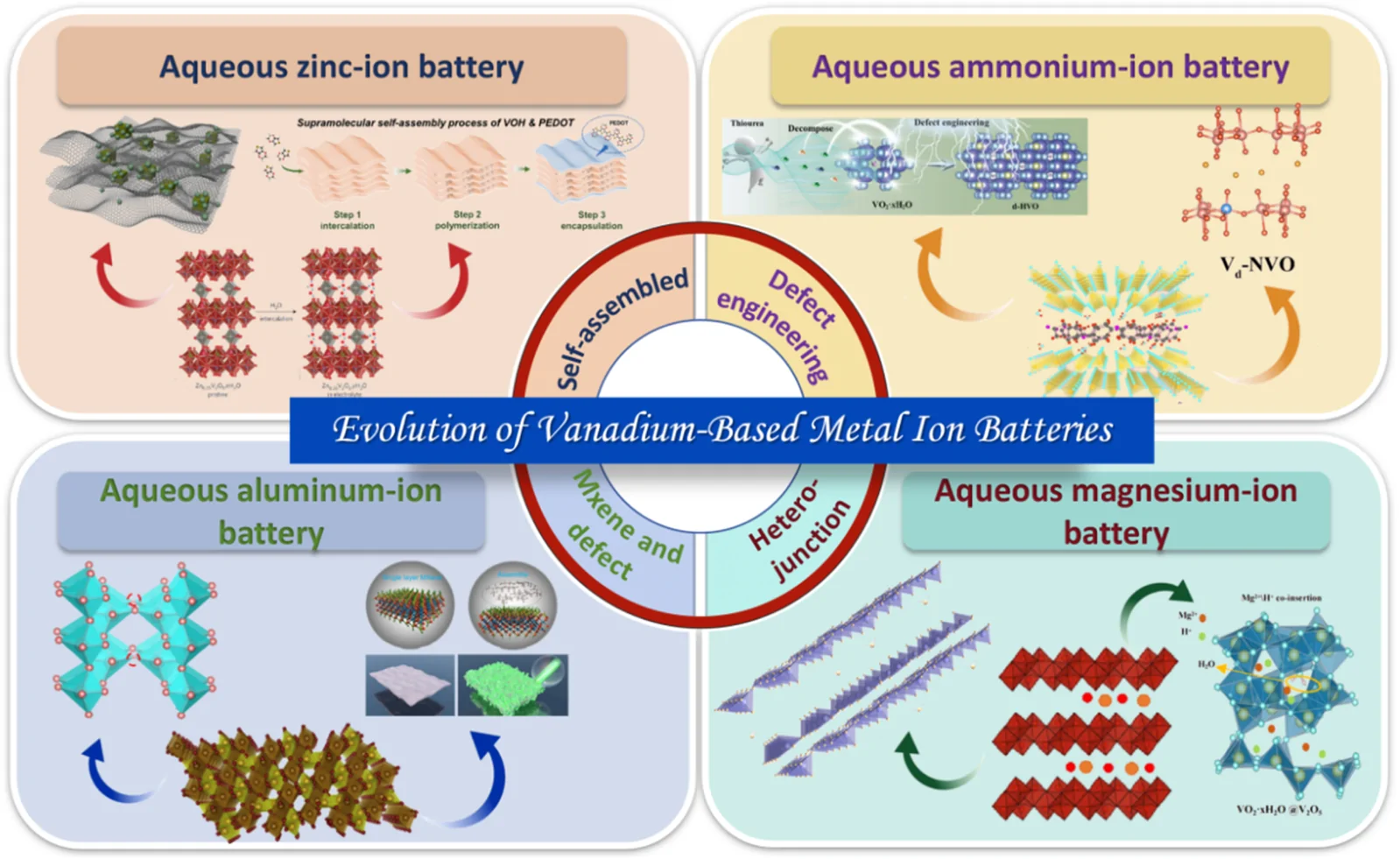
Overview of the future outlook image.^58,133,136,146,163,164,174,182,184,188,201,202^ Adapted from ref. 58 with permission from Springer Nature, copyright 2016; ref. 133, 136, 164, 174, 182, 188 and 201 with permission from Elsevier, copyright 2022, 2024, 2025, 2019, 2024, 2023 and 2025, respectively; ref. 146 with permission from Springer Nature, copyright 2024; ref. 163 and 184 with permission from the American Chemical Society, copyright 2024 and 2025, respectively; and ref. 202 with permission from the Royal Society of Chemistry, copyright 2025.

## Author contributions

Xunxiang Jia and Xiaoyue Zhang conceived and designed the review, collected and analyzed the literature, prepared the original draft, and wrote the manuscript. Xiangyang Li contributed to literature collection, data organization, figure preparation and manuscript revision. Wenhaotian Zhu, Xinxin Cui and Weiji Li contributed to literature collection, data organization and manuscript revision. Mengchuan Lai, Zekai Zhu and Yumeng Lu provided literature support, data collection and suggestions for improving the manuscript. Xuefeng Ren, Xifei Li and Chong Peng contributed suggestions and improvements to the review and reviewed and edited the manuscript. Anmin Liu managed the project, acquired funding, supervised the work, and reviewed and edited the manuscript.

## Conflicts of interest

There are no conflicts to declare.

## Data Availability

No primary research results, software or code have been included and no new data were generated or analysed as part of this review.
